# Adult skin fibroblast state change in murine wound healing

**DOI:** 10.1038/s41598-022-27152-4

**Published:** 2023-01-17

**Authors:** Fatma Z. Gharbia, Ahmed S. Abouhashem, Yomna A. Moqidem, Ahmed A. Elbaz, Ahmed Abdellatif, Kanhaiya Singh, Chandan K. Sen, Hassan M. E. Azzazy

**Affiliations:** 1 Graduate Nanotechnology Program, The American University in Cairo (AUC), AUC Avenue, P.O. Box 74, New Cairo, 11835 Egypt; 2 Indiana Center for Regenerative Medicine & Engineering, Department of Surgery, Indiana University School of Medicine, Indianapolis, IN 46202 USA; 3 Department of Chemistry, School of Sciences & Engineering, The American University in Cairo (AUC), AUC Avenue, P.O. Box 74, New Cairo, 11835 Egypt; 4 Sharkia Clinical Research Department, Ministry of Health & Population, Zagazig, 44511 Sharkia Egypt; 5 CytoTalk LLC, Cheyenne, WY 82001 USA; 6 Department of Biology, School of Sciences & Engineering, The American University in Cairo (AUC), AUC Avenue, P.O. Box 74, New Cairo, 11835 Egypt; 7 Department of Nanobiophotonics, Leibniz Institute for Photonic Technology, Albert Einstein Str. 9, 07745 Jena, Germany; 8 Present Address: Department of Medicinal Chemistry, College of Pharmacy, University of Michigan, Ann Arbor, MI 48105 USA

**Keywords:** Computational biology and bioinformatics, Medical research

## Abstract

Wound healing is a well-organized dynamic process involving coordinated consecutive phases: homeostasis, inflammation, proliferation and resolution. Fibroblasts play major roles in skin wound healing such as in wound contraction and release of growth factors which are of importance in angiogenesis and tissue remodeling. Abnormal fibroblast phenotypes have been identified in patients with chronic wounds. In this work, we analyzed scRNA-seq datasets of normal and wounded skin from mice at day 4 post-wound to investigate fibroblast heterogeneity during the proliferative phase of wound healing. Compositional analysis revealed a specific subset of fibroblast (cluster 3) that primarily increased in wounded skin (14%) compared to normal skin (3.9%). This subset was characterized by a gene signature marked by the plasma membrane proteins *Sfrp2* + *Sfrp4* + *Sfrp1* + and the transcription factors *Ebf1* + *Prrx1* + *Maged1* + . Differential gene expression and enrichment analysis identified epithelial to mesenchymal transition (EMT) and angiogenesis to be upregulated in the emerging subset of fibroblasts of the wounded skin. Using two other datasets for murine wounded skin confirmed the increase in cluster 3-like fibroblasts at days 2, 7 and 14 post-wounding with a peak at day 7. By performing a similarity check between the differential gene expression profile between wounded and normal skin for this emerging fibroblast subset with drug signature from the ConnectivityMap database, we identified drugs capable of mimicking the observed gene expression change in fibroblasts during wound healing. TTNPB, verteprofin and nicotinic acid were identified as candidate drugs capable of inducing fibroblast gene expression profile necessary for wound healing. On the other hand, methocarbamol, ifosfamide and penbutolol were recognized to antagonize the identified fibroblast differential expression profile during wound healing which might cause delay in wound healing. Taken together, analysis of murine transcriptomic skin wound healing datasets suggested a subset of fibroblasts capable of inducing EMT and further inferred drugs that might be tested as potential candidates to induce wound closure.

## Introduction

The wound healing process consists of four overlapping phases: hemostasis, inflammation, proliferation and resolution^1,2^. Immediately after wounding, the hemostasis phase begins with formation of fibrin clots and vascular constriction. Next, migration of inflammatory cells into the wound starts. Recruitment of inflammatory cells such as neutrophils, macrophages and lymphocytes initiate the inflammatory phase of the wound healing process^3^. The proliferation phase overlaps with the inflammatory phase in which epithelial cells proliferate and migrate within the wound area^4^. During the proliferative phase, fibroblasts and endothelial cells are the most prominent cell types which support collagen formation, blood vessel growth and granulation tissue formation at the injury site^4^. After proliferation and extracellular matrix synthesis are completed, the healing process enters the final resolution phase in which tissue remodeling occurs^4^. During the resolution phase, the wound density returns to normal by pruning of many of the newly formed blood vessels^5^. During the wound healing process, activation of well-organized biological processes, such as epithelial to mesenchymal transition (EMT), mesenchymal to epithelial transition (MET) and angiogenesis, occurs^6,7^. During the EMT process, epithelial cells acquire mesenchymal characteristics, such as enhanced motility, change their interactions with ECM and lose their cell–cell junctions^8–11^. The EMT process, which is necessary for normal embryonic development, is accelerated during the wound healing process^8,12^. The EMT was also found to be accelerated by fibroblast growth factor 2 during wound healing^13^. Next, MET helps to reconstitute the apical junctional complex (AJCs) restoring the barrier function of the repaired skin. Additionally, angiogenesis increases during the healing process^14^. New capillary sprouts invade the wound clot followed by their organization into a network through the granulation tissue before their regression during the resolution phase of wound healing^4^.

Fibroblasts are diverse and dynamic in nature and play major role in wound healing^15^. As it relates to wound contraction, they are present in two distinct states: contractile and noncontractile^16^. Fibroblasts are activated and differentiated into myofibroblasts at the wound site to generate contractile forces to bring the wound edges together and facilitate wound closure^16^. The EMT process plays significant role in myofibroblast transition^16,17^. Fibroblasts also support the wound angiogenesis process by the production of appropriate extracellular matrix (ECM) bed^18^. Fibroblast malfunction has been reported to cause wound chronicity^19^. These awry fibroblasts show impaired pattern of cytokine release and display abnormal phenotypes with defective proliferation and early senescence^20^. Fibroblast-focused therapies for wound care are currently under development^21,22^. Growth factor-based therapies such as platelet derived growth factor B subunit B seek to stimulate fibroblasts activation and differentiation^23,24^. However, the focus on any single growth factor to heal wound has not been productive^25^. The complexity of the in vivo wound healing microenvironment requires intelligent approaches to modify fibroblast state as would be needed for wound closure.

Defining Fibroblast heterogeneity is an important and emerging area of research in wound healing. The application of single cell RNA sequencing (scRNA-seq) has established different states (e.g. contractile, regenerative, adaptive or fibrotic) of skin fibroblasts during development, homeostasis and injury in both human and murine models^26–33^. A recently published article provided a systematic view of skin cellular dynamics at day 4 during dermal wound healing in mice at the single cell level^34^. In the current study, we investigated the same dataset (GSE142471) to identify fibroblast states during wound healing and to change normal skin fibroblast states necessary for physiological wound healing. Next, we verified the presence of the identified states using other datasets (GSE153596, GSE178758 and GSE188432). Such understanding will inform therapies targeting the change of fibroblast state in non-healing wound. The primary outcomes of this study include: (1) identification of skin cell types states in normal skin and during wound healing, (2) identification of the fibroblast state emerging during the wound healing process, (3) identification of the differentially expressed genes, altered pathways and biological processes in fibroblasts during wound healing, and (4) identification of the drugs which could induce changes in fibroblast gene expression consistent with that required for physiological wound repair.

## Results

**Figure 1:**
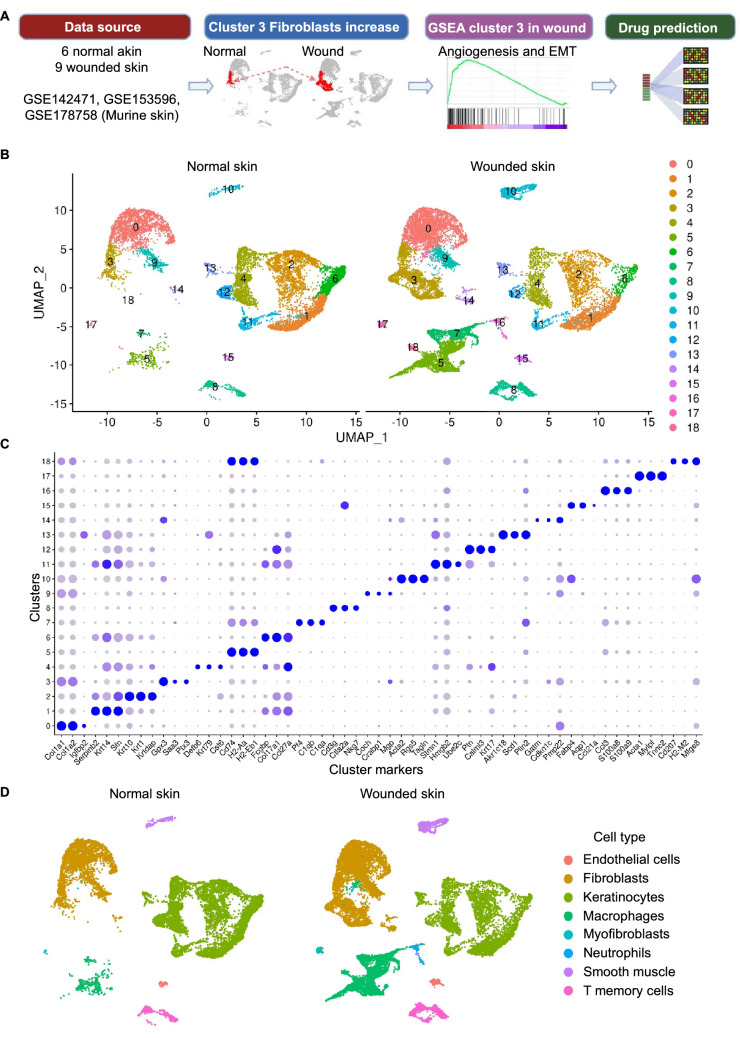
Identification of 19 distinct clusters. (**A**) The analysis overview. (**B**) UMAP plot showing 19 distinct clusters. Each dot represents a single cell. Left panel represents normal skin cells and right panel represents wounded skin at day 4 post wounding (GSE14271). (**C**) Dotplot representing the top 3 markers in each cluster. (D) UMAP plots showing eight cell types for each condition (normal skin and wounded skin).

Murine skin scRNA-seq data were retrieved from GEO database (accession no. 142471) from two groups: normal skin and day 4 wounded skin. Cells were clustered and cell types were identified for each of the identified 19 clusters. Clusters identified as fibroblasts were analyzed to identify the subset of fibroblasts having higher abundance during wound healing. Differential expression analysis and Gene Set Enrichment Analysis (GSEA) were performed on the identified subset of fibroblasts (cluster 3) which revealed upregulation of angiogenesis and EMT. The resulting DEGs were used to identify drugs which have potential to induce fibroblast state transition from the resting state found in cluster 3 in normal skin towards the wound healing state found in cluster 3 in wounded skin (Fig. 1a).

### Identification of eight distinct cell types in murine skin

The initial data included 27,317 cells from 5 skin samples (2 normal skin samples and 3 skin samples at day 4 post wounding). After excluding low quality cells, 26,723 cells remained for the downstream analyses (Supplementary Fig. 1; Supplementary table 1). Clustering of single cells identified 19 distinct clusters (Fig. 1b,c). Cell type assignment identified eight cell types including keratinocytes (11,124 cells), fibroblasts (8,657 cells), macrophages (3,971 cells), T memory cells (1,214 cells), smooth muscle cells/ pericytes (868 cells), endothelial cells (319 cells), neutrophils (307 cells), myofibroblasts (263 cells) (Fig. 1c,d; Supplementary Fig. 2; Supplementary table 2).

### Identification of wound healing-related genes and biological processes in the skin tissue

The transcriptional changes that occurred in skin at day 4 of wound healing were investigated by comparing genes from all cells in wounded skin and all cells from the normal skin samples (Supplementary table 3). Enrichment analysis using GSEA software identified affected pathways and biological processes in the wounded skin. The upregulated gene sets in skin during wound healing included granulocyte chemotaxis, neutrophil chemotaxis, IL6 Jak Stat3 signaling, IL18 signaling, myogenesis and EMT (Supplementary Fig. 3a and b; Supplementary table 4). Sixty genes were found to be upregulated with a log2FC > 0.25 involved in EMT. The top upregulated genes involved in epithelial mesenchymal transition included *Cxcl1*, *Acta2*, *Thbs1*, *Timp1*, *Tpm1*, *Tpm2*, *Il6*, *Basp1* and *Vim* (Supplementary Fig. 3c). Genes involved in granulocytes chemotaxis in wounded skin included *Cxcl2*, *Ccl4*, *Ccl3*, *Cxcl1*, *S100a9*, *Ccl2*, *S100a8*, *Cd74*, *Thbs1*, *Fcer1g*, *Ccl7*, *Ccl8*, *Rac2*, *Ccl11*, *Dpep1*, *Pde4b* and *Rarres2* with log2FC > 0.25 (Supplementary table 4). On the other hand, keratinization, cornification and keratinocyte differentiation were found to be downregulated in wounded skin (Supplementary table 4).

### Identification of a subset of fibroblasts during wound healing with upregulated angiogenesis and EMT

**Figure 2:**
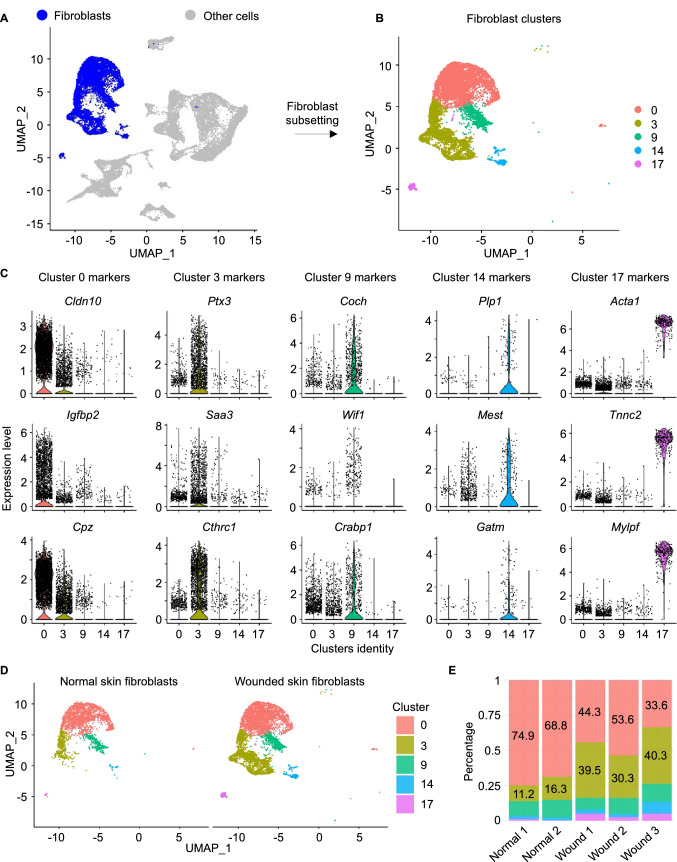
Identification of distinct subsets of fibroblasts. (**A**) UMAP plot showing cells identified as fibroblasts or myofibroblasts in blue color and other cells in grey color. (**B**) Fibroblast clusters. (**C**) Top upregulated 3 genes in fibroblast clusters 0, 3, 9, 14 and 17. (**D**) UMAP plots showing cells identified as fibroblasts splitted by condition (normal skin: left panel and wounded skin: right panel). (E) Barplot representing percentage of each fibroblast cluster among the fibroblast population within each sample.

**Figure 3:**
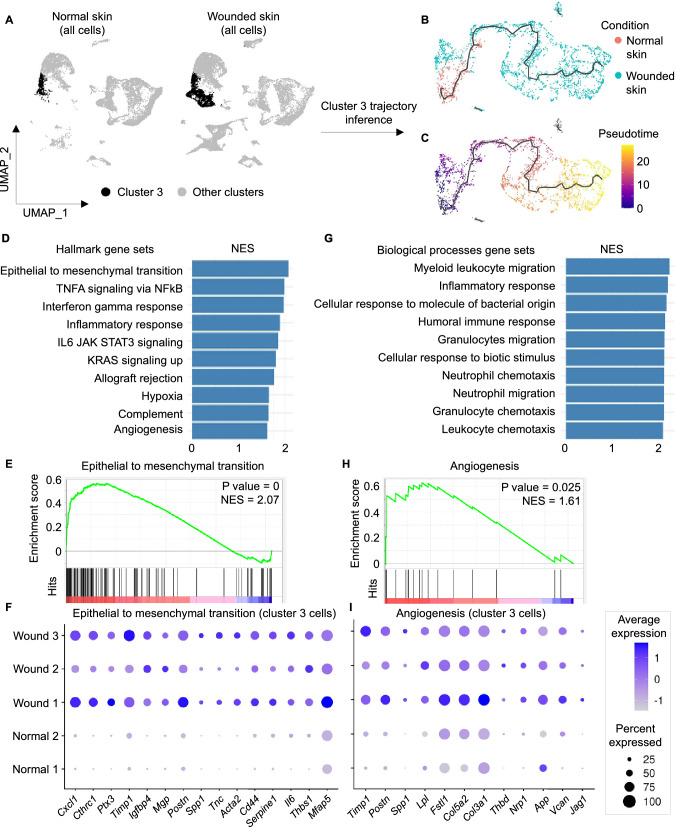
Upregulation of EMT and angiogenesis in cluster 3 fibroblasts at day 4 post-wound in mice skin. (**A**) UMAP plot representing cluster 3 in a black color and other clusters in s grey color. (**B**, **C**) UMAP plots of cluster 3 cells colored by condition and pseudotime receptively. The black line represents the inferred trajectory. (**D**, **G**) Top 10 uppregulated hallmark and biological processes gene sets in cluster 3 in wounded skin compared to normal skin. Bars represent the normalized enrichment score (NES) of the gene set enrichment analysis. (**E**, **H**) GSEA enrichment plot for EMT and angiogenesis. Hits (black lines) represent the intersection between the DEGs and the enriched gene sets. (**F**, **I**) Dotplots representing relative expression level of the top upregulated genes having role in EMT and angiogenesis respectively with log2FC > 0.25 in cluster 3 during wound healing compared to cluster 3 in normal skin. Dot size represents percentage of cells within cluster 3 expressing the gene in each sample. Dot color represents the relative expression level of the gene.

To identify the role of fibroblasts in the wound healing process, analysis of fibroblast clusters was performed. Cells identified as fibroblasts were found in five distinct states (clusters 0, 3, 9, 14 and 17) with specific upregulated markers for each (cluster 0: *Igfbp2*, *Cpz* and *Cldn10*; cluster 3: *Saa3*, *Ptx3* and *Cthrc1*; cluster 9: *Coch*, *Crabp1* and *Wif1*; cluster 14: *Gatm*, *Plp1* and *Mest*; cluster 17: *Acta1*, *Mylpf* and *Tnnc2*) (Fig. 2a–c). Percentage of cells in clusters 0, 9 and 14 among all cell types was comparable between normal skin and wounded skin (cluster 0 cells: 18% in normal skin and 17.12% in wounded skin; cluster 9: 3.16% in normal skin and 3.98% in wounded skin; cluster 14: 0.53% in normal skin and 1.65% in wounded skin). On the other hand, cluster 3 had a higher percentage of cells in wounded skin relative to in normal skin, increasing from 3.93% to 14.09% (Supplementary table 1). To test if this increase in cluster 3 cells resulted from an increase in proliferation or differentiation, cell cycle analysis was performed using the function *CellCycleScoring* in Seurat to identify the ratio of dividing cells and non-dividing cells in cluster 3 from normal skin and wounded skin. The canonical marker genes for S, G2 and M phases were used to calculate cell cycle scores for each cell as reported previously^35^. Next, cells expressing markers for either S, G2 or M phases were assigned to proliferative cells, while cells not expressing mitotic marker genes were assigned to G1 phase. Analysis of cell cycle phase for cluster 3 cells identified that 54.3% and 55.15% of cells were assigned to G1 phase (non-proliferative cells) in normal skin and wounded skin respectively indicative of no proliferation differences. Also, myofibroblasts (cluster 17) increased from 0.1% to 1.56%. Fibroblasts in cluster 3 represent 11.2–16.3% of the fibroblast population in normal skin, while they represent 30.3–40.3% of the fibroblast population in wounded skin (Fig. 2d,e, Supplementary table 1). Trajectory inference of cluster 3 cells identified a trajectory capturing cluster 3 cells from both normal skin and wounded skin samples (Fig. 3a–c). Comparing genes between cluster 3 cells in wounded skin and normal skin resulted in identification of 1,650 DEGs with adjusted p value < 0.05. Among them, 506 genes were found to be upregulated and 282 genes were found to be downregulated using a cutoff log2FC ± 0.25. The resulting DEGs were classified based on their family into transcription factors, plasma membrane proteins, enzymes and other categories. The top upregulated plasma membrane proteins in cluster 3 in wounded skin included *Sfrp2* and *Sfrp4*, while the top downregulated plasma membrane proteins included *Il1r2* and *Ghr* (Supplementary Fig. 4). The transcription factors *Nfkbia* and *Noct* were upregulated, while *Fosp*, *Ebf1*, *Klf4* and *Aff3* were downregulated in wounded skin cluster 3. Regarding growth factors, *Ptn* and *Mdk* were upregulated, while *Gas6* and *Igf1* were downregulated in wounded skin cluster 3. All DEGs in cluster 3 of wounded skin samples are provided in supplementary table 5. Enrichment analysis using GSEA software resulted in identification of altered biological processes and pathways in cluster 3 in wounded skin compared to cluster 3 in normal skin (p value < 0.05 and FDR q-val < 0.1) (Fig. 3d; Supplementary table 6). The upregulated gene sets included epithelial mesenchymal transition (Fig. 3e,f), granulocyte chemotaxis (Fig. 3g) and angiogenesis (Fig. 3h,i). On the other hand, epidermal development, keratinocyte differentiation and oxidative phosphorylation were found to be downregulated in cluster 3 fibroblasts during wound healing (Supplementary table 6). To investigate the contribution of other fibroblast clusters (0,9,14 and 17), in this paradigm, we compared inter-cluster transcriptome profiles (Supplementary Fig. 5a and b). A total of 1,235 genes were found to be differentially expressed in fibroblast clusters when comparing each cluster from wounded skin with the corresponding cluster in normal skin with log2FC ± 0.25 and adjusted p value < 0.05. Among these, 255 genes were upregulated, and 105 genes were downregulated in more than one fibroblast cluster (Supplementary table 7). Other genes were differentially expressed in a cluster-specific basis (483 upregulated genes and 323 genes). A total of 69 genes had bidirectional behavior (upregulated and downregulated in distinct fibroblast clusters). Upregulated genes in wounded skin fibroblast clusters compared to normal skin that have role in EMT included 16 genes in cluster 0, 63 genes in cluster 3, 35 genes in cluster 9, 20 genes in cluster 14 and no genes in cluster 17 (Supplementary Fig. 6).

### Validation of cluster 3-like cells in other datasets

**Figure 4:**
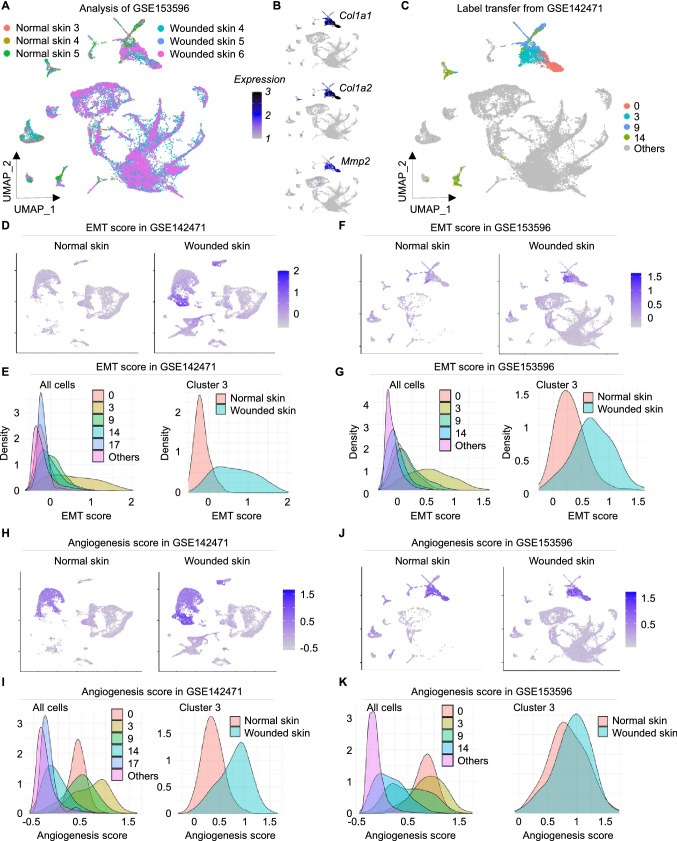
Upregulation of EMT and angiogenesis in cluster 3 fibroblasts at day 4 post-wound in mice skin. (**A**) UMAP representing cells from 3 normal skin and 3 wounded skin samples at day 7 post-wound retrieved from GSE153596. (**B**) Expression level of Col1a1, Col1a2 and Mmp2. (**C**) UMAP plot representing cells from GSE153596 labeled using mutual nearest neighbor and label transfer (see methods) from GSE142471. (**D**) EMT score in cells from GSE142471. (**E**) Left panel represents EMT score in fibroblast clusters and other cell types. Right panel represents EMT score in cluster 3-like cells from GSE142471 in normal and wounded skin. (**F**) EMT score in cells from GSE153596. (**G**) Left panel represents EMT score in fibroblast clusters and other cell types. Right panel represents EMT score in cluster 3-like cells from GSE153596 in normal and wounded skin. (**H**-**I**) Angiogenesis score in cells from GSE142471 in all cells and in cluster 3 fibroblasts. (**J**-**K**) Angiogenesis score in cells from GSE153596 in all cells and in cluster 3 fibroblasts.

Since our goal was to identify a fibroblast population that uniquely represented the ability to support EMT during wound healing, we focused on cluster 3 in our subsequent study. The dataset with accession number GSE153596 included 34,859 cells from 6 samples (3 normal skin and 3 wounded skin) at day 7 post-wound was thus retrieved and investigated for presence and quantification of cluster 3 cells. After excluding low quality cells 34,191 cells remained for downstream analysis among which 5,669 cells were labeled as fibroblasts similar to clusters 0, 3, 9 and 14 from the original dataset with high expression of *Col1a1*, *Col1a2* and *Mmp2* (Fig. 4a–c). Cells labeled as cluster 3 have higher EMT scores in both GSE153596 and GSE142471 compared to other fibroblast clusters and other cell types. Additionally, cluster 3 cells in wounded skin have higher EMT scores in wounded skin at day 4 and day 7 compared to normal skin (Fig. 4d–g). Angiogenesis scores were found to be higher in clusters 0, 3 and 9 in both GSE153596 and GSE142471 datasets (Fig. 4h–k). Next, to quantitate the abundance of cluster 3 fibroblasts at different days post-wounding, another dataset with accession number GSE178758 was investigated. This dataset included 19,968 cells from 4 samples at different time points post-wounding (days 0, 2,7 and 14). Cluster 3-like cells increased from 5.7% in normal skin to 27.7%, 40,8% and 26.6% in days 2, 7 and 14 respectively (Supplementary Fig. 7a, b and c). In conclusion, combining all three datasets it became clear that cluster 3 significantly increased post wounding in murine skin (p = 0.0003), while cluster 0 showed no significant alteration as compared to normal skin (p value = 0.99). Furthermore, the expression of EMT genes in these cluster 3-like cells remain low in the wound of aged mice (88 week old) as compared to young mice (7 week old) (adjusted p value < 0.05, supplementary Fig. 8).

### Identification of drugs affecting the wound healing process

**Figure 5:**
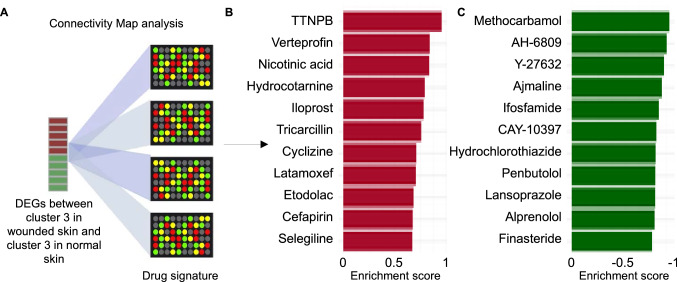
Identification of drugs mimicking or counteracting the differential expression pattern in cluster 3 during wound healing. (**A**) The similarity between the DEGs between cluster 3 cells in wounded skin and cluster 3 cells in normal skin with log2FC ± 0.25 were checked with the signature of each of the 6,100 drug/ compound instances in the Connectivity Map database. (**B**) The top identified drugs having signature that mimics the differential expression pattern in cluster 3 in wounded skin (positive enrichment). (**C**) The top identified drugs having signature that counteracts the differential expression pattern in cluster 3 in wounded skin (negative enrichment).

Prediction of drugs using Connectivity Map resulted in identification of 21 drugs with positive and 40 drugs with negative enrichment scores (P value < 0.05). Scores were based on the similarity between the effect of the drug on gene expression profile from the Connectivity Map database^36^ (drug signature) and the observed alteration in gene expression level in cluster 3 cells in wounded skin compared to cluster 3 in normal skin. The top 3 drugs with positive scores included TTNPB, verteporfin, and nicotinic acid (predicted to be capable of inducing fibroblast state change from the normal skin state towards the identified state in cluster 3 cells during wound healing). On the other hand, the top 3 drugs with negative scores included methocarbamol, AH-6809 and Y-27632 (predicted to be capable of inducing fibroblast state transition towards the resting state found in cluster 3 in normal skin) (Fig. 5a–c; Supplementary table 8).

## Methods

### Data retrieval

scRNA-seq data was extracted from the gene expression omnibus (GEO) database ( GSE142471, GSE153596 and GSE178758). Those datasets were selected as they included murine small wound 2-6 mm at 2, 4, 7 and 14 post wound days. Besides, the mice in the included data have no diseases and received no treatments representing wound healing under normal physiological conditions. For the wound healing experiments in GSE142471, the authors of the original study used 5 mice skin samples where they performed scRNA-seq experiments using 7 week-old female *K14-Cre; ROSA*^*mTmG*^ mice (C57BL/6 J background)^34^. They generated a full-thickness wound in the back skin of 3 mice using a 6-mm punch. Four days later, they excised the wound and the surrounding unwounded skin regions (1.5 cm in diameter) using a 10-mm punch. For the control group (2 mice), they shaved the mice, removed the back skin, and scrapped off the fat. Finally, they minced the skin from both groups into pieces of less than 1 mm in diameter where they were used for library generation using the Chromium Single Cell 3′ Reagents Kits v2. HiSeq 4000 platform was utilized to sequence samples and Cell Ranger 2.1.0. was used to map the resulting FASTQ reads to mm10 genome.

### Single-cell RNA sequencing preprocessing

Seurat package (4.0.4) in R (v.4.1.1) was used for all the analyses except mentioned^37–41^. Firstly, the initial data in GSE142471, including a total of 27,317 cells from the 5 samples (2 normal skin samples and 3 skin samples during wound healing) was log normalized using 10,000 as a scaling factor for the total number of detected molecules per cell. Filtration criteria was determined to exclude low quality cells. Cells with less than 200 expressed genes, a total number of detected molecules less than 500 were excluded or more than 30,000 were excluded and cells with more than 10% of their genes originating from the mitochondrial genome were excluded. All downstream analyses were performed using the remaining 26,723 cells. After removing low quality cells, the top 2,000 genes exhibiting high cell-to-cell variation were identified for each sample. Next, highly variable genes were ranked based on their identification as variable genes in multiple samples and were used for integration of the 5 samples using canonical correlation analysis (CCA). To reduce the data dimensions, we performed a principal component analysis (PCA) and the top 25 principal components were chosen for dimensionality reduction and visualization using the UMAP algorithm.

### Clustering of single cells and cell types assignment

Clustering of single cells was performed using a graph-based clustering approach. Next, the Louvain algorithm was applied to group cells together with different resolutions and a resolution of 0.25, which represents distinct states among each cell type in this dataset, was selected for downstream analysis. Cluster markers were identified by comparing genes from each of the resulting 19 clusters with the rest of the cells combined using the Wilcoxon Rank Sum test. For a gene to be considered for comparison, it has to be expressed in at least 20% of the cells of either the cluster or in 20% of the rest of the cells. Cell type assignment was performed using the PanglaoDB database using the top 10 upregulated genes in each cluster^42^. Cell type annotation was performed automatically using the PanglaoDB database, while manual annotation was performed for clusters which was not automatically assigned.

### Differential expression analysis

To identify the effect of the wound healing process on gene expression profile of skin tissue, gene expression values were compared between all cells from the wounded skin and all cells from the normal skin. Wilcoxon Rank Sum Test was performed to identify the differentially expressed genes (DEGs) and for a gene to be considered for the comparison, it has to be expressed in at least 20% of cells in either of the 2 groups. Additionally, genes were compared between cluster 3 cells in wounded skin and normal skin, which were identified to have a higher percentage of cells in wounded skin samples compared to normal skin. Identified DEGs from all analyses were annotated using ingenuity pathways analysis (IPA) software^43–45^. In addition, 4 other comparisons were performed to compare between other fibroblasts subsets from wounded skin and normal skin in clusters 0, 9, 14 and 17. The resulting DEGs for all fibroblasts comparisons were classified into genes differentially expressed in one fibroblast subset, genes having similar change in multiple subsets and genes with bidirectional change (upregulated and downregulated in distinct fibroblast clusters).

### Gene set enrichment analysis

The resulting DEGs from comparing all cells in wounded skin with all cells in normal skin and between cluster 3 cells in wounded skin with normal skin with adjusted p value < 0.05 were considered for enrichment analysis. Enrichment was performed using the GSEA software using the default parameters^46,47^. Enrichment was performed against hallmark gene sets and gene ontology biological processes gene sets. The genes identified to be involved in EMT from this analysis were checked for their differential expression in other fibroblast clusters 0, 9, 14 and 17 (Supplementary Fig. 6).

### Trajectory inference for cluster 3 cells

Fibroblasts identified in cluster 3 from normal skin and wounded skin were used to infer a trajectory linking between them. Monocle3 package (v 1.0.0) in R was used to infer a trajectory based on the continuous change in gene expression profiles in cluster 3 cells^48,49^. PCA analysis was performed and the first 10 dimensions were used to reduce the data dimensions.

### Cell cycle analysis

To test if the increase in cluster 3 cells resulted from an increase in proliferation or differentiation, cell cycle analysis was performed using the function *CellCycleScoring* in Seurat to identify the ratio of dividing cells and non-dividing cells in cluster 3 cells from normal skin and wounded skin. The canonical marker genes for S, G2 and M phases were used to calculate cell cycle scores for each cell^35^. Next, cells expressing markers for either S, G2 or M phases were assigned to proliferative cells, while cells not expressing mitotic marker genes were assigned to G1 phase.

### Analysis of wound healing at day 7 post-wound

To verify the presence of cluster 3-like cells in wounded skin from other datasets, the dataset with GEO accession number GSE153596 was retrieved including 3 samples of normal skin and 3 samples of 2 mm wounded skin at day 7 post-wound in 21-day old mice^27^. Cells with more than 200 detected genes, between 500 and 30,000 total number of counts and less than 10% mitochondrial DNA expression were kept for downstream analysis. All samples were integrated using CCA and log normalization was performed with a scaling factor of 10,000. Next, PCA was performed to reduce data dimensions and UMAP was used for visualization. The functions *FindTransferAnchors* and *Transfer Data* in Seurat were used to map the cluster labels from the original dataset (GSE142471) into the new samples based on mutual nearest neighbors. The function *AddModuleScore* in Seurat was used to calculate scores for EMT and angiogenesis using the identified upregulated genes in cluster 3 in wounded skin.

### Identification of cluster 3-like cells over time during wound healing

To further identify the dynamics of cluster 3 cells, we retrieved a spatial transcriptomic dataset (GSE178758) of wound healing at different time points (normal skin, day 2, 7 and 14 post wound)^29^. The authors performed splinted excisional 6 mm diameter wound in mice skin. Cells with more than 200 detected genes, between 500 and 30,000 total number of counts and less than 10% mitochondrial DNA expression were kept for downstream analysis. All samples were integrated using CCA and log normalization was performed with a scaling factor of 10,000. Then, PCA was performed to reduce data dimensions and UMAP was used for visualization. SingleR and celldex packages in R were used to identify fibroblasts based on Spearman correlation across marker genes between each cell and the reference samples^50^. Cell type assignment was computed for each cell against the reference labels including sorted 18 main cell types^51^. Cluster labels from GSE142471 were used to identify cells similar to cluster 3 cells using the functions *FindTransferAnchors* and *TransferData* in Seurat.

### Analysis of wound healing in young and aged mice

To identify the effect of aging in cluster 3-like cells proportion and the accompanied EMT activation, 2 combined count matrices were retrieved from GSE188432 representing young and aged skin samples^52^. The data included normal skin and wounded skin from 7 young and 3 aged mice skin samples. The authors generated a full-thickness wound using 6-mm punch in 7-week-old and 88-week-old C57BL/6 J mice. Cells with more than 200 detected genes, between 500 and 30,000 total number of counts and less than 20% mitochondrial DNA expression were kept for downstream analysis (46,713 cells). All samples were integrated using CCA and log normalization was performed with a scaling factor of 10,000. Then, PCA was performed to reduce data dimensions and UMAP was used for visualization. Cluster labels from GSE142471 were used to identify cells similar to cluster 3 cells using the functions *FindTransferAnchors* and *TransferData* in Seurat. The function *AddModuleScore* in Seurat was used to calculate scores for EMT in young and aged skin samples. Additionally, *FindMarkers* function in Seurat was used to compare the EMT-related genes in cluster 3-like cells between aged and young mice.

### Predictions of drugs for fibroblast shate change

To identify which drugs or compounds have an effect on gene expression profile mimicking that observed change in cluster 3 fibroblast in wounded skin compared to normal skin, a similarity check was performed using the Connectivity map software^36^. Connectivity map is a large public database of drugs and gene signatures which represent the effect of drugs on gene expression profile. The basic concept of Connectivity map is to compare between drug-specific gene expression profiles as references with a specific gene signature by submitting a list of upregulated and downregulated genes relevant to a particular biological condition. The connectivity map database includes 6,100 microarray experiments of 1,309 drugs on different cell lines such as MCF7, HL60 and PC3 and varying concentrations and time points against vehicle controls. The fold change of treatment to control was calculated and sorted into decreasing order and converted to a ranked list. DEGs with adjusted p value < 0.05 and log2FC ± 0.25 resulting from comparing genes from cluster 3 in wounded skin and normal skin were annotated with HT_HG-U133A IDs as a requirement for Connectivity Map enrichment. Next, the annotated genes were submitted as a signature query in Connectivity Map software to perform the enrichment. The query was compared to the ranked vector based on the fold changes of the probesets. The algorithm gives a connectivity score either positive or negative for each instance using a nonparametric rank-ordered Kolmogorov–Smirnov (KS) test. Next, the resulting connectivity scores were normalized using random permutation, so values close to + 1 reflects closeness or similarity between the submitted query and the effect of the drug on the gene expression profile using the ranked list. A score close to -1 emphasizes an inverse similarity between the submitted query and the reference profile. The algorithm detected the similarity between drug signatures from the database and the differential expression pattern in cluster 3 fibroblasts to unravel unexpected connections between them.

## Discussion

Understanding the dynamic cell biology of wound healing process in vivo in light of scRNA-seq data will provide critical insight into the molecular basis of wound closure. Fibroblasts have numerous roles in skin wound healing including deposition of new ECM of murine skin^53^. We analyzed scRNA-seq data of mice skin in normal state and during wound healing with the objective to identify and characterize the subset of fibroblasts playing a major role in the wound healing process. To be able to identify drugs that could induce fibroblasts to enable wound healing, genes from the emerging subset of fibroblasts in wound versus those in normal skin were compared followed by a similarity check between the resulting differential gene expression pattern and the drug effects on gene expression profile.

Compositional analysis identified a distinct percentage of cell types in normal and wounded skin. The observed early upregulation of neutrophil chemotaxis is consistent with the reported sequel of events wherein early recruitment of neutrophils is followed by monocytes which differentiate into wound macrophage^54,55^. Fibroblasts were identified in different states based on distinct gene expression profiles. This is consistent with previous reports that dermal fibroblasts include processes relevant to wound healing^54,56,57^. Among the identified fibroblast subsets, the increase in cluster 3 percentage from 3.93% in normal skin to 14.09% among all cells in wounded skin hints towards their potential role in the wound healing process. The top upregulated plasma membrane proteins found in wounded skin cluster 3 fibroblasts were *Sfrp2*, *Sfrp4* and *Sfrp1*. *Sfrp2* has been found to be essential for myocardial tissue survival and repair by enhancing engraftment of mesenchymal stem cells and formation of granulation tissue^58,59^. On the other hand, downregulation of *Gpc4* (Glypican 4) induces cell migration and proliferation in breast cancer cell lines^60^. Upregulation of EMT process in cluster 3 fibroblasts during wound healing is consistent with previous reports^13,61^. During the EMT process, epithelial cells acquire mesenchymal features such as enhanced motility, change their interaction with ECM and undergo cytoskeletal rearrangement^8–11^. *Cxcl1*, *Cthrc1*, *Ptx3* genes support EMT and were upregulated in cluster 3 wound fibroblasts. *Cxcl1* promote EMT in cutaneous wound healing by mediating formation of new vessels^62,63^. *Cthrc1* improves wound healing via regulating NOTCH and TGF-β pathways and recruiting M2 macrophages^64^. The deficiency of *Ptx3* has pathological consequences such as epithelial hyperplasia, excessive accumulation of collagen and defective mature tissue formation^65^. Data analysis presented in this work identified multiple genes involved in angiogenesis to be upregulated in cluster 3 fibroblasts in wounded skin including *Fstl1* and *Nrp1*. *Fstl1* (Follistatin Like 1) is a known angiogenic factor that promotes ischemic tissue revascularization and endothelial cell function^66^. *Nrp1* (Neuropilin 1) is critically important to drive angiogenesis^67^. Anti-*Nrp1* treatment exhibited a significant reduction in the wound vascular density (67% decrease) during dermal wound healing^67^. Investigation of cluster 3 wound fibroblasts suggest their involvement in EMT and angiogenesis during the wound healing process. EMT upregulation was found to be cluster 3-specific, while angiogenesis score was found to be higher in clusters 0, 3 and 9 indicating that multiple subsets of fibroblasts are involved in the angiogenesis process. Cluster 3 cells were found to have a similar proportion of proliferative and non-proliferative cells in both normal skin and wounded skin indicating that the observed increase in cluster 3 cells resulted from cell differentiation. This indicates that some other mechanisms rather than proliferation might be involved in the rise of cluster 3 cells during wound healing. Trajectory inference also explained such differentiation of cluster 3 fibroblasts in normal skin (resting state) into cluster 3 fibroblasts in the wound healing state. These observations lead to the hypothesis that cluster 3 cells present in uninjured skin differentiate to the observed state during wound healing activating EMT.

Wound healing outcomes may be viewed as a product of the acquisition of appropriate cell state and fate by participating cell types. Such state and fate of any single cell depends on their gene expression profiles. In this work the differential gene profile of cluster 3 fibroblast can be considered as the signature profile requirement for physiological wound healing. Identification of drugs with the ability to induce pro-healing signature gene expression profile will help populate a drug candidate list for preclinical and clinical validation studies. The result of the similarity check compared to the signature of cluster 3 wound fibroblasts revealed a group of drugs with positive enrichment scores (pro-healing) and another group with negative enrichment scores (anti-healing). Anti-healing drugs will interfere with the healing process and should be useful to induce wound chronicity for experimental processes. Among the pro-healing drugs, TTNPB, an analog of retinoic acid, activates retinoic acid receptors potently and selectively^68^. TTNPB in combination with other small molecules has been reported to enable chemical reprogramming of mice embryonic fibroblasts to pluripotent stem cells^69^. Verteporfin induces skin regeneration without scar formation through *Yap* (Yes-associated protein) inhibition^70^. Additionally, verteporfin was found to inhibit fibrogenic genes and was suggested to be a novel antifibrotic agent^71^. The third drug identified to have a positive enrichment score was nicotinic acid which represents the active form of vitamin-B3. Nicotinic acid was reported to have anti-inflammatory and antioxidant effects^72–74^. Furthermore, nicotinic acid topical administration was found to improve tissue regeneration in excisional skin wounds through the increment of fibroblast proliferation, collagen synthesis, and vascularization^75,76^. Findings of this study provide a list of candidate drugs that should be preclinically and clinically tested for their effects on improving wound closure. In conclusion, findings of this work at transcriptome level underscore the diversity and dynamicity of wound-site fibroblasts and their potential responsiveness to drugs that may induce their change of state favoring wound closure. However, the results are solely based on analysis of transcriptomic datasets and further mechanistic studies are needed to support its inferences.

## Supplementary Information

Supplementary Information 1.

Supplementary Information 2.

Supplementary Information 3.

## Data Availability

The datasets are publicly available in the National Center for Biotechnology Information Gene Expression Omnibus (GEO) repository (GSE142471, GSE153596, GSE178758 and GSE188432).
