# Supplementary material for: Adult skin fibroblast state change in murine wound healing
Source: Sci Rep. 2023 Jan 17;13:886. doi: 10.1038/s41598-022-27152-4 (PMC9845335; doi:10.1038/s41598-022-27152-4)
Supplement: Supplementary file 2 — Supplementary Information 2. [file 41598_2022_27152_MOESM2_ESM.pdf]

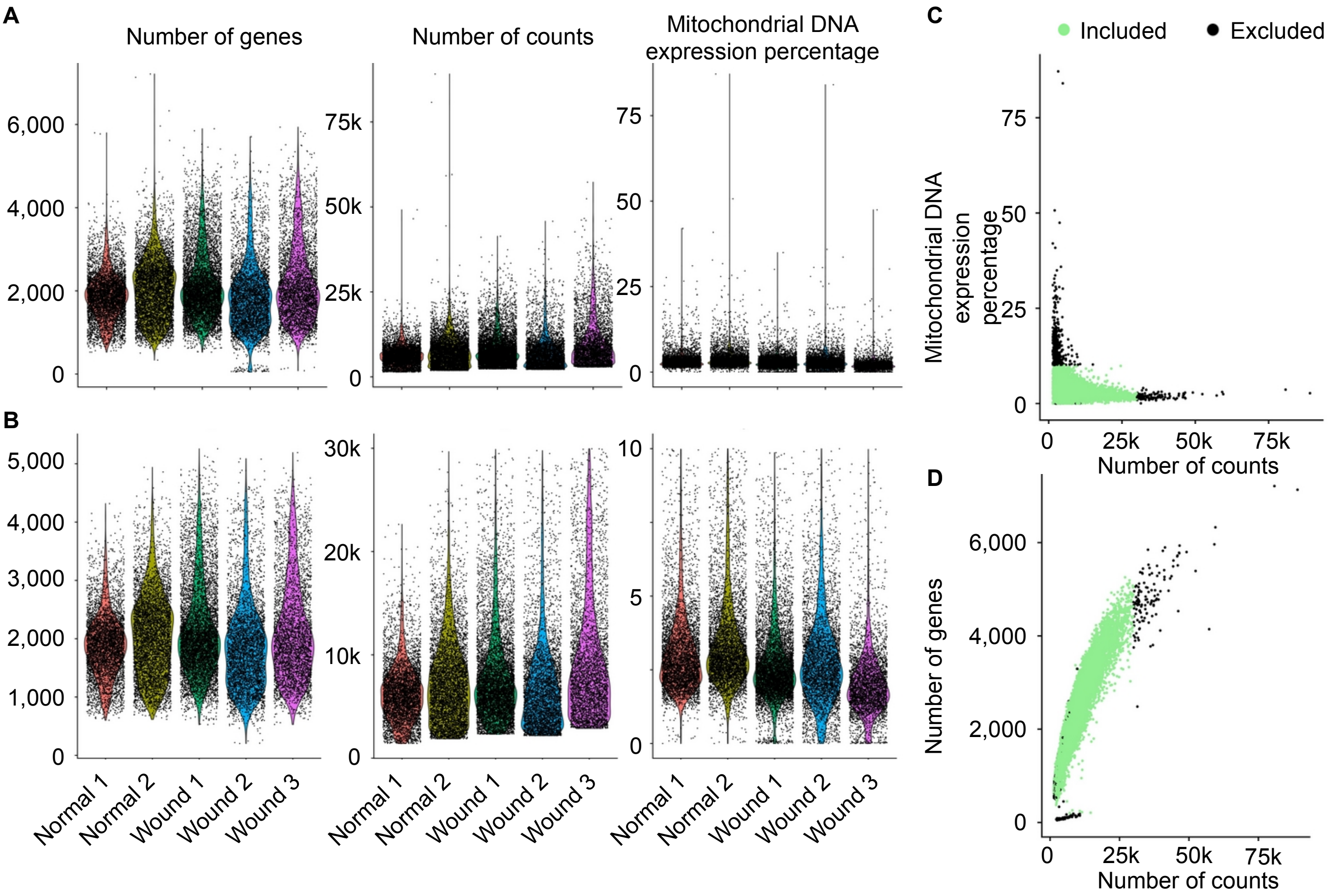

Supplementary fig 1. Filtering low quality cells in GSE142471. (A and B) Violin plots showing the distribution of the total number of detected genes (left), total number of detected molecules (middle) and the percentage of mitochondrial DNA expression (right) in the five samples before and after excluding low quality cells respectively. Each dot represents a single cell. (C and D) Scatter plot of the total number of counts versus mitochondrial DNA expression percentage and total number of detective genes respectively. Each dot represents a single cell. Green dots represent cells included in the downstream analyses, while black dots represent cells which didn't pass the quality control criteria as mentioned in methods.

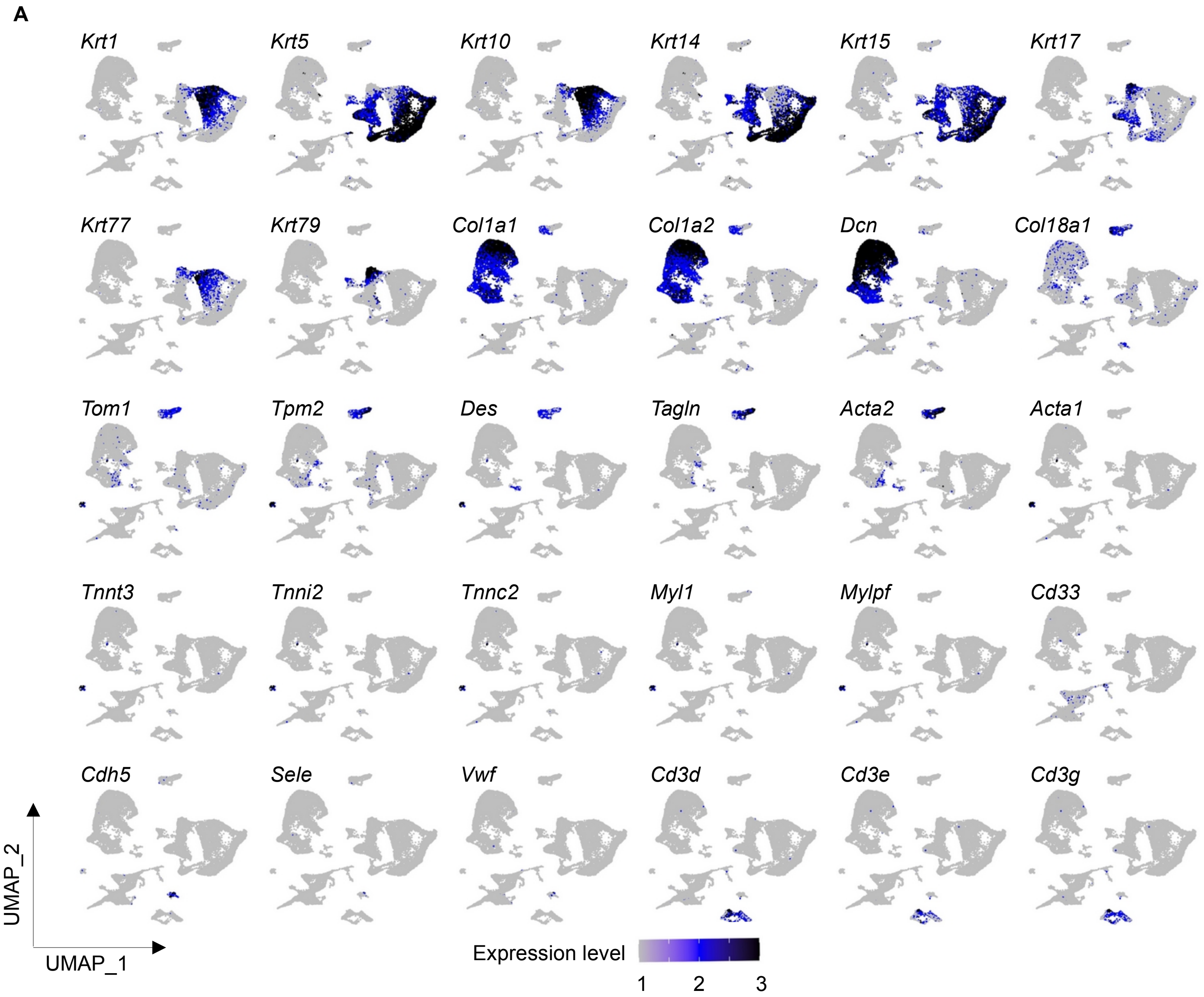

Supplementary fig 2. Cluster markers in GSE142471. (A) UMAP plots showing expression level of cell type markers.

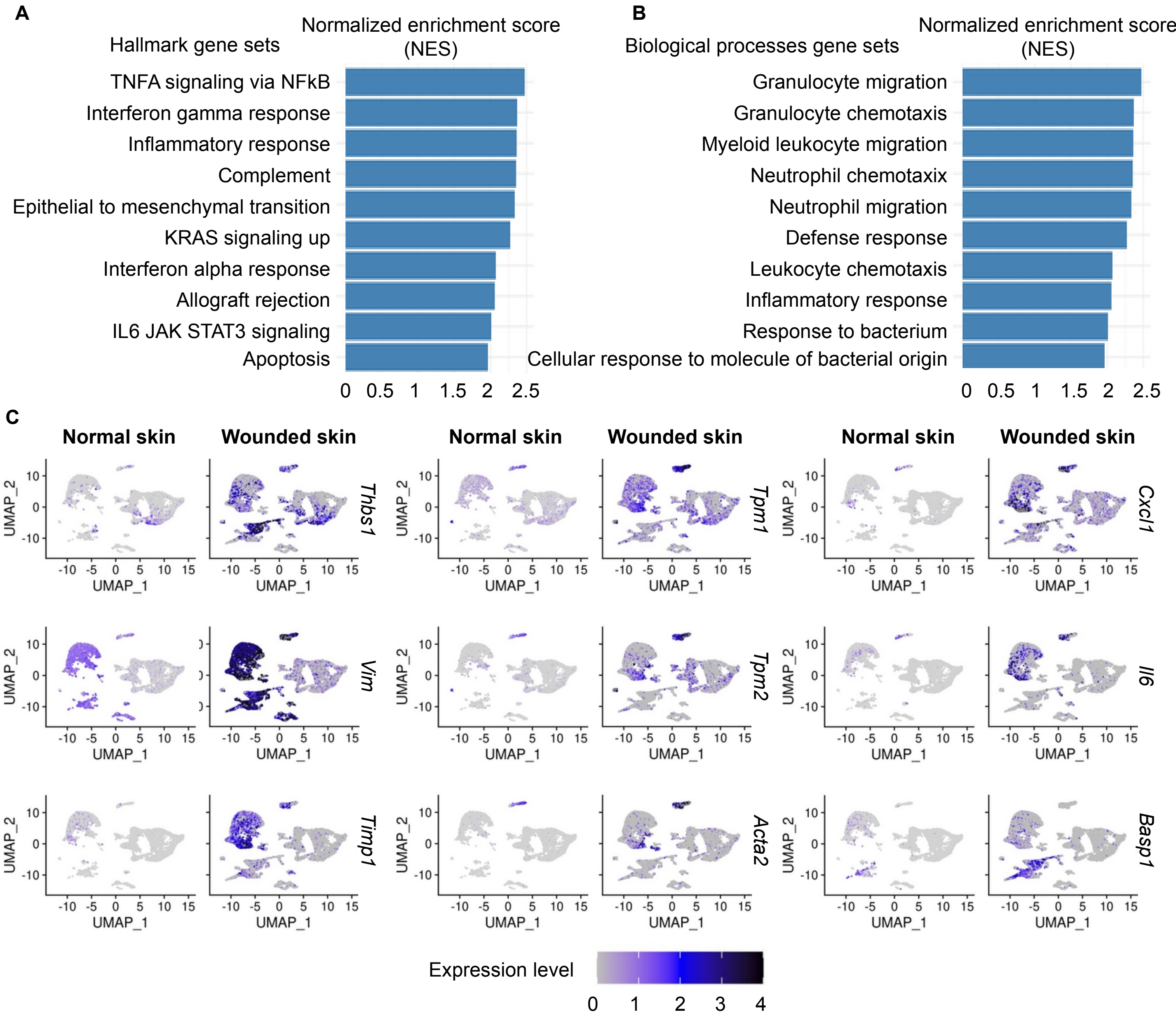

Supplementary fig 3. Upregulation of EMT in wounded skin tissue at day 4 post wounding from GSE142471. (A & B) Top 10 upregulated hallmark and biological processes gene sets in all cells in wounded skin compared to normal skin. Bars represent the normalized enrichment score of the gene set enrichment analysis. (C) UMAP plots for all cells representing top nine upregulated genes in wounded skin having a role in EMT. Each dot represents a single cell.

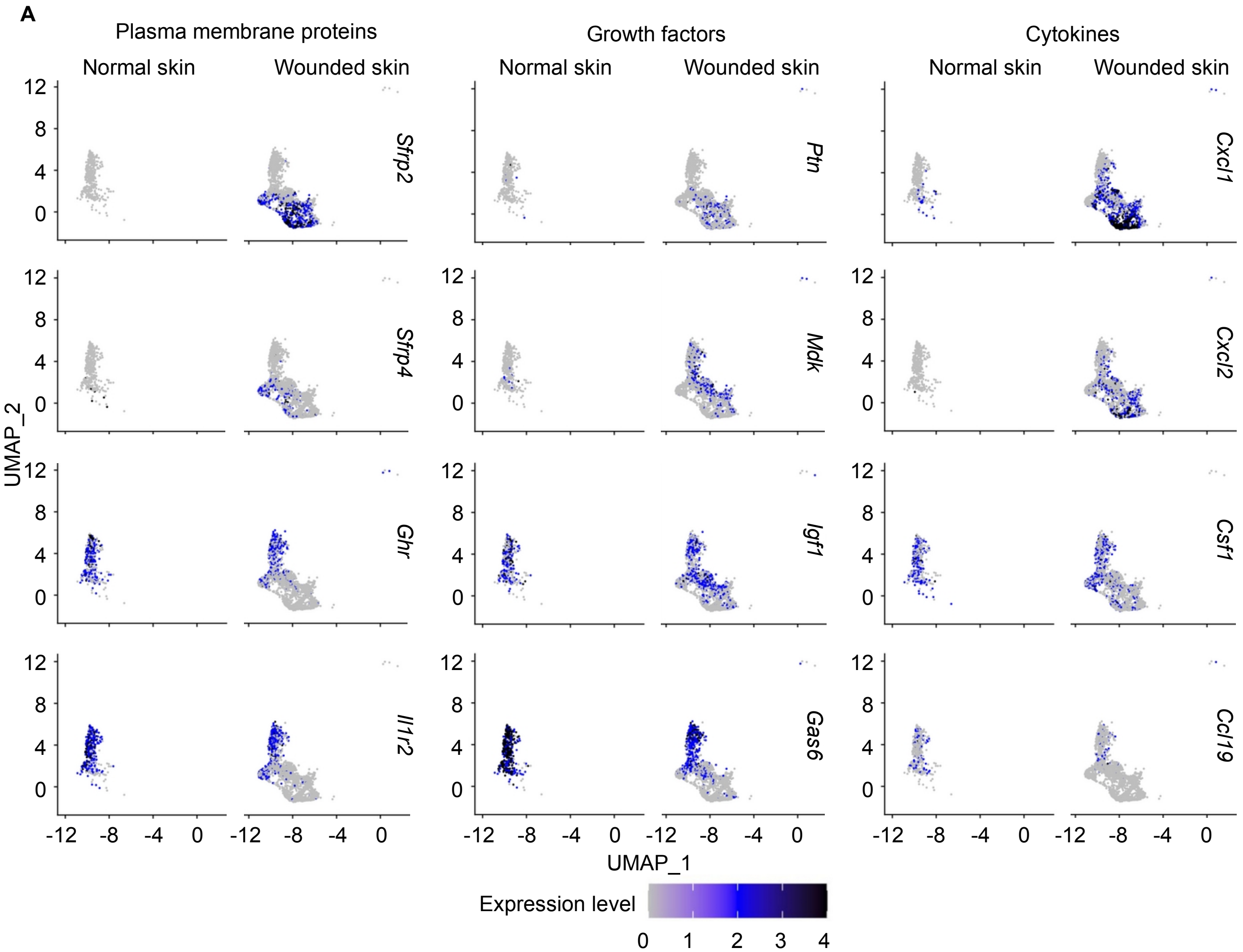

Supplementary fig 4. Cluster 3 emerges during the wound healing process in GSE142471. (A) Top upregulated and downregulated plasma membrane proteins (left panel), growth factors (middle panel) and cytokines (right panel) in cluster 3 from wounded skin samples compared to cluster three cells from normal skin.

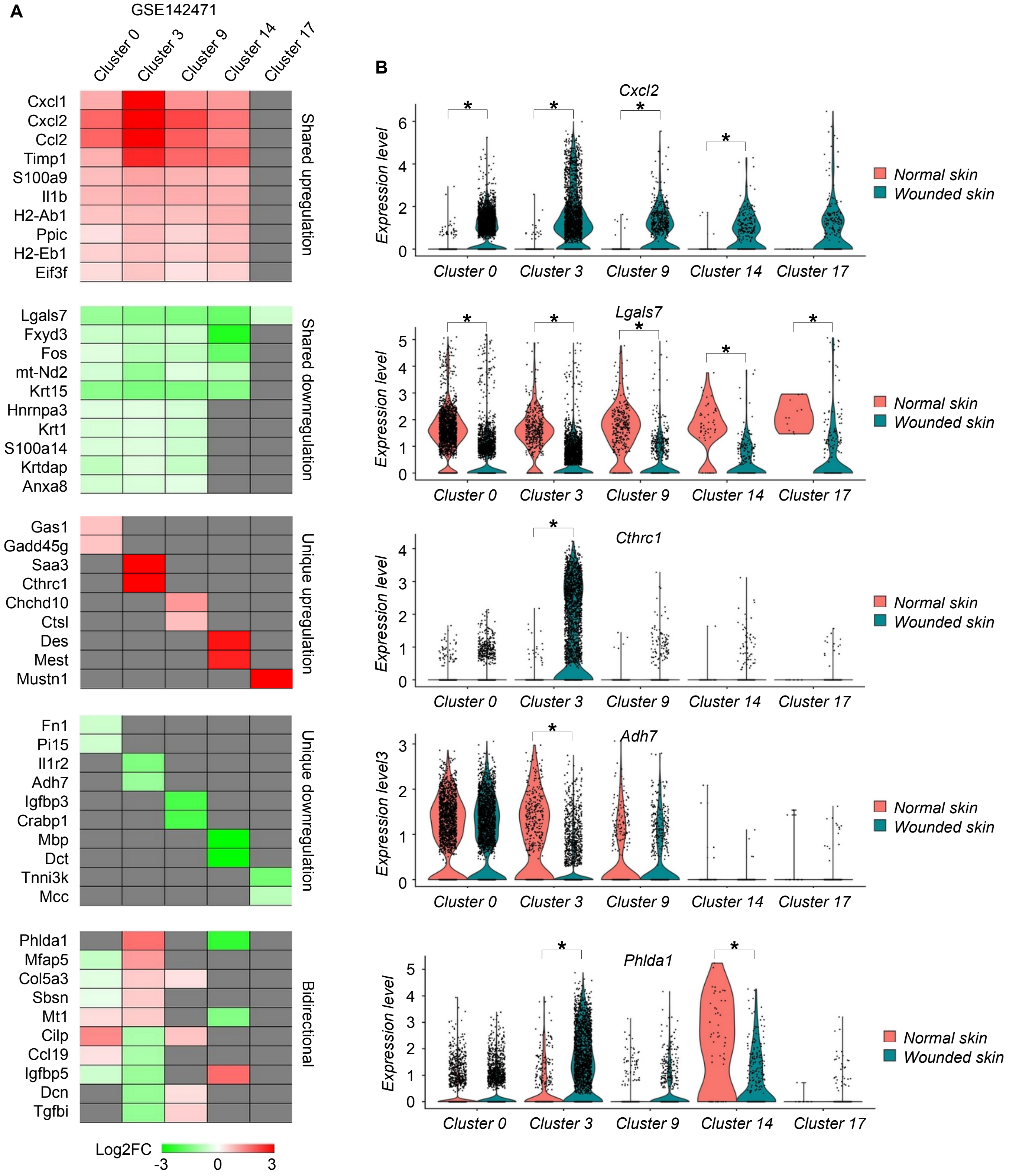

Supplementary fig 5. Differentially expressed genes in fibroblast clusters in GSE142471. (A) Heatmaps representing the log2 fold change of differentially expressed genes between wounded skin fibroblasts and normal skin fibroblasts. (B) Example genes from heatmaps in A. Stars denote adjusted p value < 0.05 and log2FC ±0.25

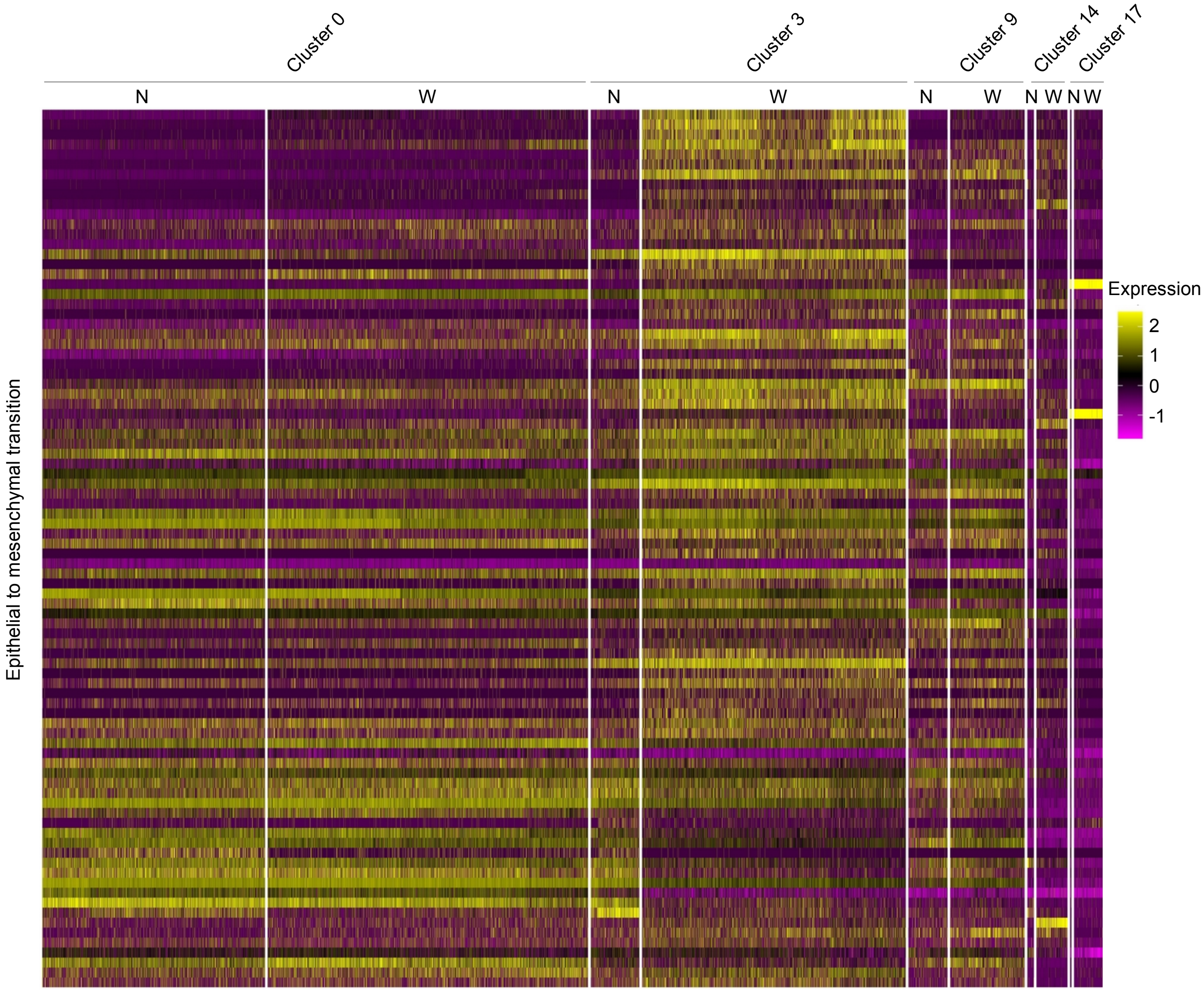

Supplementary fig 6. Differentially expressed genes involved in EMT in cluster 3 wounded skin compared to cluster 3 skin in GSE142471. Heatmap representing the expression level of differentially expressed EMT genes resulting from comparing cluster 3 cells in wounded skin with cluster 3 cells in normal skin in different clusters labeled as fibroblasts. Name and order of genes are mentioned in supplementary file 7.

Analysis of GSE178758 (Label transfer from GSE142471)

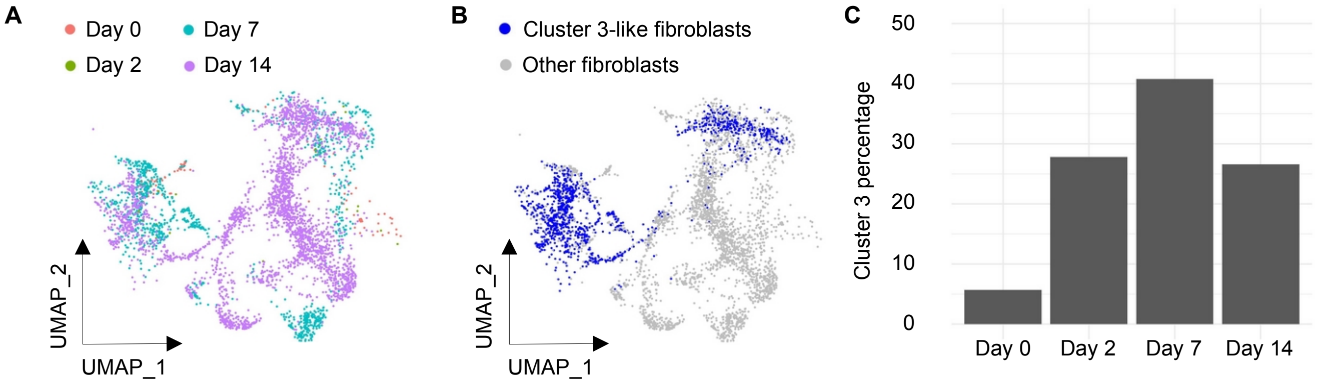

Supplementary fig 7. Cluster 3-like cells increase during wound healing. (A-B) UMAP plots representing fibroblasts from GSE178758 at day 0 (no wound), day 2, 7 and 14 post-wound. (C) Bar plot of percentage of cluster 3-like cells among fibroblasts population in GSE178758.

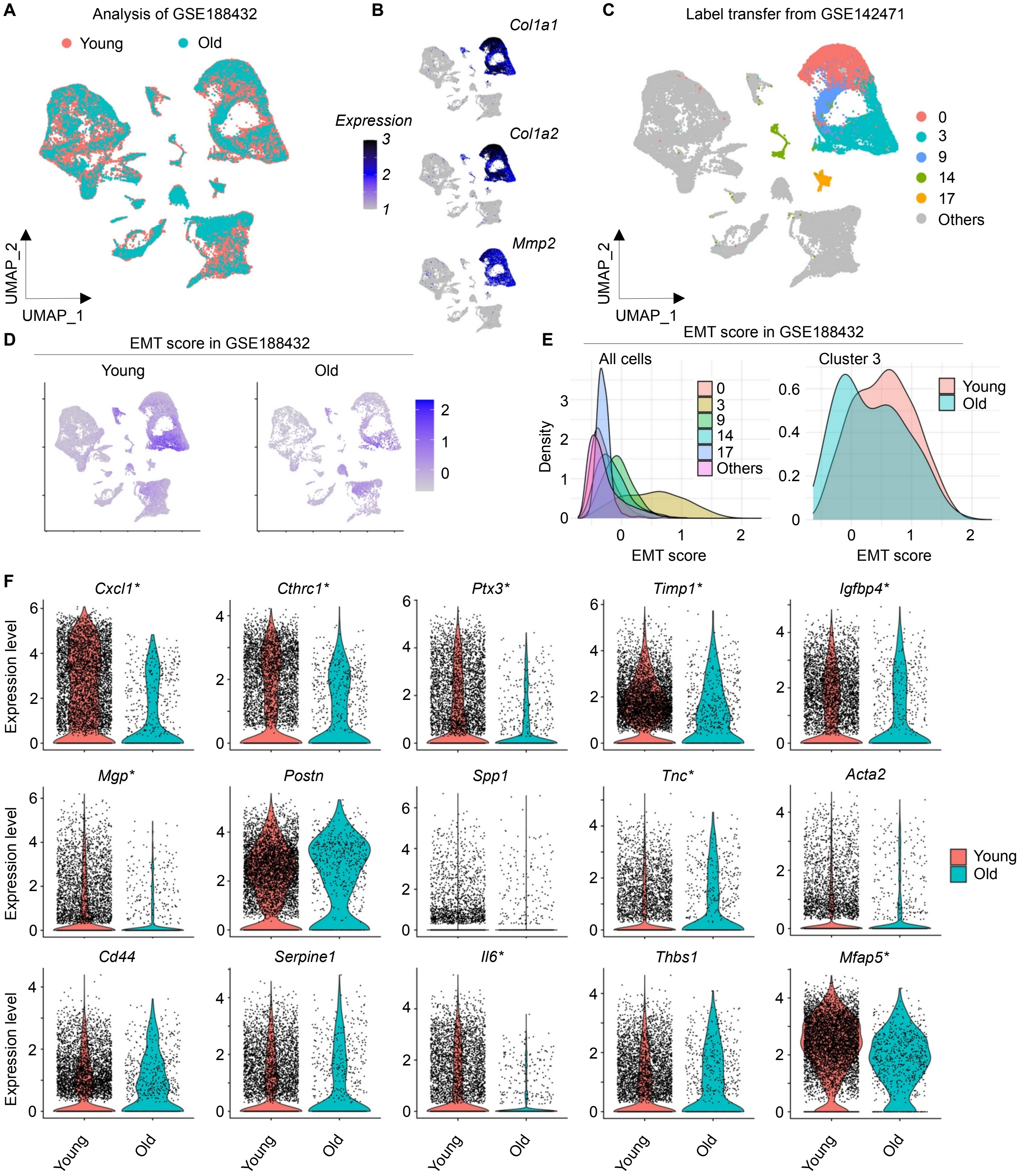

Supplementary fig 8. Analysis of wound healing in young and aged mice skin. (A) UMAP representing skin cells from 7 young and 3 aged mice from GSE188432. (B) Expression level of *Col1a1*, *Col1a2* and *Mmp2*. (C) UMAP plot representing cells from GSE188432 labeled using mutual nearest neighbor and label transfer (see methods) from GSE142471. (D) EMT score in cells from GSE188432. (E) Left panel represents EMT score in fibroblast clusters and other cell types. Right panel represents EMT score in cluster 3-like cells from GSE188432 in young and aged skin. (F) Violin plots representing the top identified 15 EMT-related genes which were used in calculating EMT score. Stars denote the genes with adjusted p value < 0.05.
