# Supplementary material for: Adult skin fibroblast state change in murine wound healing
Source: Sci Rep. 2023 Jan 17;13:886. doi: 10.1038/s41598-022-27152-4 (PMC9845335; doi:10.1038/s41598-022-27152-4)
Supplement: Supplementary file 3 — Supplementary Information 3. [file 41598_2022_27152_MOESM3_ESM.pdf]

Supplementary table 1: Metadata for single cells

Number of cells passed quality control criteria for each sample in GSE142471

Sample	GEO accession number	Excluded	Included
Normal skin 1	GSM4230076	110	5262
Normal skin 2	GSM4230077	150	5367
Wound 1	GSM4230078	80	7498
Wound 2	GSM4230079	143	4555
Wound 3	GSM4230080	111	4041

Number and percentage of cells in each cluster in each sample in GSE142471

Number of cells in each cluster

Cluster number	Normal skin 1	Normal skin 2	Wound 1	Wound 2	Wound 3
Cluster 0	400	1516	1136	1084	536
Cluster 1	1330	320	1126	25	91
Cluster 2	1032	681	834	60	149
Cluster 3	60	358	1011	613	644
Cluster 4	860	734	620	179	114
Cluster 5	97	130	406	1049	788
Cluster 6	773	511	208	21	26
Cluster 7	4	32	214	374	646
Cluster 8	144	214	102	514	240
Cluster 9	56	280	210	229	202
Cluster 10	51	43	654	27	93
Cluster 11	213	175	202	9	29
Cluster 12	113	229	78	16	19
Cluster 13	91	51	168	15	22
Cluster 14	12	44	81	47	138
Cluster 15	19	39	118	39	104
Cluster 16	0	0	73	154	80
Cluster 17	6	5	124	50	78
Cluster 18	1	5	133	50	42
Total	5262	5367	7498	4555	4041

Percentage of cells in each cluster

Cluster number	Normal skin 1	Normal skin 2	Wound 1	Wound 2	Wound 3
Cluster 0	7.6	28.25	15.15	23.8	13.26
Cluster 1	25.28	5.96	15.02	0.55	2.25
Cluster 2	19.61	12.69	11.12	1.32	3.69
Cluster 3	1.14	6.67	13.48	13.46	15.94
Cluster 4	16.34	13.68	8.27	3.93	2.82
Cluster 5	1.84	2.42	5.41	23.03	19.5
Cluster 6	14.69	9.52	2.77	0.46	0.64
Cluster 7	0.08	0.6	2.85	8.21	15.99
Cluster 8	2.74	3.99	1.36	11.28	5.94
Cluster 9	1.06	5.22	2.8	5.03	5
Cluster 10	0.97	0.8	8.72	0.59	2.3
Cluster 11	4.05	3.26	2.69	0.2	0.72
Cluster 12	2.15	4.27	1.04	0.35	0.47
Cluster 13	1.73	0.95	2.24	0.33	0.54

Cluster 14	0.23	0.82	1.08	1.03	3.41
Cluster 15	0.36	0.73	1.57	0.86	2.57
Cluster 16	0	0	0.97	3.38	1.98
Cluster 17	0.11	0.09	1.65	1.1	1.93
Cluster 18	0.02	0.09	1.77	1.1	1.04

Cell number in each group

Cluster number	Normal skin group	Wounded skin group
Cluster 0	1916	2756
Cluster 1	1650	1242
Cluster 2	1713	1043
Cluster 3	418	2268
Cluster 4	1594	913
Cluster 5	227	2243
Cluster 6	1284	255
Cluster 7	36	1234
Cluster 8	358	856
Cluster 9	336	641
Cluster 10	94	774
Cluster 11	388	240
Cluster 12	342	113
Cluster 13	142	205
Cluster 14	56	266
Cluster 15	58	261
Cluster 16	0	307
Cluster 17	11	252
Cluster 18	6	225
Total	10629	16094

Cell percentage in each group

Cluster number	Normal skin group	Wounded skin group
Cluster 0	18.03	17.12
Cluster 1	15.52	7.72
Cluster 2	16.12	6.48
Cluster 3	3.93	14.09
Cluster 4	15	5.67
Cluster 5	2.14	13.94
Cluster 6	12.08	1.58
Cluster 7	0.34	7.67
Cluster 8	3.37	5.32
Cluster 9	3.16	3.98
Cluster 10	0.88	4.81
Cluster 11	3.65	1.49
Cluster 12	3.22	0.7
Cluster 13	1.34	1.27
Cluster 14	0.53	1.65
Cluster 15	0.55	1.62
Cluster 16	0	1.91
Cluster 17	0.1	1.57

Cluster 18	0.06	1.4

Percentage of fibroblast clusters among fibroblast population in each sample

Cluster	Percentage	Sample
Cluster 0	74.91	Normal 1
Cluster 0	68.82	Normal 2
Cluster 0	44.34	Wound 1
Cluster 0	53.58	Wound 2
Cluster 0	33.54	Wound 3
Cluster 3	11.24	Normal 1
Cluster 3	16.25	Normal 2
Cluster 3	39.46	Wound 1
Cluster 3	30.3	Wound 2
Cluster 3	40.3	Wound 3
Cluster 9	10.49	Normal 1
Cluster 9	12.71	Normal 2
Cluster 9	8.2	Wound 1
Cluster 9	11.32	Wound 2
Cluster 9	12.64	Wound 3
Cluster 14	2.25	Normal 1
Cluster 14	2	Normal 2
Cluster 14	3.16	Wound 1
Cluster 14	2.32	Wound 2
Cluster 14	8.64	Wound 3
Cluster 17	1.12	Normal 1
Cluster 17	0.23	Normal 2
Cluster 17	4.84	Wound 1
Cluster 17	2.47	Wound 2
Cluster 17	4.88	Wound 3

Supplementary table 2: Cluster markers

Top 10 markers for each cluster

Gene name	p_val	avg_log2FC	p_val_adj	cluster	Type
Col1a1	0	4.25449928	0	0	other
Col1a2	0	4.069493723	0	0	other
Igfbp2	0	3.671912954	0	0	other
Sparc	0	3.611622535	0	0	other
Dcn	0	3.549348519	0	0	other
Col3a1	0	3.237646989	0	0	other
Igfbp5	0	3.068805094	0	0	other
Nupr1	0	3.017461964	0	0	transcription regulator
Lum	0	2.981454916	0	0	other
Aebp1	0	2.953695564	0	0	peptidase
Serpinb2	0	2.970751855	0	1	other
Krt14	0	2.844154726	0	1	other
Sfn	0	2.753336604	0	1	other
Dnajb1	0	2.394384581	0	1	transcription regulator
Krt5	0	2.338970762	0	1	other
Krt15	0	2.300634384	0	1	other
Avpi1	0	2.188151075	0	1	other
Fgfbp1	0	2.126452682	0	1	other
Atf3	0	2.044846821	0	1	transcription regulator
Hspb1	0	2.034944884	0	1	other
Krt10	0	4.931827824	0	2	other
Krt1	0	4.858028696	0	2	other
Krtdap	0	4.255921659	0	2	other
Mt4	0	3.754127938	0	2	other
Krt77	0	3.49959709	0	2	other
Calm4	0	3.290364745	0	2	
Fam25c	0	2.996718306	0	2	other
Dmkn	0	2.884535235	0	2	other
Ly6g6c	0	2.544207612	0	2	other
Lypd3	0	2.47498967	0	2	other
Gpx3	0	4.027033875	0	3	enzyme
Saa3	0	3.086377947	0	3	
Ptx3	0	3.063999903	0	3	other
Mfap5	0	3.012747305	0	3	other
Cthrc1	0	2.841956731	0	3	other
Cxcl1	0	2.801070478	0	3	cytokine
Ccl2	0	2.618377303	0	3	cytokine
Apod	0	2.574824124	0	3	transporter
Timp1	0	2.544132733	0	3	cytokine
Cxcl12	0	2.509728816	0	3	cytokine
Cst6	3E-257	4.638853544	7.9E-253	4	other
Defb6	0	4.341228881	0	4	
Krt79	0	4.328555125	0	4	other
Krt17	0	3.702186853	0	4	other
Sostdc1	0	2.922887578	0	4	growth factor
Aqp3	0	2.32869533	0	4	transporter

Ccl27a	0	2.130545591	0	4	
Apoe	0	1.941956575	0	4	transporter
Apoc1	0	1.875532355	0	4	transporter
Fxyd3	0	1.777049624	0	4	ion channel
Cd74	0	4.856290966	0	5	transmembrane receptor
H2-Aa	0	4.70730122	0	5	
H2-Eb1	0	4.597335749	0	5	
H2-Ab1	0	4.596750686	0	5	
Il1b	0	3.636053568	0	5	cytokine
Lyz2	0	3.373798848	0	5	
Ccr7	0	3.254825894	0	5	G-protein coupled receptor
Cd83	0	3.171593251	0	5	transmembrane receptor
Ifitm1	0	3.110070194	0	5	transmembrane receptor
H2-DMa	0	2.942551522	0	5	
Fcgbp	0	2.118885147	0	6	other
Col17a1	0	2.043670997	0	6	other
Ccl27a	0	1.767847829	0	6	
Krt5	0	1.67346266	0	6	other
Trp63	0	1.576853404	0	6	
Cxcl14	0	1.536746406	0	6	cytokine
Krt14	0	1.462188097	0	6	other
Krt15	0	1.420784191	0	6	other
Fxyd3	0	1.384936154	0	6	ion channel
4631405K08Rik	0	1.377511425	0	6	
Pf4	0	5.087411171	0	7	cytokine
C1qb	0	4.350577522	0	7	other
C1qa	0	4.087719061	0	7	other
Cxcl2	0	3.955854162	0	7	cytokine
C1qc	0	3.557403361	0	7	other
Lyz2	0	3.400145101	0	7	
Cxcl3	0	3.322992715	0	7	cytokine
Ccl8	3.5E-13	3.270938159	9.816E-09	7	cytokine
Ms4a7	0	3.238768975	0	7	other
Tnf	0	3.08688712	0	7	cytokine
Cd3g	0	4.833941095	0	8	transmembrane receptor
Ctla2a	0	4.261114286	0	8	
Nkg7	0	4.147828636	0	8	other
Xcl1	0	4.053022864	0	8	cytokine
Cd7	0	3.677243402	0	8	other
Cd3e	0	3.650638057	0	8	transmembrane receptor
Cd3d	0	3.273680304	0	8	transmembrane receptor
Ptprcap	0	3.219206004	0	8	other
Rgs2	0	3.106182513	0	8	enzyme
Ctsw	0	3.050683873	0	8	peptidase
Coch	0	3.858805078	0	9	other
Crabp1	9E-267	3.448314274	2.38E-262	9	transporter
Mgp	4E-106	2.375131658	1.16E-101	9	other
Dkk2	0	2.368067951	0	9	other
Gas1	0	2.241527424	0	9	other

Mfap4	5E-215	2.150477316	1.37E-210	9	other
Col6a3	0	1.946471775	0	9	other
Enpp2	0	1.937161695	0	9	enzyme
Cilp	0	1.912270068	0	9	phosphatase
Bgn	0	1.881649464	0	9	other
Acta2	0	5.990082773	0	10	other
Rgs5	0	5.375130373	0	10	enzyme
Tagln	0	4.982452536	0	10	other
Myl9	0	4.899326615	0	10	other
Gm13889	0	4.659396639	0	10	
Fabp4	0	4.072014178	0	10	transporter
Rgs16	0	3.924756248	0	10	enzyme
Sparcl1	0	3.707111761	0	10	other
Serpine2	0	3.590859376	0	10	other
Tinagl1	0	3.354955672	0	10	transporter
Stmn1	0	3.228117683	0	11	other
Hmgb2	0	2.802331536	0	11	transcription regulator
Ube2c	0	2.685008458	0	11	enzyme
Cdc20	0	2.531347201	0	11	other
Cks2	0	2.498984045	0	11	kinase
Cenpa	0	2.3477844	0	11	other
Tubb4b	3E-203	2.217997347	9.68E-199	11	other
H2afv	0	2.151022531	0	11	other
Cdca8	0	2.127067549	0	11	other
Ube2s	2E-248	2.088652976	6.75E-244	11	enzyme
Ptn	0	4.149694644	0	12	growth factor
Krt17	4E-184	3.723570951	1.07E-179	12	other
Calml3	0	3.665630691	0	12	other
Cxcl14	4E-255	3.292817225	1E-250	12	cytokine
Tgm5	0	2.867689399	0	12	enzyme
Aqp3	0	2.839109842	0	12	transporter
Nrtn	0	2.691408159	0	12	growth factor
Postn	0	2.623732126	0	12	other
Wfdc3	0	2.482397584	0	12	other
Fst	7E-217	2.326787119	1.9E-212	12	other
Akr1c18	0	5.209101355	0	13	
Scd1	2E-268	3.3727004	5.64E-264	13	
Plin2	5E-248	3.062727724	1.35E-243	13	other
Psph	0	2.980496007	0	13	phosphatase
Apoc1	8E-251	2.895750774	2.24E-246	13	transporter
Ldhb	0	2.718711121	0	13	enzyme
Mgst1	1E-214	2.709182641	3E-210	13	enzyme
Acsbg1	0	2.551153568	0	13	enzyme
Fam213a	0	2.329453977	0	13	
Cers4	2E-251	2.233911959	4.22E-247	13	transcription regulator
Pmp22	5.3E-17	2.600354173	1.492E-12	14	other
Cdkn1c	2.7E-43	2.480410166	7.496E-39	14	other
Gatm	2E-276	2.447308771	4.77E-272	14	enzyme
Plp1	0	2.444356492	0	14	other

Mest	0	2.274061813	0	14	peptidase
Pdlim4	4E-187	2.256309294	1.07E-182	14	other
Cnn3	4E-216	2.160020381	1.14E-211	14	other
Cryab	2.7E-95	2.117880208	7.565E-91	14	other
Tubb2b	0	2.114867623	0	14	other
Itm2a	1E-151	2.109731801	2.88E-147	14	other
Ccl21a	4E-102	6.65543888	1.178E-97	15	
Fabp4	0	4.929704068	0	15	transporter
Aqp1	0	3.897908137	0	15	transporter
Plvap	0	3.87149309	0	15	other
Cldn5	0	3.860771145	0	15	other
Lrg1	0	3.757833836	0	15	other
Tm4sf1	0	3.668846573	0	15	other
Egfl7	0	3.595234785	0	15	other
Rnd1	0	3.15939807	0	15	enzyme
8430408G22Rik	0	3.073202992	0	15	
S100a9	3E-203	7.084648573	9.59E-199	16	other
S100a8	3E-234	6.777885513	9.12E-230	16	other
Ccl3	4E-303	6.184063802	1.15E-298	16	cytokine
Hdc	0	5.230779363	0	16	enzyme
Irg1	0	5.132234794	0	16	
Ccl6	0	4.850247142	0	16	
G0s2	0	4.815399384	0	16	other
Slpi	0	4.75387556	0	16	other
Ccl4	1E-146	4.740485899	4E-142	16	cytokine
Cxcl2	9E-171	4.528503221	2.43E-166	16	cytokine
Acta1	0	9.155066989	0	17	other
Mylpf	0	8.043875316	0	17	other
Tnnc2	0	7.813381137	0	17	other
Myl1	0	7.693164315	0	17	other
Tnnt3	0	7.132902514	0	17	other
Ckm	0	6.969819515	0	17	kinase
Tnni2	0	6.912732478	0	17	enzyme
Pvalb	0	6.448863357	0	17	other
Tpm1	2E-202	5.79137171	4.94E-198	17	other
Des	0	5.645229618	0	17	other
Cd207	0	5.344911876	0	18	other
H2-M2	0	3.980595889	0	18	
Mfge8	2E-105	3.949242251	4.32E-101	18	other
Rgs2	1E-109	3.552528095	4.2E-105	18	enzyme
Rgs1	1E-116	3.492146576	2.84E-112	18	enzyme
H2-Ab1	2.4E-96	3.299298074	6.615E-92	18	
Cd74	6.2E-91	3.263405436	1.733E-86	18	transmembrane receptor
Grasp	1E-177	3.016272807	3.56E-173	18	other
Pxdc1	2E-107	2.923189821	4.36E-103	18	other
H2-Eb1	1.8E-97	2.916812023	5.153E-93	18	

Supplementary table 3: DEGs between all wounded cells compared to all normal cells

DEGs with log2FC ±0.5 (GSE142471)

Highlighted in red: upregulated genes

Highlighted in green: downregulated genes

Gene name	p_val	avg_log2FC	p_val_adj	Type
Cxcl2	0	4.790879315	0	cytokine
Ccl4	0	3.388425356	0	cytokine
Il1b	0	3.293369409	0	cytokine
Ccl3	0	3.196460897	0	cytokine
Cxcl1	0	2.950699853	0	cytokine
Acta1	0	2.939692961	0	other
S100a9	0	2.820375937	0	other
Ccl2	0	2.580708298	0	cytokine
S100a8	0	2.521092886	0	other
Srgn	0	2.347717833	0	other
Slpi	0	2.327870229	0	other
Tyrobp	0	2.291617225	0	transmembrane receptor
Ifi27l2a	0	2.263964276	0	other
Plac8	0	2.253947542	0	other
H2-Aa	0	2.210331273	0	transmembrane receptor
Lyz2	0	2.20782292	0	enzyme
Ifitm1	0	2.190654681	0	transmembrane receptor
Ctss	0	1.97814538	0	peptidase
H2-Ab1	0	1.962413979	0	other
H2-Eb1	0	1.948869328	0	transmembrane receptor
Cd74	0	1.931569905	0	transmembrane receptor
Cd14	0	1.905942983	0	transmembrane receptor
Clec4e	0	1.818421687	0	other
Hilpda	0	1.736159569	0	other
Acta2	0	1.699777134	0	other
Apod	3E-249	1.669887518	9.36E-245	transporter
Thbs1	0	1.648011761	0	other
Fcer1g	0	1.642506649	0	transmembrane receptor
Ptgs2	0	1.616612444	0	enzyme
Ccl7	0	1.607754145	0	cytokine
Cd52	0	1.594695578	0	other
Ftl1	0	1.591658503	0	
Ccl9	0	1.559413181	0	
Cyba	0	1.542915105	0	enzyme
Gpx3	0	1.521909784	0	enzyme
Timp1	0	1.506643729	0	cytokine
Bcl2a1b	0	1.500309249	0	
Marcksl1	0	1.490716812	0	other
Ifrd1	0	1.478351654	0	other
Slfn2	0	1.464568802	0	
Fabp5	1E-141	1.419477236	3.76E-137	transporter
Fth1	0	1.413618248	0	enzyme
Isg15	0	1.412728058	0	other
Wfdc17	0	1.398553206	0	

Il1rn	2E-159	1.375052105	5.1E-155	cytokine
Pim1	0	1.367430087	0	kinase
Lgmn	0	1.353939202	0	peptidase
Tpm1	2.7E-85	1.306919386	7.464E-81	other
Lilr4b	0	1.297692188	0	
Ccrl2	0	1.281366418	0	G-protein coupled receptor
Tpm2	5E-150	1.268520148	1.36E-145	other
Tnfaip6	0	1.264430215	0	other
Alox5ap	0	1.263918747	0	other
Plek	0	1.250169565	0	other
Coro1a	0	1.24442669	0	other
Il6	3E-264	1.233767307	7.2E-260	cytokine
Ctsz	0	1.221479018	0	peptidase
Lsp1	0	1.211472112	0	other
Fxyd5	0	1.175696079	0	ion channel
Cd83	0	1.172405687	0	transmembrane receptor
Traf1	0	1.153210377	0	other
Basp1	0	1.152076176	0	transcription regulator
Hmox1	0	1.141697554	0	enzyme
Phlda1	0	1.123936812	0	other
Igfbp7	7E-194	1.123875889	2.08E-189	transporter
Vim	0	1.120544017	0	other
Lgals1	0	1.113831996	0	other
Lilrb4a	0	1.108158593	0	
Ccl8	1E-291	1.091098223	3.73E-287	cytokine
Ctsk	1E-185	1.067950737	3.38E-181	peptidase
Sqstm1	0	1.042798773	0	transcription regulator
Plin2	0	1.041497048	0	other
Bst2	0	1.035938657	0	other
Tnfaip2	0	1.030607889	0	other
Ifi30	5E-195	1.007422934	1.37E-190	enzyme
Bgn	5E-264	0.996329382	1.38E-259	other
Laptm5	0	0.979146751	0	other
Gapdh	0	0.977550399	0	enzyme
Calm1	0	0.962987566	0	other
Mxd1	0	0.961676273	0	transcription regulator
Arpc1b	0	0.960420138	0	other
Ninj1	1E-216	0.952909171	3.12E-212	other
Cd53	0	0.952902083	0	other
Cpxm1	2E-256	0.942519955	4.77E-252	peptidase
Tmsb4x	6E-222	0.918154639	1.65E-217	other
Col6a3	1E-273	0.913680683	3.02E-269	other
Nfkbia	0	0.912621579	0	transcription regulator
Lst1	0	0.897696984	0	other
Spi1	0	0.896795113	0	transcription regulator
Ptpn18	0	0.886219509	0	phosphatase
Cytip	0	0.885243356	0	other
Cd68	0	0.879497537	0	other
Tmem176a	0	0.876429335	0	other

Mfge8	3E-266	0.869228037	8.19E-262	other
Cxcl16	3E-149	0.862353475	9.02E-145	cytokine
Tmem176b	0	0.85727065	0	other
Ier5	0	0.855943501	0	other
Cxcl12	6E-176	0.851492558	1.74E-171	cytokine
Ehd1	0	0.851465188	0	other
Gadd45b	0	0.850067618	0	other
Cd44	0	0.849205666	0	other
Plaur	0	0.835974133	0	transmembrane receptor
Postn	2E-164	0.824376367	4.69E-160	other
Serpina3n	1E-105	0.823387054	3.46E-101	
Csrp2	1E-298	0.822138969	3.99E-294	other
Ifitm3	0	0.821157016	0	other
Npc2	0	0.810829023	0	transporter
Igfbp4	5E-200	0.810685651	1.52E-195	other
Id2	4E-229	0.810251276	1.18E-224	transcription regulator
Serpine1	3E-236	0.809053421	9.54E-232	other
Gadd45g	5E-146	0.806602709	1.35E-141	other
Fstl1	2E-215	0.804125361	4.22E-211	other
Mfap5	6.6E-73	0.80149199	1.843E-68	other
H2-DMa	5E-251	0.797371378	1.48E-246	
Sub1	0	0.784827614	0	transcription regulator
Gngt2	0	0.784403952	0	enzyme
Rgs1	0	0.77642224	0	enzyme
Odc1	4E-146	0.774851181	1.03E-141	enzyme
Ly6e	0	0.766551318	0	other
Cilp	3E-150	0.766475506	8.06E-146	phosphatase
Lmnb1	0	0.754719074	0	other
Lcp1	0	0.750688416	0	other
Tnfaip3	0	0.746470149	0	enzyme
Gmfg	0	0.745909299	0	growth factor
Actb	4E-280	0.745137269	1.15E-275	other
AW112010	3E-141	0.744851805	8.35E-137	
Rilpl2	0	0.743911459	0	other
Rac2	0	0.740767065	0	enzyme
Ier3	4E-201	0.737101801	9.95E-197	other
H2-DMb1	6E-141	0.736776686	1.76E-136	
Ctsb	4E-218	0.729675291	1.11E-213	peptidase
Mfap4	7.8E-79	0.727584182	2.189E-74	other
Zeb2	0	0.725240262	0	transcription regulator
Mmp3	2E-105	0.722653626	5.45E-101	peptidase
Cstb	4E-167	0.721105556	1.12E-162	peptidase
Ifi205	1E-303	0.714054101	3.99E-299	
Ccl11	3E-112	0.710856915	8.79E-108	cytokine
Dpt	1E-144	0.70995943	3.63E-140	other
Col6a2	2E-202	0.709281789	6.73E-198	other
C3	2E-158	0.706395314	6.82E-154	peptidase
Efhd2	0	0.704364987	0	other
Dusp5	0	0.70291039	0	phosphatase

Col6a1	6E-193	0.692876378	1.63E-188	other
Tnfrsf1b	0	0.685008897	0	transmembrane receptor
Gng11	3E-239	0.68165597	7.66E-235	other
Psap	1E-237	0.680979598	3.36E-233	enzyme
Sdcbp	0	0.680290511	0	enzyme
Rhoc	0	0.673881977	0	enzyme
Ifitm2	7E-303	0.665487453	1.95E-298	other
Rnf149	2E-110	0.665402658	5.79E-106	enzyme
Tmsb10	0	0.659224008	0	other
Kdm6b	2E-237	0.65552012	6.97E-233	enzyme
Sod2	7E-119	0.654074506	1.87E-114	enzyme
Dpep1	3E-187	0.651358373	8.85E-183	peptidase
Htra3	7E-141	0.647062373	1.87E-136	peptidase
Lpl	1E-221	0.63853617	3.59E-217	enzyme
Marcks	8E-291	0.63651344	2.28E-286	other
Ctsc	4E-213	0.634087538	1.25E-208	peptidase
Crip1	0	0.633773519	0	other
Sod3	2E-232	0.633763473	4.47E-228	enzyme
Itm2a	5E-164	0.616661373	1.3E-159	other
Card19	8E-173	0.614491222	2.24E-168	other
Btg1	9E-160	0.613550047	2.54E-155	transcription regulator
Ctsl	4E-156	0.612731774	1.15E-151	peptidase
Mcl1	4E-220	0.611857969	1.16E-215	transporter
Col4a1	3E-162	0.611441071	9.01E-158	other
Cald1	6E-182	0.604385786	1.65E-177	other
Hexa	0	0.603693632	0	enzyme
Eln	9.8E-53	0.600903694	2.743E-48	other
Txn1	9E-265	0.598775619	2.58E-260	
Serping1	2E-128	0.598276479	5.08E-124	other
Map4k4	6E-245	0.595379183	1.66E-240	kinase
Lgals3bp	0	0.592992882	0	transmembrane receptor
Ddit4	4E-151	0.592414246	1.17E-146	other
Grn	3E-269	0.589685674	7.74E-265	growth factor
C1s1	1E-253	0.589563119	3.5E-249	
Ifi204	0	0.589514065	0	
H2afz	5E-121	0.589479109	1.49E-116	other
Ptp4a1	2E-289	0.587135293	4.67E-285	phosphatase
Rel	3E-167	0.586984577	7.15E-163	transcription regulator
Cryab	9.6E-39	0.586755096	2.675E-34	other
Aspn	9E-153	0.584440707	2.53E-148	other
Arpc3	0	0.5827131	0	other
Clic4	3E-305	0.573705911	7.25E-301	ion channel
Slc25a4	1.2E-99	0.568481686	3.415E-95	transporter
Gm12840	5.7E-74	0.568138579	1.604E-69	
Nid1	3E-274	0.566147121	7.1E-270	other
Sparcl1	1.9E-80	0.564665948	5.182E-76	other
Syngr2	2.3E-74	0.564094356	6.316E-70	other
Ndrg1	2E-157	0.561283785	5.98E-153	kinase
Irf7	0	0.559374995	0	transcription regulator

Cflar	5E-236	0.558536294	1.37E-231	other
Sh3bgrl3	8.4E-90	0.557431119	2.339E-85	other
Unc93b1	0	0.554846127	0	other
Pmaip1	2E-140	0.553127812	5.88E-136	other
Smox	2E-196	0.552349297	4.43E-192	enzyme
Col4a2	1E-163	0.552175322	3.79E-159	other
Sepp1	1.5E-94	0.551445394	4.325E-90	
Gm2a	1E-236	0.549922461	2.82E-232	enzyme
Cox17	2E-241	0.549869378	6.86E-237	enzyme
Fam49b	5E-219	0.548641983	1.46E-214	other
Cotl1	6E-116	0.53962438	1.76E-111	other
Col5a2	4E-160	0.539508448	1.08E-155	other
Cd302	3E-238	0.539271601	8.78E-234	transmembrane receptor
Pfn1	3E-217	0.535242163	9.04E-213	other
Cfl1	3E-263	0.534631275	8.31E-259	other
Ldha	2E-133	0.533327934	4.26E-129	enzyme
Picalm	1E-289	0.523839807	3.13E-285	other
Pi16	2.9E-59	0.520382234	8.062E-55	other
Errfi1	2E-141	0.519129444	6.75E-137	other
Pdpn	2E-151	0.518391259	6.09E-147	other
Birc3	5E-304	0.518153377	1.5E-299	enzyme
2010107E0	0	0.515658592	0	
Akap13	2E-292	0.51471721	5.58E-288	other
Ppic	1.6E-90	0.511307401	4.417E-86	enzyme
Tubb6	8E-305	0.510392244	2.13E-300	other
Cebpb	2.9E-48	0.508471169	8.058E-44	transcription regulator
Anxa3	7E-200	0.508447947	1.83E-195	enzyme
Mndal	0	0.508071622	0	
Ifi203	0	0.507912447	0	
Gpnmb	2E-143	0.506861734	6.6E-139	enzyme
Mxra8	6E-288	0.505632392	1.63E-283	other
Has1	2.7E-86	0.504239855	7.627E-82	enzyme
Fgl2	8E-214	0.503165148	2.12E-209	peptidase
Angptl4	5E-250	0.502898448	1.26E-245	other
Limd2	5E-307	0.502365834	1.49E-302	other
Ppp1r15a	3E-218	0.500265552	7.87E-214	other
Tnfrsf18	0	-0.50322696	0	transmembrane receptor
Ptprf	0	-0.50506639	0	phosphatase
Gstp1	8E-250	-0.50649824	2.33E-245	enzyme
Ikzf2	0	-0.50910109	0	transcription regulator
Mif	0	-0.51420069	0	cytokine
Rsrp1	4E-241	-0.5150335	1.13E-236	other
Epcam	5E-298	-0.51675059	1.35E-293	other
Fgfbp1	0	-0.51733709	0	other
Sema3c	3E-280	-0.5215213	8.68E-276	other
Il34	2E-297	-0.52313511	6.2E-293	other
Fam132a	2E-110	-0.52708779	4.31E-106	
Gpha2	0	-0.53015464	0	other
Sptssa	1E-292	-0.53172097	2.86E-288	enzyme

Rpl13-ps3	0	-0.53292361	0	
Neat1	0	-0.53429784	0	other
Pkp1	0	-0.53739347	0	other
Kif21a	0	-0.53813207	0	other
Cav2	0	-0.53977673	0	other
Ppp1r14b	0	-0.5461878	0	phosphatase
Ivns1abp	0	-0.55178989	0	other
Dynlt3	0	-0.56142347	0	other
Dsg1a	0	-0.56384541	0	
Rab3d	0	-0.56407915	0	enzyme
Lamb3	0	-0.56609996	0	transporter
Gm13305	0	-0.56735977	0	
Hoxa7	0	-0.57084892	0	transcription regulator
Srsf2	0	-0.57179107	0	transcription regulator
Mgst3	0	-0.5722843	0	enzyme
Nfib	4E-279	-0.57268653	9.96E-275	transcription regulator
Klc3	0	-0.58240898	0	other
Xist	0	-0.58245019	0	other
Gm42418	2E-155	-0.58675931	4.47E-151	
Rarg	0	-0.59043608	0	ligand-dependent nuclear receptor
Serpinb5	0	-0.5918432	0	other
Btg2	3.3E-76	-0.59387419	9.224E-72	transcription regulator
Atf3	3E-119	-0.59494838	9.58E-115	transcription regulator
Bbox1	0	-0.5950746	0	enzyme
Efna3	0	-0.59680892	0	kinase
Dst	0	-0.59804668	0	other
Fos	1E-202	-0.60491547	3.99E-198	transcription regulator
Cirbp	0	-0.60559695	0	translation regulator
Ptma	0	-0.61884945	0	other
Oas1f	0	-0.62246122	0	
Itga6	0	-0.62337248	0	transmembrane receptor
S100a16	0	-0.6236249	0	other
Aqp3	1E-129	-0.62519549	3.37E-125	transporter
Rpl6l	0	-0.62582354	0	
Gm16136	0	-0.62678195	0	
Gas6	0	-0.62818853	0	growth factor
Pdzk1ip1	0	-0.62825538	0	other
Sdc4	0	-0.63174754	0	other
Il20rb	0	-0.64419388	0	other
mt-Nd2	0	-0.64453414	0	
Net1	0	-0.6468814	0	other
Jup	0	-0.64901294	0	other
Sytl1	0	-0.65017579	0	enzyme
Urah	0	-0.65212671	0	
Bcam	0	-0.65586563	0	transmembrane receptor
Tecr	0	-0.66191901	0	enzyme
Krt79	1E-210	-0.68212985	2.68E-206	other
Tubb4b	8E-238	-0.68227015	2.21E-233	other
Rps18-ps3	7E-305	-0.68515408	1.94E-300	

Tacstd2	0	-0.70356334	0	other
Igfbp3	2E-192	-0.71530713	4.37E-188	other
Pou3f1	0	-0.7342576	0	transcription regulator
Rpl10-ps3	0	-0.73763524	0	
Rpl15	0	-0.74296487	0	other
4631405K08	0	-0.7472898	0	
Hs3st6	0	-0.7590391	0	enzyme
Dsc3	0	-0.76146089	0	other
Hmgn1	0	-0.76343449	0	transcription regulator
Sfn	0	-0.77048554	0	other
Ly6g6c	0	-0.77889473	0	other
Hras	0	-0.78240306	0	enzyme
Gltp	6E-264	-0.79882292	1.6E-259	transporter
Pkp3	0	-0.80889695	0	other
Gm9493	0	-0.809697	0	
Sdc1	0	-0.8154838	0	enzyme
Calm4	0	-0.82310693	0	
Gm10020	0	-0.8319109	0	
Dgcr6	0	-0.83596066	0	other
Krt14	0	-0.85495463	0	other
Ahnak2	0	-0.85521734	0	other
Hspb1	0	-0.85586284	0	other
Cd9	0	-0.86418028	0	other
Rpl9-ps6	0	-0.867244	0	
Uba52	0	-0.874306	0	enzyme
Ckmt1	0	-0.89039957	0	
Trim29	0	-0.89398686	0	transcription regulator
Emp2	0	-0.90132693	0	other
Rab25	0	-0.90212732	0	enzyme
Krt1	0	-0.91553441	0	other
Apoe	0	-0.92503072	0	transporter
Gm8730	0	-0.92697523	0	
Spint2	0	-0.93975453	0	other
Sbsn	0	-0.95509547	0	other
Mt4	0	-0.95913276	0	other
Krt77	0	-0.9643107	0	other
Dsp	0	-0.96991657	0	other
Krt17	0	-0.97260908	0	other
Trp63	0	-0.98330049	0	transcription regulator
Gata3	0	-0.98584801	0	transcription regulator
Capns2	0	-0.98842951	0	peptidase
Calm5	0	-0.99848263	0	other
Ptn	1E-210	-1.00766277	2.79E-206	growth factor
Dapl1	0	-1.04660882	0	other
Apoc1	0	-1.06066982	0	transporter
Sostdc1	0	-1.06255212	0	growth factor
Tmem45a	0	-1.0924374	0	other
Krt10	0	-1.10387166	0	other
Perp	0	-1.11407367	0	other

S100a14	0	-1.13118565	0	other
Krtdap	0	-1.13989318	0	other
Clca3a2	0	-1.15283769	0	ion channel
Fcgbp	0	-1.16233893	0	other
Fam25c	0	-1.17214963	0	other
Dmkn	0	-1.29253426	0	other
Col17a1	0	-1.29845233	0	other
Lgals7	0	-1.31421636	0	other
Fxyd3	0	-1.35110054	0	ion channel
Ly6d	0	-1.35981261	0	other
Krt5	0	-1.3620304	0	other
Ccl27a	0	-1.36379678	0	other
Cxcl14	0	-1.39573031	0	cytokine
Anxa8	0	-1.4753715	0	other
Krt15	0	-1.72416833	0	other

Supplementary table 4: **GSEA resulting from comparing all wounded cells with all normal cells**

Top upregulated gene sets (FDR 1-val < 0.001)

NAME	ES	NES	NOM p-val	FDR q-val
HALLMARK_TNFA_SIGNALING_VIA_NFKB	0.69928056	2.4741397	0	0
HALLMARK_INTERFERON_GAMMA_RESPONSE	0.7184096	2.369059	0	0
HALLMARK_INFLAMMATORY_RESPONSE	0.7467706	2.362794	0	0
HALLMARK_COMPLEMENT	0.7192742	2.354042	0	0
HALLMARK_EPITHELIAL_MESENCHYMAL_TRANSITION	0.67414165	2.3361747	0	0
HALLMARK_KRAS_SIGNALING_UP	0.74948627	2.2715156	0	0
HALLMARK_INTERFERON_ALPHA_RESPONSE	0.7240555	2.0744925	0	0.0001746
HALLMARK_ALLOGRAFT_REJECTION	0.6777527	2.0607054	0	0.0001528
HALLMARK_IL6_JAK_STAT3_SIGNALING	0.7029623	2.0098383	0	0.0002469
HALLMARK_APOPTOSIS	0.60159403	1.9645588	0	0.0005527
GOBP_GRANULOCYTE_MIGRATION	0.806583	2.3769736	0	0
GOBP_GRANULOCYTE_CHEMOTAXIS	0.8238724	2.374141	0	0
GOBP_MYELOID_LEUKOCYTE_MIGRATION	0.77410024	2.3731787	0	0
GOBP_NEUTROPHIL_CHEMOTAXIS	0.892141	2.3721952	0	0
GOBP_NEUTROPHIL_MIGRATION	0.8710328	2.3575685	0	0
GOBP_DEFENSE_RESPONSE	0.6083921	2.3451936	0	0
GOBP_LEUKOCYTE_CHEMOTAXIS	0.74766606	2.3414178	0	0
GOBP_INFLAMMATORY_RESPONSE	0.6422395	2.3269067	0	0
GOBP_RESPONSE_TO_BACTERIUM	0.65214336	2.290609	0	0
GOBP_CELLULAR_RESPONSE_TO_MOLECULE_OF_BACTERIAL_	0.73969036	2.2741787	0	0
GOBP_CELL_CHEMOTAXIS	0.68367285	2.2477105	0	0
GOBP_RESPONSE_TO_LIPID	0.59073985	2.2400901	0	0
GOBP_RESPONSE_TO_INTERFERON_GAMMA	0.7065001	2.2291975	0	0
GOBP_CYTOKINE_MEDIATED_SIGNALING_PATHWAY	0.59201586	2.224039	0	0
GOBP_RESPONSE_TO_MOLECULE_OF_BACTERIAL_ORIGIN	0.6596199	2.203845	0	0
GOBP_POSITIVE_REGULATION_OF_INFLAMMATORY_RESPONS	0.7549869	2.203779	0	0
GOBP_MAINTENANCE_OF_LOCATION	0.6539865	2.1892307	0	0
GOBP_IMMUNE_EFFECTOR_PROCESS	0.564166	2.1832232	0	0
GOBP_RESPONSE_TO_CHEMOKINE	0.88542545	2.1830068	0	0
GOBP_CELLULAR_RESPONSE_TO_BIOTIC_STIMULUS	0.70654964	2.1769311	0	0
GOBP_CYTOKINE_PRODUCTION	0.5917665	2.1690578	0	0
GOBP_INNATE_IMMUNE_RESPONSE	0.5787569	2.153816	0	4.815E-05
GOBP_POSITIVE_REGULATION_OF_DEFENSE_RESPONSE	0.6401957	2.1404321	0	7.919E-05
GOBP_G_PROTEIN_COUPLED_RECEPTOR_SIGNALING_PATHW	0.6497912	2.1407568	0	8.224E-05
GOBP_DEFENSE_RESPONSE_TO_OTHER_ORGANISM	0.574595	2.1432717	0	8.553E-05
GOBP_PHAGOCYTOSIS	0.6479781	2.1497462	0	8.909E-05
GOBP_RESPONSE_TO_BIOTIC_STIMULUS	0.5577225	2.150967	0	9.297E-05
GOBP_HUMORAL_IMMUNE_RESPONSE	0.7169174	2.1193755	0	0.0001141
GOBP_LEUKOCYTE_MEDIATED_IMMUNITY	0.5527545	2.112102	0	0.0001838
GOBP_MONOCYTE_CHEMOTAXIS	0.8395908	2.1080465	0	0.0002405
GOBP_RESPONSE_TO_CYTOKINE	0.54304755	2.1103032	0	0.0002485
GOBP_RESPONSE_TO_INTERLEUKIN_1	0.6611121	2.1044507	0	0.0003999
GOBP_CELLULAR_RESPONSE_TO_LIPID	0.58678347	2.092718	0	0.0004524
GOBP_LEUKOCYTE_MIGRATION	0.59830636	2.0802195	0	0.0005789
GOBP_NEGATIVE_REGULATION_OF_IMMUNE_SYSTEM_PROCE	0.62516034	2.0768552	0	0.0005923
GOBP_CELL_ACTIVATION_INVOLVED_IN_IMMUNE_RESPONSE	0.5486735	2.0825598	0	0.0005959

GOBP_ANTIMICROBIAL_HUMORAL_RESPONSE	0.8021241	2.060666	0	0.0009508

Top downregulated gene sets (FDR 1-val < 0.001)

NAME	ES	NES	NOM p-val	FDR q-val
GOBP_KERATINIZATION	-0.86071986	-2.643826	0	0
GOBP_CORNIFICATION	-0.85621953	-2.555769	0	0
GOBP_KERATINOCYTE_DIFFERENTIATION	-0.6450554	-2.4620523	0	0
GOBP_EPIDERMIS_DEVELOPMENT	-0.5858202	-2.4563222	0	0
GOBP_SKIN_DEVELOPMENT	-0.5762716	-2.4103231	0	0
GOBP_EPIDERMAL_CELL_DIFFERENTIATION	-0.5823767	-2.2783098	0	0.000275

Granulocytes chemotaxis genes

NAME	Rank in query	Rank score	Running ES	Core
CXCL2	0	4.79087925	0.1301681	Yes
CCL4	1	3.38842535	0.22223154	Yes
CCL3	3	3.19646096	0.3086982	Yes
CXCL1	4	2.95069981	0.38886866	Yes
S100A9	6	2.82037592	0.46511713	Yes
CCL2	7	2.58070827	0.5352349	Yes
S100A8	8	2.52109289	0.60373294	Yes
CD74	20	1.93156993	0.6520216	Yes
THBS1	26	1.6480118	0.6948926	Yes
FCER1G	27	1.6425066	0.7395194	Yes
CCL7	29	1.60775411	0.782821	Yes
CCL8	68	1.09109819	0.79798436	Yes
RAC2	127	0.74076706	0.79600734	Yes
CCL11	136	0.71085691	0.81227255	Yes
DPEP1	153	0.65135837	0.8238724	Yes
PDE4B	269	0.42622116	0.7916266	No
RARRES2	302	0.38402873	0.7898655	No
CSF1	577	0.19712624	0.69080067	No
LGALS3	659	0.16712788	0.66447264	No
PPIB	758	0.13297318	0.63073796	No
MAPK1	1324	-0.0924146	0.41792876	No
C1QBP	1358	-0.0965371	0.40797547	No
PPIA	2139	-0.228965	0.11694034	No
BSG	2185	-0.2442265	0.10642658	No
IL17RC	2477	-0.434586	0.00733489	No
CXADR	2501	-0.4742429	0.01145481	No
IL34	2535	-0.5231351	0.01309216	No
S100A14	2637	-1.1311857	0.0053356	No

Supplementary table 5: DEGs between cluster 3 in wound and cluster 3 in normal

Cluster 3 signature during wound healing (DEGs cluster 3 wound vs cluster 3 normal)

Highlighted in red: upregulated genes

Highlighted in green: downregulated genes

Gene name	p_val	avg_log2FC	p_val_adj	Type
Saa3	3.92264E-59	4.191680759	1.09826E-54	
Cxcl1	2.60436E-98	3.631174115	7.29169E-94	cytokine
Cxcl2	6.107E-149	3.56821022	1.7098E-144	cytokine
Ccl2	9.03239E-81	3.278205272	2.52889E-76	cytokine
Cthrc1	1.85817E-98	3.047800151	5.2025E-94	other
Ptx3	1.29787E-61	2.980753406	3.63379E-57	other
Sfrp2	4.3971E-81	2.765363813	1.2311E-76	transmembrane receptor
Plac8	5.1061E-106	2.610582457	1.4296E-101	other
Timp1	6.03759E-92	2.536775223	1.6904E-87	cytokine
Igfbp4	7.52096E-86	2.532389099	2.10572E-81	other
Cxcl5	5.17184E-37	2.367912309	1.44801E-32	cytokine
Gm12840	1.91897E-60	2.211760614	5.37273E-56	
Mgp	6.79109E-40	2.149023058	1.90137E-35	other
H19	3.29156E-43	2.030390979	9.21571E-39	other
Serpinb2	3.20557E-15	1.963670114	8.97496E-11	other
Ifi27l2a	1.49049E-81	1.946822551	4.17307E-77	other
Postn	7.51855E-68	1.878860227	2.10504E-63	other
Spp1	6.78628E-29	1.864610743	1.90002E-24	cytokine
Tnc	5.04575E-47	1.737219262	1.41271E-42	other
Acta2	5.72538E-50	1.683144824	1.60299E-45	other
Phlda1	2.44551E-56	1.67166342	6.84695E-52	other
Thbs4	6.89169E-20	1.614298848	1.92953E-15	other
Ccl7	2.34295E-37	1.539370171	6.5598E-33	cytokine
Cxcl10	5.26136E-27	1.50147117	1.47308E-22	cytokine
Cpxm1	5.39484E-61	1.445414182	1.51045E-56	peptidase
Cd44	2.64654E-50	1.384040304	7.40978E-46	other
Csrp2	1.37429E-61	1.382362396	3.84774E-57	other
Hist1h2bc	2.22963E-65	1.36480314	6.24251E-61	other
Nid1	3.69028E-78	1.240827276	1.0332E-73	other
Serpine1	1.39316E-21	1.227110787	3.90057E-17	other
Ier3	3.52022E-25	1.203599166	9.85592E-21	other
Rbp1	2.17608E-57	1.197118897	6.09258E-53	transporter
Il6	2.58779E-24	1.180002329	7.24528E-20	cytokine
Thbs1	1.86489E-42	1.177026066	5.22133E-38	other
Mfap5	3.68185E-36	1.166097641	1.03084E-31	other
Ptgs2	2.18729E-43	1.145064023	6.12399E-39	enzyme
Col14a1	7.96902E-54	1.136708509	2.23117E-49	other
Sfrp4	2.89304E-38	1.129813043	8.09994E-34	transmembrane receptor
Mt2	5.54861E-25	1.120017735	1.5535E-20	
Nfkbia	1.43692E-25	1.111088118	4.02309E-21	transcription regulator
Il33	2.26589E-50	1.090198254	6.34404E-46	cytokine
Mmp3	7.29514E-31	1.082566983	2.04249E-26	peptidase
Tpm2	7.31833E-45	1.080340248	2.04899E-40	other
S100a9	5.6445E-70	1.070779236	1.58035E-65	other

Il1rl1	7.46201E-28	1.065177775	2.08921E-23	transmembrane receptor
Angptl4	6.73125E-58	1.063092294	1.88462E-53	other
Rnd1	4.0542E-30	1.056544075	1.13509E-25	enzyme
Plat	2.15206E-65	1.055561284	6.02535E-61	peptidase
Tmem176b	1.31957E-49	1.049408879	3.69454E-45	other
Cd74	3.26647E-39	1.029218794	9.14546E-35	transmembrane receptor
Hist1h1c	1.77068E-45	1.021335265	4.95754E-41	other
Tmem176a	3.38809E-49	1.010080089	9.48596E-45	other
Islr	7.75722E-49	1.008485175	2.17187E-44	other
Maged2	4.88522E-52	0.991902546	1.36776E-47	other
Il1b	1.15402E-61	0.989193738	3.23102E-57	cytokine
Prg4	5.47611E-42	0.977260046	1.5332E-37	other
Bgn	1.27161E-36	0.931094306	3.56025E-32	other
Ifrd1	1.44559E-07	0.925407328	0.004047359	other
Tnfaip6	8.00165E-15	0.917195089	2.2403E-10	other
Sh3bgrl3	7.83132E-57	0.91371385	2.19261E-52	other
Slpi	3.66152E-47	0.907552936	1.02515E-42	other
Pkm	5.29083E-29	0.89655721	1.48133E-24	kinase
Ptn	2.66695E-23	0.890424838	7.46693E-19	growth factor
Col8a1	9.24447E-30	0.873292037	2.58827E-25	other
Ldha	3.24666E-37	0.869757323	9.09E-33	enzyme
Ccl4	6.25083E-49	0.86728869	1.75011E-44	cytokine
Gadd45a	3.3123E-38	0.861318544	9.27378E-34	other
Hk2	1.86626E-44	0.856435701	5.22517E-40	kinase
Hmox1	2.65353E-33	0.847137489	7.42935E-29	enzyme
Lgmn	1.84323E-57	0.845257676	5.16068E-53	peptidase
S100a8	4.08582E-49	0.8423177	1.14395E-44	other
Lrrc15	1.06169E-41	0.841754163	2.97253E-37	other
Noct	1.96311E-43	0.839953713	5.49632E-39	transcription regulator
Bst2	7.39596E-45	0.838286454	2.07072E-40	other
H2afz	2.75154E-18	0.831025947	7.70376E-14	other
Tm4sf1	3.07053E-37	0.828010313	8.59686E-33	other
Tnfaip3	2.2623E-24	0.819905106	6.33398E-20	enzyme
Isg15	1.88273E-28	0.816910267	5.27127E-24	other
Rcan1	5.23814E-18	0.816665075	1.46657E-13	other
Ptgis	3.42718E-47	0.812718181	9.59541E-43	enzyme
Sod3	2.36101E-37	0.811599361	6.61036E-33	enzyme
Ccl3	1.16163E-48	0.810650344	3.25232E-44	cytokine
Tsc22d1	1.38106E-45	0.807599318	3.86669E-41	transcription regulator
Gapdh	2.2026E-23	0.80386929	6.16684E-19	enzyme
H2-Ab1	1.21183E-42	0.803734554	3.39287E-38	
Fscn1	3.7246E-57	0.803095348	1.04281E-52	other
Efemp2	2.96707E-47	0.800211841	8.3072E-43	other
Mif	1.19623E-18	0.799148191	3.3492E-14	cytokine
2700094K13R	9.65133E-32	0.797117478	2.70218E-27	
Nov	6.07597E-24	0.7962322	1.70115E-19	
Nfkbiz	1.48715E-29	0.777473529	4.16373E-25	transcription regulator
Stmn1	8.69167E-25	0.77503964	2.43349E-20	other
Lox	4.12494E-32	0.773274527	1.1549E-27	enzyme

Gadd45b	2.51567E-28	0.770115556	7.04336E-24	other
Ppic	1.41802E-50	0.766806418	3.97017E-46	enzyme
Lpl	1.58327E-17	0.74945395	4.43285E-13	enzyme
Cenpa	4.17304E-21	0.748608524	1.16837E-16	other
Itih5	1.75038E-28	0.74777536	4.90071E-24	other
Plk2	1.23934E-25	0.7441972	3.4699E-21	kinase
Ddit4	2.33555E-31	0.736355576	6.53909E-27	other
Has1	1.62857E-13	0.734749722	4.55967E-09	enzyme
Figf	9.07004E-40	0.730197874	2.53943E-35	
Eno1	3.62742E-22	0.720953119	1.01561E-17	enzyme
Mxra8	5.85645E-42	0.719951135	1.63969E-37	other
Srgn	2.09596E-35	0.718293015	5.86826E-31	other
Lgals3bp	1.67462E-46	0.711594207	4.6886E-42	transmembrane receptor
Cks2	1.5506E-19	0.710584293	4.34136E-15	kinase
H2-Eb1	8.93855E-34	0.707908003	2.50262E-29	
Lsp1	1.50604E-35	0.702310534	4.21661E-31	other
Fabp4	6.17783E-19	0.702262711	1.72967E-14	transporter
Pla1a	1.79829E-23	0.700480665	5.03485E-19	enzyme
Abracl	1.00286E-44	0.698540437	2.8078E-40	other
Ace	6.5708E-29	0.696615907	1.83969E-24	peptidase
Col12a1	2.9139E-27	0.696403442	8.15833E-23	other
Ifitm1	1.49296E-26	0.692680406	4.17998E-22	transmembrane receptor
Prelid1	1.7326E-37	0.682988395	4.85093E-33	other
Csrp1	1.05522E-31	0.677434646	2.9544E-27	other
Hilpda	3.90926E-26	0.674457046	1.09451E-21	other
Eif3f	5.88056E-60	0.672536269	1.64644E-55	translation regulator
Srpx2	1.18084E-50	0.665771182	3.30611E-46	other
S100a11	2.67667E-41	0.658537749	7.49413E-37	other
Tpi1	1.96021E-32	0.652114599	5.48818E-28	enzyme
Serpine2	4.40784E-26	0.651583725	1.23411E-21	other
H2-Aa	9.87277E-31	0.650670294	2.76418E-26	
Osr1	8.51971E-35	0.637896468	2.38535E-30	transcription regulator
Tagln2	4.61926E-22	0.634400173	1.2933E-17	other
F3	2.07296E-27	0.632886189	5.80387E-23	transmembrane receptor
Col6a3	1.49278E-31	0.632205336	4.17948E-27	other
Marcksl1	7.56992E-42	0.631999162	2.11943E-37	other
1810011O10R	9.3573E-25	0.631755809	2.61986E-20	
Ccl8	6.09197E-23	0.628524864	1.70563E-18	cytokine
Tshz2	5.48432E-42	0.62437035	1.5355E-37	other
Rpl22l1	2.02927E-53	0.618109174	5.68155E-49	other
Gpx7	1.65441E-26	0.614744559	4.63202E-22	enzyme
Rhoc	2.39612E-40	0.614602036	6.70866E-36	enzyme
Ccnb2	6.36078E-23	0.612207516	1.78089E-18	other
Gas7	1.05098E-21	0.609743595	2.94252E-17	
Emb	6.3435E-30	0.60696254	1.77605E-25	other
P4ha3	2.88892E-49	0.60537954	8.08839E-45	enzyme
Col5a3	8.02876E-27	0.604833378	2.24789E-22	other
Tpm1	5.19682E-20	0.597582539	1.45501E-15	other
Mdk	1.76356E-24	0.590932552	4.93761E-20	growth factor

Fabp5	1.50203E-28	0.590821472	4.20539E-24	transporter
Pabpc1	8.02228E-37	0.589420925	2.24608E-32	translation regulator
Vcam1	1.60789E-31	0.587335368	4.50178E-27	transmembrane receptor
Col6a2	9.12461E-27	0.587232071	2.55471E-22	other
Sbsn	1.58205E-12	0.586869155	4.42942E-08	other
Mt1	3.27347E-14	0.585908132	9.16505E-10	
Angptl2	4.37482E-32	0.585212953	1.22486E-27	other
Bhlhe40	9.45426E-28	0.58091148	2.647E-23	transcription regulator
Birc5	1.02299E-20	0.57717135	2.86418E-16	other
Rpl18a	5.65075E-78	0.5724161	1.5821E-73	other
Irf7	1.27798E-29	0.572159193	3.57808E-25	transcription regulator
Cfl1	1.08432E-22	0.569624772	3.03589E-18	other
Slc25a4	9.7435E-44	0.567148817	2.72799E-39	transporter
Cald1	5.21563E-25	0.566412448	1.46027E-20	other
Sqstm1	3.49662E-20	0.565978812	9.78982E-16	transcription regulator
Sulf1	2.25418E-43	0.564905318	6.31125E-39	enzyme
Loxl3	8.12564E-45	0.557841358	2.27502E-40	enzyme
Eva1b	1.19386E-33	0.556686191	3.34256E-29	other
Tubb2b	3.04022E-35	0.556196554	8.512E-31	other
Col5a1	4.99095E-27	0.555966662	1.39736E-22	other
Nt5dc2	2.13075E-38	0.553270155	5.96567E-34	other
Cfb	2.00778E-29	0.550423863	5.62139E-25	peptidase
Fibin	9.9169E-21	0.546193537	2.77653E-16	other
Ifi203	2.90081E-31	0.543418619	8.1217E-27	
Rpl32	2.38598E-71	0.541581666	6.68026E-67	other
Arpc1b	2.40678E-36	0.536079388	6.7385E-32	other
Rpl29	1.98726E-42	0.531388333	5.56393E-38	other
Lamb1	6.90242E-39	0.530430374	1.93254E-34	other
Ptp4a1	3.52668E-12	0.527102699	9.87401E-08	phosphatase
Actn1	2.0048E-35	0.524540054	5.61303E-31	transcription regulator
Runx1	2.33034E-38	0.523425559	6.52447E-34	transcription regulator
Tpm4	6.57548E-14	0.522695749	1.841E-09	other
Lgals1	1.49348E-19	0.518643081	4.18144E-15	other
Eef1b2	2.34607E-32	0.518038443	6.56853E-28	translation regulator
Sox4	2.82721E-29	0.517450541	7.91561E-25	transcription regulator
Sod2	7.81699E-16	0.515128863	2.1886E-11	enzyme
Myc	1.5917E-16	0.512873017	4.45643E-12	transcription regulator
H2afv	5.71143E-23	0.510909457	1.59909E-18	other
Hif1a	3.00134E-26	0.505711092	8.40315E-22	transcription regulator
Ngf	1.39728E-20	0.49945084	3.9121E-16	growth factor
Mndal	6.25419E-31	0.498895294	1.75105E-26	
Rps19	4.98439E-67	0.49713858	1.39553E-62	other
Fstl1	8.53397E-21	0.496909246	2.38934E-16	other
Ccdc34	1.01331E-26	0.49506608	2.83706E-22	other
Nrep	3.82941E-32	0.49274276	1.07216E-27	other
Ly6e	1.51331E-20	0.491462572	4.23698E-16	other
Fxyd5	2.59268E-22	0.491151506	7.25899E-18	ion channel
Rpl10a	3.50901E-47	0.490076085	9.82453E-43	other
Mmp19	4.59375E-34	0.489574014	1.28616E-29	peptidase

P4hb	1.61963E-22	0.489387279	4.53464E-18	enzyme
Prdx5	7.66699E-26	0.48819726	2.1466E-21	enzyme
Col4a2	2.23951E-16	0.487079962	6.27017E-12	other
Rplp0	3.36668E-53	0.485678211	9.42603E-49	other
Basp1	7.18769E-23	0.485049328	2.01241E-18	transcription regulator
Cstb	3.3025E-24	0.484630702	9.24635E-20	peptidase
Foxp1	2.24218E-37	0.483569265	6.27767E-33	transcription regulator
Cnn2	2.42094E-20	0.482553928	6.77814E-16	other
Gng11	1.19971E-20	0.481440574	3.35894E-16	other
Phf11d	1.58995E-28	0.48128321	4.45155E-24	
Prss23	3.29384E-15	0.480251694	9.22209E-11	peptidase
Tyrobp	1.63795E-23	0.479885333	4.58593E-19	transmembrane receptor
Abca8a	4.95778E-12	0.476982465	1.38808E-07	
Col5a2	5.2824E-23	0.474160769	1.47897E-18	other
1810058I24Ri	1.57026E-28	0.474039339	4.39641E-24	
Rtp4	3.05385E-30	0.473533496	8.55018E-26	other
Pamr1	1.56089E-24	0.472754621	4.37018E-20	peptidase
Col3a1	1.21686E-18	0.472604537	3.40698E-14	other
Pdlim7	1.22062E-33	0.470746935	3.4175E-29	other
Rpl13a	1.52659E-46	0.468897862	4.27414E-42	other
Man2a1	6.44233E-32	0.467742111	1.80372E-27	enzyme
Spry2	2.3519E-14	0.465348879	6.58485E-10	other
Rhoj	1.04724E-22	0.465135456	2.93207E-18	enzyme
Ywhah	3.70025E-19	0.463989331	1.03599E-14	transcription regulator
Dpysl3	1.2174E-23	0.463413432	3.40848E-19	enzyme
Aldoa	1.42706E-17	0.458838328	3.99549E-13	enzyme
C1qtnf6	6.01128E-26	0.457386677	1.68304E-21	other
Chmp4b	4.40076E-25	0.456527237	1.23213E-20	other
Abi3bp	1.84042E-19	0.454848259	5.15281E-15	other
Fxyd6	1.05409E-15	0.452003708	2.95124E-11	ion channel
Gnpnat1	1.25425E-31	0.451807186	3.51165E-27	enzyme
Rps3	1.30657E-48	0.450604578	3.65812E-44	enzyme
Ap3s1	3.83529E-27	0.449830426	1.07381E-22	transporter
Slc39a14	4.87657E-21	0.449228473	1.36534E-16	transporter
Rpl41	2.53983E-43	0.444263179	7.11101E-39	other
Cdca3	6.51146E-21	0.44382732	1.82308E-16	other
Ecscr	1.44639E-30	0.442242137	4.04962E-26	other
Cotl1	2.34752E-30	0.441105878	6.57258E-26	other
Lhfp	9.75626E-26	0.440117282	2.73156E-21	
Rpl24	1.4503E-51	0.438334588	4.06056E-47	other
Plagl1	2.74194E-24	0.436999326	7.67688E-20	transcription regulator
Sri	4.9931E-27	0.434906504	1.39797E-22	transporter
Fhl2	2.91119E-32	0.434238067	8.15074E-28	transcription regulator
Adamts15	7.07344E-27	0.433467018	1.98042E-22	peptidase
Ext1	2.70857E-25	0.431822355	7.58347E-21	enzyme
Npm1	8.26222E-22	0.430514625	2.31326E-17	transcription regulator
Rps15a	1.516E-40	0.428957549	4.24451E-36	other
Cnn3	1.07528E-17	0.428022119	3.01058E-13	other
Nfkb1	4.12545E-20	0.427418555	1.15504E-15	transcription regulator

Pdlim1	1.93967E-14	0.426852072	5.43068E-10	transcription regulator
Tax1bp3	7.66432E-24	0.424008575	2.14586E-19	transcription regulator
Plod2	2.7842E-28	0.421190931	7.79521E-24	enzyme
Sdc1	9.67923E-23	0.420142073	2.70999E-18	enzyme
Fbln2	8.54981E-17	0.41858037	2.39378E-12	other
Serf2	5.76483E-32	0.418200413	1.61404E-27	other
Cp	7.05716E-19	0.417997494	1.97586E-14	enzyme
Txn1	1.6913E-22	0.417757568	4.73529E-18	
Ackr3	2.4755E-10	0.416051285	6.93092E-06	G-protein coupled receptor
Cox7a2l	1.75605E-22	0.415020427	4.9166E-18	enzyme
Tspan6	1.8747E-32	0.414195078	5.24877E-28	other
Wisp1	1.26434E-28	0.413809911	3.5399E-24	
Acta1	5.55972E-34	0.413210155	1.55661E-29	other
Thbd	2.45751E-15	0.413045371	6.88055E-11	transmembrane receptor
Rps18	4.20398E-49	0.412883711	1.17703E-44	other
Fkbp10	7.37951E-12	0.408536078	2.06612E-07	enzyme
Fcer1g	3.09445E-22	0.407467895	8.66385E-18	transmembrane receptor
Eif3e	5.91546E-22	0.407420327	1.65621E-17	other
Gnb2l1	1.47618E-22	0.406041674	4.13301E-18	
2700060E02R	1.69386E-26	0.405947151	4.74248E-22	
Adam12	1.31765E-30	0.40583894	3.68917E-26	peptidase
Steap4	1.52355E-22	0.404795037	4.26564E-18	enzyme
Ccdc109b	7.67542E-24	0.404054249	2.14897E-19	
Agpat4	6.254E-28	0.401706116	1.75099E-23	enzyme
Col6a1	9.0429E-17	0.401002336	2.53183E-12	other
Rpl23	3.47975E-27	0.400929494	9.74262E-23	other
Adora2b	8.60867E-23	0.399464673	2.41025E-18	G-protein coupled receptor
Esd	5.94765E-20	0.396023824	1.66522E-15	enzyme
Hsph1	1.38631E-16	0.395386391	3.8814E-12	other
Rgs3	6.49105E-19	0.395273291	1.81737E-14	enzyme
Procr	4.78425E-27	0.394071723	1.33949E-22	other
Dnaja1	2.47198E-14	0.39020751	6.92106E-10	other
Enc1	3.85846E-20	0.389698723	1.08029E-15	peptidase
Eif4ebp1	3.60117E-18	0.389588945	1.00826E-13	translation regulator
Ddit3	4.28937E-15	0.387809802	1.20094E-10	transcription regulator
Dap	1.93852E-14	0.387239519	5.42748E-10	transcription regulator
Sparc	2.11613E-18	0.385336218	5.92475E-14	other
Rras2	1.37723E-13	0.385147216	3.85598E-09	enzyme
Plxdc2	2.29533E-22	0.379324884	6.42647E-18	other
Rps11	3.64575E-40	0.378599314	1.02074E-35	other
Akr1b8	2.00566E-31	0.37853999	5.61545E-27	
Etfa	8.98042E-23	0.378225763	2.51434E-18	transporter
Pdlim2	1.20849E-19	0.377541012	3.38352E-15	other
Gpi1	2.36873E-21	0.37685827	6.63197E-17	
Pkig	7.11427E-26	0.37682603	1.99185E-21	other
Glipr2	2.53394E-18	0.375765663	7.09452E-14	other
Loxl2	1.55195E-12	0.375559211	4.34516E-08	enzyme
Ypel3	9.65259E-15	0.373157115	2.70253E-10	other
Rps20	1.53886E-17	0.372468066	4.30851E-13	other

Higd1a	1.29782E-07	0.371814041	0.003633627	other
Vim	5.39121E-14	0.371582127	1.50943E-09	other
Snhg6	2.05794E-21	0.370632849	5.76182E-17	other
Gpx8	3.97429E-17	0.37015822	1.11272E-12	enzyme
Rpl17	4.40466E-33	0.369578659	1.23322E-28	other
Rpl3	2.06389E-37	0.368424239	5.77847E-33	other
Eef1g	7.06035E-20	0.367016418	1.97676E-15	translation regulator
Rpsa	4.28302E-22	0.366924946	1.19916E-17	translation regulator
Lrrc17	7.13701E-16	0.366189226	1.99822E-11	other
Rps3a1	8.28251E-37	0.366145304	2.31894E-32	
Fndc1	3.10675E-10	0.365232803	8.69828E-06	other
Rps6	3.76322E-38	0.364447438	1.05363E-33	other
Rps8	4.82258E-28	0.362996116	1.35023E-23	other
Rps14	1.30295E-39	0.362789601	3.64801E-35	translation regulator
Nrp1	3.34706E-21	0.362723466	9.37111E-17	transmembrane receptor
Eif4e	9.38701E-10	0.362102801	2.62817E-05	translation regulator
Rpl4	6.64806E-34	0.36114379	1.86132E-29	enzyme
Col4a1	1.1484E-07	0.360845438	0.003215283	other
Arl4c	2.84185E-21	0.360816797	7.95661E-17	enzyme
Atp5g2	1.91263E-18	0.360199675	5.35499E-14	
Hacd1	2.87144E-27	0.359652093	8.03944E-23	enzyme
Bicc1	7.85067E-20	0.359597505	2.19803E-15	other
Grb10	2.45108E-18	0.359523817	6.86254E-14	other
Rdh10	6.10289E-19	0.358858411	1.70869E-14	enzyme
Prdx2	1.27943E-17	0.357575849	3.58216E-13	enzyme
Myl9	8.34781E-14	0.357399565	2.33722E-09	other
Gem	4.00086E-11	0.357273369	1.12016E-06	enzyme
Gm1673	5.75309E-31	0.355833342	1.61075E-26	
Pxdn	1.94182E-24	0.355585835	5.4367E-20	enzyme
Eif3i	5.19814E-20	0.355585361	1.45537E-15	translation regulator
Rpl34	1.76709E-34	0.355153817	4.94749E-30	other
Fap	1.50849E-21	0.353772988	4.22347E-17	peptidase
Smim3	7.28137E-29	0.352746798	2.03864E-24	ion channel
Spsb1	2.70679E-22	0.352182956	7.57848E-18	other
Rpl7	6.35341E-27	0.352152865	1.77883E-22	transcription regulator
Eif4e2	5.43134E-21	0.350439253	1.52067E-16	translation regulator
App	2.77641E-20	0.350069348	7.7734E-16	other
Hspa8	4.64664E-10	0.348105097	1.30097E-05	enzyme
Loxl1	4.30595E-13	0.347913304	1.20558E-08	enzyme
Ehd1	4.93025E-11	0.347368092	1.38037E-06	other
Nxn	2.3077E-28	0.346585584	6.46109E-24	enzyme
Msrb1	1.69245E-25	0.346015032	4.73852E-21	enzyme
Map1lc3b	8.1407E-17	0.346013045	2.27923E-12	other
Myl12a	6.79416E-14	0.345742569	1.90223E-09	other
Pfn1	4.39068E-11	0.343285358	1.2293E-06	other
Rpl36a	1.08342E-24	0.341713833	3.03336E-20	other
Cfh	2.99091E-18	0.340844567	8.37394E-14	other
Fbn2	6.83133E-25	0.340234208	1.91263E-20	other
Rab13	3.36452E-24	0.339967453	9.41998E-20	enzyme

Scarb2	1.25744E-24	0.339039654	3.52058E-20	transmembrane receptor
Ugp2	7.84007E-13	0.338689078	2.19506E-08	enzyme
Cdkn1c	8.16802E-10	0.33823577	2.28688E-05	other
Pgls	8.11246E-22	0.338079354	2.27133E-17	
Wnt2	1.86566E-20	0.337549684	5.22347E-16	cytokine
Srxn1	1.82429E-09	0.337432273	5.10765E-05	enzyme
Rpl12	1.77414E-10	0.336421266	4.96724E-06	other
Mfap2	1.58281E-07	0.33587039	0.004431558	other
Oasl2	2.21666E-20	0.335731126	6.20621E-16	
Pgam1	1.0649E-09	0.335585435	2.98151E-05	phosphatase
Rps16	2.47744E-26	0.335281109	6.93634E-22	other
Cdk4	5.12739E-18	0.333740307	1.43557E-13	kinase
Dynlt1f	8.41728E-28	0.329349069	2.35667E-23	
Ppp1ca	1.07524E-13	0.329026399	3.01046E-09	phosphatase
Dact1	9.39767E-21	0.328954682	2.63116E-16	other
Plin2	4.34135E-15	0.327414206	1.21549E-10	other
Tmsb4x	4.99008E-10	0.327400328	1.39712E-05	other
Slfn2	2.288E-22	0.326792539	6.40593E-18	
Eny2	5.14636E-15	0.325793007	1.44088E-10	transcription regulator
Eif3h	6.5833E-20	0.325784943	1.84319E-15	other
Zfos1	6.89E-14	0.325260455	1.92906E-09	
Fst	2.70115E-09	0.324092669	7.56269E-05	other
Serf1	4.50239E-20	0.322740634	1.26058E-15	
Cxcl16	6.77098E-24	0.322048817	1.89574E-19	cytokine
Gsto1	2.9405E-20	0.320807475	8.23281E-16	enzyme
Eef2	4.42392E-18	0.320223308	1.23861E-13	translation regulator
Lgi2	4.04415E-22	0.318774527	1.13228E-17	other
Rpl6	1.07329E-27	0.318237963	3.005E-23	
Rnf19b	1.05628E-16	0.317620719	2.95736E-12	enzyme
Prdx4	1.79583E-10	0.317586635	5.02796E-06	enzyme
Ap2s1	3.41933E-15	0.317500453	9.57344E-11	transporter
Plscr1	3.85965E-24	0.317078836	1.08062E-19	enzyme
Lyz2	5.30663E-12	0.316936051	1.48575E-07	
Heg1	8.78711E-12	0.316852404	2.46022E-07	other
Rps9	5.283E-30	0.316747413	1.47913E-25	translation regulator
Rps2	5.66749E-15	0.316737833	1.58678E-10	other
Rps4x	4.73817E-23	0.315819099	1.32659E-18	other
Ier5	5.1957E-10	0.315386672	1.45469E-05	other
Rps7	1.27746E-26	0.315279906	3.57664E-22	other
Rpl27a	6.41932E-22	0.315162423	1.79728E-17	other
Rpl18	1.07766E-24	0.314628163	3.01723E-20	other
Capzb	8.59267E-14	0.314565344	2.40577E-09	other
Vcan	4.91743E-15	0.314260706	1.37678E-10	other
Rhoq	1.26769E-21	0.314228181	3.54927E-17	enzyme
Svbp	5.17064E-19	0.314063298	1.44768E-14	other
Mrpl34	1.85494E-24	0.313512865	5.19347E-20	other
Ifit1	2.53713E-09	0.312698797	7.10345E-05	other
Ninj1	2.09917E-23	0.310546765	5.87724E-19	other
Zbp1	1.06595E-12	0.310451822	2.98445E-08	other

Hspe1	1.30497E-07	0.309138863	0.003653647	enzyme
Nid2	8.62065E-21	0.308437947	2.41361E-16	other
Atpif1	4.45091E-14	0.30835665	1.24617E-09	
Gpm6b	3.94681E-15	0.307441183	1.10503E-10	other
Ebf2	4.25164E-22	0.307068269	1.19037E-17	transcription regulator
Mmp11	2.05905E-09	0.306544578	5.76493E-05	peptidase
Rps27a	3.3352E-24	0.304912268	9.3379E-20	other
Acaa2	1.25378E-18	0.304191172	3.51032E-14	enzyme
P4ha1	1.69814E-16	0.303391759	4.75445E-12	enzyme
Lxn	3.19785E-21	0.30227359	8.95335E-17	other
Plp2	7.88477E-18	0.301231979	2.20758E-13	transporter
Mrpl30	7.67164E-15	0.300852788	2.1479E-10	other
Ndufa6	2.57565E-13	0.299714927	7.21131E-09	enzyme
Gpc6	7.62186E-21	0.299470948	2.13397E-16	transmembrane receptor
Tomm20	1.3672E-17	0.299314256	3.82788E-13	transporter
Eif3k	8.80208E-17	0.298746986	2.46441E-12	translation regulator
Pdia5	1.75156E-18	0.298566274	4.90401E-14	enzyme
Pcsk5	2.35975E-13	0.298100205	6.60681E-09	peptidase
Rpl37	9.78682E-25	0.297587939	2.74011E-20	other
Nsa2	1.08439E-15	0.297349565	3.03608E-11	other
Ifi30	2.93107E-19	0.296966979	8.20641E-15	enzyme
Mettl9	1.4699E-16	0.296442052	4.11542E-12	other
Akr1a1	9.73893E-12	0.296003937	2.72671E-07	enzyme
Phlda3	3.74044E-14	0.295910094	1.04725E-09	other
Nfe2l2	2.11617E-11	0.295795641	5.92486E-07	transcription regulator
Ttyh2	1.84413E-20	0.295257625	5.1632E-16	ion channel
Rpl26	7.13605E-19	0.294908529	1.99795E-14	other
Tmem119	5.97091E-17	0.294879018	1.67174E-12	other
Ifi204	2.78099E-18	0.294715503	7.78622E-14	
Rgs10	9.10722E-20	0.294606433	2.54984E-15	enzyme
Slc43a3	3.03885E-12	0.293937492	8.50816E-08	other
Rps27rt	3.52783E-12	0.292903094	9.87723E-08	
B4galt5	1.64046E-20	0.292049381	4.59297E-16	enzyme
AI607873	9.49302E-15	0.291919898	2.65785E-10	
Peg3	2.11243E-08	0.291364114	0.000591439	kinase
Dkk3	3.1824E-19	0.290850037	8.9101E-15	cytokine
Gpsm3	8.36584E-21	0.28865107	2.34227E-16	other
Eif3m	1.39357E-16	0.287976645	3.90171E-12	other
Dbn1	1.65139E-17	0.287565777	4.62356E-13	other
Fau	1.95611E-14	0.286302546	5.47671E-10	other
Sep15	1.23979E-13	0.286249453	3.47118E-09	
Tnfaip2	7.91565E-07	0.286148848	0.022162234	other
Matn2	1.37929E-18	0.285771071	3.86173E-14	other
Acp5	2.79834E-22	0.28550014	7.83478E-18	phosphatase
Ptms	7.00267E-11	0.285013927	1.96061E-06	other
Meox2	7.16593E-18	0.284913091	2.00632E-13	transcription regulator
Lima1	5.3311E-17	0.284553326	1.4926E-12	other
Ngfrap1	2.84938E-14	0.284420732	7.97768E-10	
Pofut2	8.76296E-14	0.284264024	2.45345E-09	enzyme

Rpl14	7.15602E-25	0.283909341	2.00354E-20	other
Vmp1	4.18449E-13	0.283779301	1.17157E-08	other
Zfhx3	5.05216E-20	0.283465599	1.4145E-15	transcription regulator
Adprh	8.82447E-16	0.283432146	2.47067E-11	enzyme
Eef1a1	6.26481E-22	0.283287172	1.75402E-17	translation regulator
Dcakd	3.25553E-22	0.282904573	9.11482E-18	other
Ptgr1	5.70486E-22	0.281449632	1.59725E-17	enzyme
Il18	2.22264E-14	0.281187467	6.22294E-10	cytokine
Ssc5d	2.07797E-21	0.281086335	5.81791E-17	transmembrane receptor
Cyp1b1	2.18388E-14	0.281012684	6.11444E-10	enzyme
Tfpi2	3.12761E-21	0.280642204	8.75669E-17	other
Tceb1	3.5314E-11	0.279491533	9.88723E-07	
Nans	3.56165E-14	0.27919372	9.97191E-10	enzyme
Sgk1	5.56549E-09	0.278500816	0.000155822	kinase
Rpl7a	8.43681E-16	0.278075082	2.36214E-11	other
Uqcrfs1	4.75677E-15	0.277255114	1.3318E-10	enzyme
Dcbld2	2.13457E-20	0.277211442	5.97637E-16	other
Spats2l	1.49645E-19	0.276877257	4.18977E-15	other
Rnf149	3.09029E-19	0.275998367	8.6522E-15	enzyme
Rps27	1.56926E-23	0.275828465	4.39362E-19	other
Ugcg	3.59794E-17	0.275718714	1.00735E-12	enzyme
Sub1	8.72276E-12	0.275117799	2.4422E-07	transcription regulator
Vasp	2.10789E-19	0.274757939	5.90168E-15	other
Rsu1	5.78196E-14	0.274537543	1.61883E-09	other
Prelp	1.14433E-06	0.271849682	0.032038989	other
Prkar1a	7.06995E-12	0.271690527	1.97944E-07	kinase
Fhl1	4.1124E-08	0.271083711	0.00115139	other
Jag1	2.29255E-18	0.27089029	6.41869E-14	growth factor
Hmgb2	2.845E-07	0.270039217	0.007965435	transcription regulator
BC028528	1.06436E-18	0.269805999	2.98E-14	
Rpl31	1.68337E-13	0.269648792	4.7131E-09	other
Rpl9	1.57384E-21	0.268762759	4.40643E-17	other
Col27a1	1.00092E-20	0.268266637	2.80237E-16	other
Fam129a	1.42573E-22	0.268209157	3.99176E-18	other
Uqcrh	8.83619E-14	0.267953294	2.47396E-09	enzyme
Rps26	3.42401E-11	0.266564504	9.58654E-07	other
Rap1a	6.06564E-09	0.264802426	0.000169826	enzyme
H1f0	1.19148E-06	0.264738282	0.033358933	other
Adam19	2.87322E-18	0.264576352	8.04445E-14	peptidase
Nek6	3.7082E-23	0.264301834	1.03822E-18	kinase
Birc3	4.54326E-13	0.264297526	1.27202E-08	enzyme
Ssbp4	9.49317E-20	0.26426876	2.6579E-15	transcription regulator
Smpdl3a	1.68136E-11	0.263907659	4.70746E-07	enzyme
Slc35b2	6.0825E-19	0.263432729	1.70298E-14	transporter
Atp5f1	3.29005E-11	0.262443252	9.21148E-07	
Bmp1	6.2229E-11	0.262307342	1.74229E-06	peptidase
P3h1	1.89852E-14	0.261837129	5.31548E-10	enzyme
Rpl15	1.78271E-19	0.261069634	4.99122E-15	other
Csgalnact1	1.83999E-21	0.261025629	5.15161E-17	enzyme

Scn7a	3.27375E-14	0.260777902	9.16585E-10	ion channel
Gbp3	1.49396E-16	0.258099136	4.18278E-12	enzyme
Glul	1.35471E-09	0.257886032	3.79293E-05	enzyme
Rpl37a	1.51012E-21	0.256540841	4.22802E-17	other
Nudcd2	2.25204E-16	0.256530168	6.30526E-12	other
Ubald2	7.23962E-18	0.256079628	2.02695E-13	other
Trim30a	6.81939E-14	0.255247672	1.90929E-09	
Wbp5	5.50252E-10	0.254994468	1.5406E-05	
Fkbp11	1.25423E-07	0.254208347	0.003511593	enzyme
Arpc2	2.50986E-09	0.253945883	7.0271E-05	other
Bcat1	1.29208E-18	0.253929164	3.61757E-14	enzyme
Rgs1	9.27658E-14	0.253920341	2.59726E-09	enzyme
Rab32	3.86731E-19	0.253254182	1.08277E-14	enzyme
Praf2	1.02707E-12	0.252895043	2.87558E-08	other
Bnip3	2.52751E-14	0.252714398	7.07651E-10	other
Dcun1d5	7.17017E-12	0.252517743	2.0075E-07	other
Map4k4	1.71852E-13	0.252486526	4.81153E-09	kinase
Bmper	1.50214E-16	0.252264709	4.20569E-12	other
Igf2r	5.07193E-21	0.251846058	1.42004E-16	transmembrane receptor
Rps5	1.85849E-19	0.251599521	5.20341E-15	other
Cmpk1	7.82783E-15	0.250675103	2.19163E-10	kinase
Sh3bgrl	1.18981E-14	0.247535713	3.33123E-10	other
Il3ra	7.11428E-15	0.246658939	1.99185E-10	transmembrane receptor
Hist1h4i	1.99869E-19	0.24611383	5.59594E-15	other
Pla2g16	5.04179E-11	0.245767094	1.4116E-06	
Nudt4	6.97194E-14	0.244746492	1.952E-09	phosphatase
Plekhf1	3.54007E-13	0.244209878	9.91149E-09	other
Epdr1	3.28565E-18	0.243369157	9.19917E-14	other
Mtpn	3.2268E-14	0.242207682	9.03438E-10	transcription regulator
Rpl28	1.27937E-08	0.240926034	0.000358198	other
Zyx	1.07531E-10	0.240773337	3.01066E-06	other
Hspd1	3.84493E-07	0.240552722	0.010765048	enzyme
Idh2	1.05258E-11	0.240018694	2.94703E-07	enzyme
Rps17	9.7756E-10	0.239991215	2.73697E-05	other
Atp6v1f	1.39043E-10	0.239851999	3.89293E-06	enzyme
Emp1	1.90964E-07	0.239208808	0.005346609	other
Ogfr	6.76846E-15	0.238993226	1.89503E-10	other
Fundc2	5.13177E-11	0.238672548	1.43679E-06	other
Tpbg	4.27208E-19	0.238534379	1.1961E-14	other
Pold4	2.34817E-13	0.23828451	6.57442E-09	enzyme
Rpl5	8.50497E-09	0.237927683	0.000238122	other
Txndc5	2.39806E-08	0.237596463	0.00067141	enzyme
Gpx4	1.79564E-11	0.237546623	5.02744E-07	enzyme
Raph1	2.10473E-13	0.236433699	5.89282E-09	other
Slc3a2	5.60072E-09	0.236237425	0.000156809	transporter
Ech1	3.42117E-10	0.235913466	9.57859E-06	enzyme
Rbm17	1.13884E-14	0.235908998	3.18851E-10	other
Rbms2	5.11748E-17	0.235853449	1.43279E-12	other
Bckdhb	1.11611E-10	0.233971436	3.12488E-06	enzyme

Lmnb1	3.71184E-18	0.233136022	1.03924E-13	other
Cmtm7	9.10895E-17	0.233043072	2.55032E-12	cytokine
Hcfc1r1	3.73011E-08	0.232080819	0.001044355	other
Casp6	3.02949E-21	0.231794657	8.48197E-17	peptidase
Bach1	2.11395E-16	0.231751315	5.91863E-12	transcription regulator
Smim1	2.32607E-14	0.231635678	6.51254E-10	other
Rpl11	7.91977E-15	0.231605514	2.21738E-10	other
Rpl22	7.75688E-10	0.231540345	2.17177E-05	translation regulator
2610524H06R	1.34911E-19	0.231470476	3.77723E-15	
Mlf2	1.55499E-10	0.229364144	4.35365E-06	other
Rpl19	7.49944E-16	0.229170173	2.09969E-11	other
Paip2	8.57728E-09	0.229048081	0.000240147	translation regulator
Lamc1	1.24643E-07	0.228693502	0.003489743	other
Ak2	8.96946E-14	0.228468771	2.51127E-09	kinase
Gch1	4.10953E-16	0.226944575	1.15059E-11	enzyme
Edem1	1.77654E-18	0.226695119	4.97395E-14	enzyme
Nfkbib	3.90851E-14	0.226646736	1.09431E-09	transcription regulator
Uck2	8.12241E-18	0.226330444	2.27411E-13	kinase
Plod3	1.63495E-13	0.226241622	4.57752E-09	enzyme
Dhrs3	7.71782E-19	0.225945279	2.16083E-14	enzyme
Plod1	4.02572E-13	0.225626253	1.12712E-08	enzyme
Mdh2	3.53966E-08	0.225316562	0.000991034	enzyme
Shfm1	1.17948E-10	0.225090772	3.30232E-06	
Litaf	3.22473E-12	0.225069354	9.02861E-08	transcription regulator
Stx11	6.27418E-15	0.224865854	1.75664E-10	transporter
Rbp4	4.98652E-10	0.223439416	1.39613E-05	other
Hacd2	1.03447E-14	0.223340476	2.89632E-10	phosphatase
Lipa	1.05658E-16	0.223244344	2.95822E-12	enzyme
Atp5l	4.5023E-12	0.222870697	1.26055E-07	
Iah1	8.36169E-14	0.222811965	2.34111E-09	other
Plaur	1.05128E-10	0.222226444	2.94337E-06	transmembrane receptor
Btf3	3.89529E-11	0.221394949	1.0906E-06	transcription regulator
Fam195b	3.46E-09	0.220705814	9.68731E-05	
Syngr2	1.80547E-16	0.220536028	5.05495E-12	other
Glrx3	2.24882E-12	0.220330683	6.29623E-08	enzyme
Tpt1	4.38704E-07	0.220127516	0.012282844	other
Arf5	2.21454E-10	0.220102713	6.20027E-06	enzyme
Erh	2.50174E-07	0.219980969	0.007004363	other
Chchd5	1.5965E-16	0.219492644	4.46988E-12	other
Itga5	9.11475E-12	0.218794652	2.55195E-07	transmembrane receptor
Pck2	2.75396E-18	0.218777195	7.71054E-14	kinase
Rps23	1.39593E-13	0.218070391	3.90833E-09	translation regulator
Ube2j2	7.62992E-14	0.216970988	2.13622E-09	enzyme
Tspo	3.3682E-10	0.216958908	9.43028E-06	transmembrane receptor
Rcan2	3.40862E-16	0.216695454	9.54346E-12	other
Psmb1	3.98398E-10	0.216627817	1.11544E-05	peptidase
G0s2	1.23059E-08	0.216044808	0.00034454	other
Tmem173	7.80703E-17	0.215994168	2.18581E-12	other
Rras	1.72452E-12	0.215854372	4.8283E-08	enzyme

Ube2l3	3.79566E-11	0.215473568	1.06271E-06	enzyme
Slc4a4	2.36197E-12	0.214423319	6.61304E-08	transporter
Gdi2	2.98439E-07	0.214094002	0.008355702	other
Prdx1	3.81948E-07	0.213153086	0.01069377	enzyme
Cnih4	6.31466E-14	0.213042715	1.76798E-09	other
Cdr2l	5.55948E-19	0.212880984	1.55654E-14	other
Tmem167	1.22885E-07	0.212880223	0.003440523	
Rpl13	2.61824E-18	0.212817792	7.33056E-14	other
Enah	1.40104E-16	0.212400155	3.92262E-12	other
Serp1	9.40948E-07	0.21176759	0.026344656	other
Cdc16	4.36669E-15	0.211160745	1.22258E-10	other
Rps13	2.17107E-10	0.211101499	6.07855E-06	other
Tfpi	9.7517E-15	0.210617677	2.73028E-10	other
2010107E04R	1.14563E-07	0.210528722	0.003207524	
Cox5a	3.92268E-07	0.210160041	0.010982732	enzyme
Tgfb1i1	3.53953E-11	0.209999866	9.90996E-07	transcription regulator
Susd6	1.08344E-15	0.209622694	3.03343E-11	other
Ndufab1	1.48924E-09	0.20955857	4.16957E-05	enzyme
Mnda	1.70279E-10	0.209537592	4.76747E-06	other
Fgfr1	8.28759E-11	0.209430434	2.32036E-06	kinase
Rcn1	8.94215E-08	0.209344148	0.002503623	other
Rpl36	1.53059E-10	0.208732065	4.28534E-06	other
Fmo1	1.95337E-10	0.208459086	5.46904E-06	enzyme
Itgb1	5.87003E-11	0.208400176	1.64349E-06	transmembrane receptor
Pcbp2	6.33252E-08	0.207707612	0.001772978	other
Ndufc2	6.14759E-09	0.207370719	0.00017212	enzyme
Pvr	1.78525E-15	0.207035748	4.99833E-11	other
Kdelr1	1.0494E-07	0.206586017	0.002938117	transporter
Clta	1.08161E-09	0.206551244	3.02828E-05	other
Rps15	5.38329E-07	0.206295937	0.015072149	other
Hbp1	3.58677E-15	0.206123028	1.00422E-10	transcription regulator
Dusp10	1.68988E-09	0.205383457	4.73134E-05	phosphatase
Fkbp1a	1.57038E-07	0.205167611	0.004396759	enzyme
Coro1b	1.22587E-12	0.205089689	3.4322E-08	other
Cct4	1.29872E-09	0.204647641	3.63616E-05	other
Scand1	1.10364E-10	0.204240281	3.08996E-06	transcription regulator
S100a1	2.17724E-08	0.203796135	0.000609583	other
Lrrn4cl	1.19931E-06	0.203754794	0.033578154	other
Akr1b3	2.4974E-09	0.202855815	6.99222E-05	
Supt4a	1.53574E-11	0.202699298	4.29977E-07	
Pddc1	8.28771E-13	0.202344132	2.32039E-08	
Ero1l	5.30117E-15	0.202228786	1.48422E-10	
Mgst3	2.96737E-11	0.202225962	8.30806E-07	enzyme
Psma5	1.51003E-09	0.201823566	4.22778E-05	peptidase
Picalm	3.6221E-15	0.201654417	1.01412E-10	other
Ube2l6	3.00233E-13	0.201357444	8.40591E-09	enzyme
Rps24	2.35321E-09	0.200762227	6.58853E-05	other
Bnip3l	4.70956E-13	0.200413999	1.31858E-08	other
Smc4	5.4225E-10	0.199798751	1.51819E-05	transporter

Vdac1	6.17741E-09	0.19880091	0.000172955	ion channel
Gprc5b	3.84828E-15	0.198540031	1.07744E-10	G-protein coupled receptor
Limd2	1.06836E-13	0.197936418	2.9912E-09	other
Lbh	2.24312E-16	0.197225757	6.28029E-12	transcription regulator
Hacd4	3.69587E-15	0.197088171	1.03477E-10	enzyme
Naca	7.3148E-10	0.19647321	2.048E-05	transcription regulator
Zeb1	6.13954E-13	0.196342739	1.71895E-08	transcription regulator
Skap2	3.67974E-18	0.195760108	1.03025E-13	other
Sp110	1.4721E-11	0.195757516	4.12159E-07	transcription regulator
Lhfpl2	6.76471E-15	0.195620353	1.89398E-10	enzyme
Tspan3	6.47881E-10	0.195496256	1.81394E-05	other
Sh3gl1	1.76805E-11	0.195487302	4.9502E-07	other
Tmem9	3.89632E-14	0.19513377	1.09089E-09	other
Gusb	2.45124E-15	0.194941545	6.86298E-11	enzyme
Atp5c1	9.32883E-08	0.194805192	0.002611886	
Gars	1.68744E-08	0.193159442	0.000472449	enzyme
Iigp1	1.54446E-11	0.192390435	4.32419E-07	
Fermt2	1.33499E-07	0.191371502	0.003737702	other
Lama4	1.25193E-10	0.191297839	3.50514E-06	enzyme
Oaz2	3.44134E-12	0.190619826	9.63506E-08	other
Mfhas1	3.06096E-12	0.190421362	8.57008E-08	other
Dtnbp1	9.35697E-09	0.190197403	0.000261977	other
Fosl1	1.83013E-09	0.188636737	5.124E-05	transcription regulator
Sys1	2.41596E-11	0.187976622	6.76422E-07	other
Rbpms	8.30275E-15	0.187511711	2.3246E-10	transcription regulator
Elof1	5.45117E-12	0.187482111	1.52622E-07	other
Rgcc	3.50662E-08	0.187429427	0.000981782	other
Wdr1	9.31577E-10	0.187124762	2.60823E-05	other
Yaf2	2.63219E-16	0.186093404	7.3696E-12	transcription regulator
Xaf1	2.75936E-10	0.185923307	7.72566E-06	other
Plpp5	6.16433E-13	0.185480999	1.72589E-08	phosphatase
Prcp	3.93907E-15	0.184355226	1.10286E-10	peptidase
Anpep	2.52094E-07	0.184046071	0.007058138	peptidase
Dnpep	1.41282E-12	0.183822941	3.95562E-08	peptidase
Irak3	7.90303E-10	0.183777104	2.21269E-05	kinase
Ppp1r2	5.52155E-08	0.183406749	0.001545923	phosphatase
Tma7	4.85918E-08	0.182865614	0.001360473	other
Xpnpep1	3.42285E-12	0.182324362	9.58328E-08	peptidase
Pmepa1	2.51735E-10	0.182220365	7.04807E-06	other
Pmm1	3.58305E-13	0.181461092	1.00318E-08	enzyme
Rpl39	1.16384E-07	0.181274412	0.003258509	other
Strap	1.24112E-10	0.181150419	3.47489E-06	other
Elk3	1.75871E-15	0.180809521	4.92405E-11	transcription regulator
Glmp	2.46265E-12	0.180736884	6.89493E-08	transcription regulator
Ndufa10	1.08264E-10	0.180588819	3.03119E-06	transporter
Snx5	1.5765E-13	0.180584705	4.41387E-09	transporter
Myl12b	3.00494E-08	0.180571812	0.000841324	other
Fam92a	6.42746E-14	0.180436843	1.79956E-09	other
Myadm	7.07841E-08	0.180111856	0.001981814	other

Bfar	8.91287E-14	0.179776404	2.49543E-09	enzyme
Slc16a2	2.62423E-15	0.179212611	7.34732E-11	transporter
Epb41l3	5.60756E-17	0.178757332	1.57001E-12	other
Akap13	1.47988E-09	0.178705321	4.14336E-05	other
Galk1	5.47043E-11	0.177855655	1.53161E-06	kinase
Tmem59	2.06071E-07	0.1777504	0.005769586	peptidase
Panx1	8.11477E-14	0.177601817	2.27197E-09	transporter
Got1	1.26553E-14	0.177580025	3.54322E-10	enzyme
Gltscr2	2.9469E-10	0.177239942	8.25074E-06	
0610012G03F	8.51853E-11	0.177210735	2.38502E-06	
Cdc42ep2	3.31522E-10	0.176665222	9.28196E-06	other
Mvb12a	6.88244E-11	0.176458101	1.92695E-06	other
Osmr	1.81658E-12	0.176392068	5.08606E-08	transmembrane receptor
Dram1	8.11658E-15	0.176320943	2.27248E-10	other
Ube2a	1.88904E-12	0.176153484	5.28893E-08	enzyme
Fads3	5.47203E-15	0.176091937	1.53206E-10	enzyme
Cct5	1.9245E-07	0.175927538	0.005388226	other
Psma4	1.01871E-06	0.175612868	0.028521776	peptidase
Arl5a	2.40488E-13	0.175590069	6.73317E-09	enzyme
Mbnl2	1.10556E-11	0.175349406	3.09534E-07	other
Zfp330	4.64413E-15	0.17465495	1.30026E-10	
Crtap	2.53589E-10	0.174588946	7.09998E-06	other
Gmfb	3.47474E-11	0.174034005	9.72859E-07	growth factor
Galnt16	1.97965E-13	0.173854963	5.54264E-09	enzyme
Furin	9.18444E-12	0.173830064	2.57146E-07	peptidase
Psma1	2.00494E-08	0.17352241	0.000561343	peptidase
Mast4	3.43218E-14	0.173200644	9.60943E-10	kinase
Cox4i1	2.85531E-11	0.173121842	7.99429E-07	enzyme
Nudt21	7.63039E-13	0.173051341	2.13636E-08	other
Tor1aip2	7.6814E-12	0.172887258	2.15064E-07	other
Psmd7	1.59821E-07	0.172650658	0.004474663	other
Snx3	2.12017E-07	0.172487373	0.005936055	transporter
Mlec	2.08832E-10	0.172250025	5.84688E-06	other
Ube2i	1.33566E-08	0.17207293	0.000373959	enzyme
Ostf1	4.57525E-07	0.171706218	0.012809779	transcription regulator
2310009B15R	1.13936E-12	0.170814135	3.18998E-08	
Mapk6	4.09287E-10	0.170732467	1.14592E-05	kinase
Med10	7.77758E-11	0.170155585	2.17757E-06	other
Stard3nl	2.53577E-11	0.16984221	7.09964E-07	other
Dok1	3.54803E-13	0.169702298	9.93377E-09	kinase
Gm8730	9.83912E-08	0.168992705	0.002754757	
Rnaseh2c	1.73075E-07	0.168969263	0.00484575	other
Sgcb	1.35401E-14	0.168622805	3.79095E-10	other
Nnmt	3.11359E-13	0.168510562	8.71743E-09	enzyme
Bloc1s2	3.66934E-12	0.168040105	1.02734E-07	other
Stat1	9.2854E-13	0.167919696	2.59973E-08	transcription regulator
Vps29	4.13667E-09	0.167904321	0.000115819	transporter
Chp1	3.29769E-11	0.16787472	9.23286E-07	other
Egfr	2.065E-10	0.167826119	5.78157E-06	kinase

Ube2b	9.35961E-09	0.167662098	0.00026205	enzyme
Eri3	1.42492E-10	0.16744733	3.9895E-06	other
Pfdn1	3.52491E-10	0.167416063	9.86903E-06	other
Agap1	3.16022E-12	0.16732485	8.84798E-08	enzyme
Rpl21	1.64049E-06	0.166920817	0.045930487	other
Rpl35a	2.02204E-07	0.166513845	0.005661298	other
Gngt2	7.09762E-09	0.165865866	0.000198719	enzyme
Ncstn	7.59789E-13	0.165848236	2.12726E-08	peptidase
Colgalt1	7.46741E-08	0.1653435	0.002090725	enzyme
Ufm1	2.36585E-10	0.165336868	6.62391E-06	other
Poldip2	4.25924E-11	0.165308371	1.1925E-06	other
Sdcbp	1.12744E-06	0.164957387	0.031565983	enzyme
Mpdu1	3.49111E-14	0.164687914	9.77442E-10	other
Vcl	1.70255E-13	0.164626514	4.7668E-09	enzyme
Cmah	1.61049E-08	0.163909775	0.000450905	
Car13	1.51353E-14	0.163757765	4.23759E-10	
Pdcd6	1.99187E-07	0.163641783	0.005576827	other
Tcea3	1.49392E-10	0.163449437	4.18267E-06	transcription regulator
Adam9	2.01945E-12	0.163385635	5.65405E-08	peptidase
Arid5b	1.93388E-12	0.163242612	5.41447E-08	transcription regulator
Arsb	3.80348E-11	0.162745398	1.0649E-06	enzyme
Tmbim4	2.30663E-09	0.162453101	6.45812E-05	other
Dctn3	1.10525E-06	0.16236498	0.030944728	other
Mpp6	2.27982E-14	0.162278289	6.38304E-10	kinase
Eci2	8.47059E-12	0.162150531	2.3716E-07	enzyme
Gm9493	1.41661E-06	0.161571314	0.039662287	
Baiap2	1.92567E-16	0.161273627	5.39149E-12	kinase
Csnk2b	1.05182E-09	0.160564653	2.94489E-05	kinase
Cyb5r4	7.7858E-15	0.160253805	2.17987E-10	enzyme
Wwtr1	4.88522E-10	0.159773172	1.36776E-05	transcription regulator
Pbxip1	8.4092E-11	0.15971652	2.35441E-06	transcription regulator
Rala	3.85039E-10	0.159532952	1.07803E-05	enzyme
Gemin7	9.49406E-11	0.159504927	2.65815E-06	other
Mthfd2l	6.0372E-13	0.159370419	1.69029E-08	enzyme
Bak1	6.34516E-14	0.159339765	1.77652E-09	other
Rnf144a	7.52353E-12	0.159260855	2.10644E-07	enzyme
Acot7	3.58833E-13	0.159160835	1.00466E-08	enzyme
Emilin1	5.22966E-09	0.159082056	0.00014642	other
Itgav	2.99767E-11	0.158989478	8.39289E-07	transmembrane receptor
Azin2	3.70226E-14	0.158837411	1.03656E-09	enzyme
Adamts4	3.50537E-09	0.158559401	9.81434E-05	peptidase
Hist3h2a	1.1985E-11	0.157615957	3.35557E-07	other
Aga	1.41495E-10	0.157411519	3.96158E-06	enzyme
Cops6	1.99377E-08	0.157166706	0.000558216	other
Ptpn2	2.0947E-10	0.157127634	5.86474E-06	phosphatase
Glrx5	5.48781E-10	0.156848966	1.53648E-05	other
Ilk	5.22288E-09	0.15651442	0.00014623	kinase
Pgm2	3.50739E-14	0.156323231	9.81999E-10	enzyme
Atp6v1b2	1.514E-09	0.156206677	4.23889E-05	transporter

Wipi1	2.13034E-09	0.156068764	5.96454E-05	other
Pon3	1.01296E-13	0.155572184	2.83609E-09	enzyme
Smyd2	1.62835E-13	0.155290065	4.55905E-09	enzyme
Ypel5	3.27388E-12	0.155287991	9.1662E-08	other
Pgd	7.01938E-12	0.154128794	1.96528E-07	enzyme
Odc1	3.37992E-07	0.152848828	0.009463089	enzyme
Klf7	5.91226E-09	0.152759825	0.000165531	transcription regulator
Hmg20b	2.58158E-10	0.152563011	7.22792E-06	transcription regulator
Pdlim4	1.51073E-08	0.152545233	0.000422974	other
Pdcd10	3.39814E-11	0.152465245	9.51411E-07	other
Glod4	2.34754E-12	0.152380781	6.57263E-08	enzyme
Timm10b	1.00598E-08	0.152212948	0.000281653	transporter
H6pd	7.26282E-11	0.15216172	2.03345E-06	enzyme
Nucb1	4.65865E-08	0.151962729	0.001304328	other
Mrc2	9.43427E-10	0.151728496	2.64141E-05	
Ube2v2	6.58456E-09	0.151579661	0.000184354	enzyme
Fam103a1	8.91644E-11	0.151528031	2.49643E-06	
Selenbp1	1.17316E-07	0.151328591	0.003284623	other
Apex1	3.2563E-11	0.151097057	9.117E-07	enzyme
Polr2m	8.0395E-13	0.14991825	2.2509E-08	other
Flnc	9.68364E-14	0.149782191	2.71123E-09	other
Tcp11l2	1.28418E-09	0.149276278	3.59546E-05	other
Txnl1	8.74562E-07	0.149218886	0.024485985	enzyme
Mrps15	2.94646E-13	0.149165807	8.2495E-09	other
Mrpl32	3.42795E-10	0.149068182	9.59758E-06	translation regulator
Acads	3.96134E-12	0.148974221	1.10909E-07	enzyme
Sugt1	5.67815E-09	0.148938009	0.000158977	other
Fam96b	8.75858E-09	0.148679893	0.000245223	
Ldlrad4	1.68716E-12	0.148484344	4.72372E-08	other
Gm6133	2.94086E-09	0.148415774	8.23382E-05	
Ggct	4.29465E-11	0.14837355	1.20242E-06	enzyme
Ccni	2.90003E-09	0.148299983	8.1195E-05	other
Skil	9.85369E-10	0.148252577	2.75884E-05	transcription regulator
Pigp	9.30457E-08	0.14815914	0.002605093	enzyme
Il10rb	3.11015E-09	0.147526559	8.70779E-05	transmembrane receptor
Srp9	2.41514E-07	0.147513427	0.00676192	other
Sptlc2	1.13606E-11	0.146846957	3.18074E-07	enzyme
Mthfd2	9.77763E-09	0.146796366	0.000273754	enzyme
Rnf213	3.53139E-09	0.146535327	9.88717E-05	
Prkcsh	6.40298E-10	0.146295954	1.79271E-05	enzyme
Nit2	2.45619E-10	0.146190316	6.87685E-06	enzyme
Tnip1	2.63538E-12	0.146146733	7.37853E-08	other
Txndc9	1.38633E-10	0.145842159	3.88146E-06	other
Taf10	1.36095E-07	0.145644961	0.003810389	transcription regulator
Il15ra	1.39259E-11	0.145226909	3.89896E-07	transmembrane receptor
Cs	1.73585E-09	0.14521636	4.86002E-05	enzyme
Copz1	2.63529E-09	0.144913899	7.37828E-05	transporter
Nav1	1.18332E-07	0.144734105	0.003313071	enzyme
Ebpl	1.14047E-10	0.144624885	3.19308E-06	enzyme

Cdc42ep5	1.06323E-07	0.144391726	0.002976841	other
Ube2r2	1.28646E-08	0.144348297	0.000360182	enzyme
Adh5	1.06035E-06	0.144089616	0.02968766	enzyme
Ptrhd1	1.04499E-11	0.143897266	2.92577E-07	other
Cd320	1.6112E-12	0.143770769	4.51103E-08	transmembrane receptor
Creg1	9.14946E-09	0.143717964	0.000256167	transcription regulator
Rab9	6.16708E-11	0.143641156	1.72666E-06	
Txndc12	1.80964E-09	0.14346863	5.06662E-05	enzyme
Cpt1a	2.22195E-14	0.142879705	6.22103E-10	enzyme
Thbs3	9.46964E-07	0.1422196	0.026513108	other
Dctn2	1.68596E-08	0.141928199	0.000472035	other
Ppp1cc	7.1525E-08	0.141843196	0.002002557	phosphatase
Emc6	3.0058E-09	0.141789799	8.41565E-05	other
Plscr3	7.70332E-13	0.141780746	2.15678E-08	enzyme
Mrps33	1.09897E-06	0.141706772	0.030768825	other
Pam	4.68767E-07	0.14067824	0.013124534	enzyme
Chd3	1.12539E-09	0.140553139	3.15086E-05	enzyme
Ext2	8.93297E-10	0.140319933	2.50105E-05	enzyme
Rps18-ps3	1.70122E-07	0.140303966	0.004763083	
Tcp1	9.74056E-08	0.140071335	0.002727163	other
Cask	9.57963E-13	0.139913713	2.6821E-08	kinase
Serhl	1.20734E-11	0.139766454	3.3803E-07	other
Oxa1l	6.44201E-11	0.139653043	1.80363E-06	enzyme
Igbp1	8.70203E-11	0.138840435	2.43639E-06	phosphatase
Rilpl2	1.16551E-11	0.138630266	3.26319E-07	other
Ppp1r18	4.16599E-09	0.138415218	0.000116639	other
Mrpl14	1.29573E-08	0.138093927	0.000362778	other
Map2k2	1.70282E-06	0.138084026	0.047675536	kinase
Ndufb5	2.05332E-07	0.137329502	0.005748884	enzyme
Pnrc2	2.471E-10	0.137238955	6.91831E-06	other
Sumo1	9.34903E-07	0.137210741	0.026175417	enzyme
Lpgat1	1.81973E-10	0.136859094	5.09487E-06	enzyme
Cisd3	1.73764E-10	0.136626453	4.86505E-06	other
Eln	1.88228E-07	0.136312703	0.005269998	other
Bet1	7.38785E-08	0.135998781	0.00206845	transporter
Nsg1	1.42619E-13	0.135650485	3.99304E-09	other
Rcbtb2	5.27237E-13	0.134928344	1.47616E-08	other
Ddx41	4.66299E-10	0.134862711	1.30554E-05	enzyme
Cfl2	1.24304E-07	0.134758748	0.00348025	other
Cacnb3	7.71664E-15	0.134656077	2.16051E-10	ion channel
Ndufs2	8.98357E-09	0.134526712	0.000251522	enzyme
Fam132a	4.17381E-11	0.134366811	1.16858E-06	
Fzd4	4.76523E-09	0.134294828	0.000133417	G-protein coupled receptor
Soat1	1.61571E-12	0.134190275	4.52365E-08	enzyme
Klhdc2	9.48935E-11	0.134183422	2.65683E-06	other
Phgdh	2.21347E-10	0.134107265	6.19728E-06	enzyme
Asns	3.46448E-10	0.134018592	9.69985E-06	enzyme
Tbc1d2b	4.27241E-12	0.133989841	1.19619E-07	other
Flot1	4.36246E-07	0.133862678	0.012214002	other

Rab31	5.30418E-11	0.133858969	1.48506E-06	enzyme
AW551984	2.7423E-10	0.133712725	7.6779E-06	
Srp68	1.41879E-09	0.133702523	3.97232E-05	other
Irf9	2.88799E-11	0.133692288	8.0858E-07	transcription regulator
Me1	1.97356E-12	0.133326101	5.52556E-08	enzyme
Sdhaf4	6.50215E-08	0.133040314	0.001820472	enzyme
Klhl13	1.04677E-07	0.132839064	0.002930745	other
Ly96	7.97103E-11	0.132636576	2.23173E-06	transmembrane receptor
Mplkip	2.73783E-10	0.132378664	7.66537E-06	other
Aig1	1.18398E-07	0.13225961	0.003314907	other
Adam17	2.57693E-08	0.132181826	0.00072149	peptidase
Vapb	1.71587E-08	0.132066238	0.000480411	other
Ptprs	1.99703E-13	0.131817389	5.59127E-09	phosphatase
Ecm1	1.50511E-06	0.13160825	0.042139944	transporter
Dazap1	3.08398E-09	0.131595278	8.63454E-05	other
H2-Q7	6.72826E-07	0.130980321	0.018837791	
Pcdh7	5.05141E-09	0.13086315	0.000141429	other
Ccng2	2.91858E-10	0.130670531	8.17145E-06	other
Zfand3	5.14941E-09	0.130632268	0.000144173	other
Cby1	6.70843E-11	0.130482718	1.87823E-06	other
Arhgap31	3.28975E-08	0.130407982	0.000921063	other
Lman2	2.55686E-07	0.130115235	0.007158702	transporter
Josd2	1.77304E-09	0.129870464	4.96417E-05	enzyme
Nqo2	2.95107E-12	0.129847511	8.26241E-08	enzyme
Pxn	3.62958E-12	0.129846731	1.01621E-07	other
Mocs2	1.64238E-07	0.129531411	0.004598326	enzyme
Gtf3a	1.81098E-09	0.129438887	5.07039E-05	transcription regulator
Commd3	3.10457E-07	0.129310283	0.008692174	other
St3gal2	5.39068E-10	0.129258022	1.50928E-05	enzyme
Gm10269	1.5089E-10	0.129223709	4.22463E-06	
Mfsd1	4.967E-10	0.12911491	1.39066E-05	transporter
Snap47	1.14716E-10	0.129110779	3.21182E-06	other
Fam114a1	1.37246E-08	0.129089484	0.00038426	other
Cbr1	3.63682E-11	0.129068322	1.01824E-06	enzyme
Cd276	1.00482E-12	0.128795423	2.81329E-08	other
Lmf2	6.29614E-12	0.128671213	1.76279E-07	other
Cmc2	4.44037E-15	0.128291437	1.24322E-10	other
Tusc3	1.3059E-09	0.128044258	3.65626E-05	transporter
Ifngr2	2.762E-10	0.128025409	7.73306E-06	transmembrane receptor
Rnf114	1.03116E-10	0.127963229	2.88704E-06	transcription regulator
1110059E24R	2.42852E-13	0.127935282	6.79938E-09	
Glrx	2.2546E-09	0.127709792	6.31244E-05	enzyme
Ndufa8	5.15686E-07	0.127689562	0.014438189	enzyme
Relb	2.55579E-08	0.127516188	0.000715571	transcription regulator
L3hypdh	5.50152E-12	0.127194005	1.54031E-07	enzyme
Isyna1	2.89618E-10	0.126962298	8.10872E-06	enzyme
Klf10	4.69566E-07	0.12685393	0.013146912	transcription regulator
Atp13a3	6.13738E-10	0.126668036	1.71834E-05	transporter
Nek7	1.08749E-09	0.126378764	3.04475E-05	kinase

Psmc6	3.9637E-07	0.125315355	0.011097581	peptidase
Plgrkt	1.31384E-08	0.125253073	0.000367848	other
Slmo2	2.23525E-08	0.125019334	0.000625824	
Lsm10	5.56569E-11	0.12481941	1.55828E-06	other
Tpgs1	1.41778E-10	0.124716941	3.9695E-06	enzyme
Smarca4	7.00924E-10	0.123928043	1.96245E-05	transcription regulator
Drg1	4.46352E-09	0.123761962	0.00012497	enzyme
Cdk5	1.00914E-10	0.12346834	2.82539E-06	kinase
Tctex1d2	2.28076E-11	0.123215341	6.38567E-07	other
Rin2	1.65065E-07	0.122994125	0.004621489	other
Paics	2.55074E-07	0.122951046	0.007141551	enzyme
Efhd2	3.62164E-11	0.122889425	1.01399E-06	other
Ndufs3	3.05553E-07	0.122461835	0.008554863	enzyme
Nt5c	2.70384E-08	0.12241231	0.000757021	phosphatase
Dusp19	5.27664E-13	0.122395861	1.47735E-08	phosphatase
Ap1s1	7.00213E-09	0.122346181	0.000196046	transporter
Zfp703	1.09177E-09	0.12223212	3.05674E-05	
Snrpa1	5.16826E-08	0.12220695	0.001447011	other
Rnf26	2.05222E-09	0.12220454	5.7458E-05	enzyme
Erlec1	5.74028E-07	0.122068988	0.016071648	other
Rnaseh2a	1.85857E-08	0.121844427	0.000520363	enzyme
H2afy	7.90958E-10	0.121578377	2.21452E-05	other
Gyg	3.26999E-10	0.121374885	9.15532E-06	
Tnfrsf23	1.75247E-08	0.121257507	0.000490658	
Fat1	1.20423E-09	0.12122579	3.3716E-05	other
Ctdnep1	6.14271E-09	0.121120119	0.000171984	phosphatase
Urod	2.90421E-10	0.12069695	8.1312E-06	enzyme
Zdhhc20	1.03092E-10	0.120524856	2.88637E-06	enzyme
Suclg1	1.95023E-08	0.120247849	0.000546025	enzyme
2210013O21F	2.3345E-08	0.12003215	0.000653612	
Sin3b	7.35794E-07	0.119602637	0.02060076	transcription regulator
Ssu72	5.31696E-07	0.119409326	0.014886437	phosphatase
Mrps24	5.57377E-07	0.119365136	0.015605432	other
Ywhag	1.65274E-07	0.119070227	0.004627338	other
Slc9a3r1	9.44397E-11	0.119016002	2.64412E-06	other
Smarce1	1.76069E-09	0.118948912	4.92959E-05	transcription regulator
Ube2z	1.08226E-10	0.118748884	3.03013E-06	enzyme
Ctps	2.06668E-09	0.118666507	5.78629E-05	
Rnaseh2b	1.2057E-10	0.118470394	3.37572E-06	enzyme
Smad1	6.42877E-08	0.118451149	0.001799927	transcription regulator
Ict1	1.38453E-09	0.118412523	3.87642E-05	
Amdhd2	5.95437E-09	0.118409741	0.000166711	enzyme
Stk24	1.6298E-09	0.118396576	4.56311E-05	kinase
Med30	1.96825E-08	0.118212406	0.000551071	transcription regulator
Mospd3	1.12474E-10	0.118059362	3.14906E-06	other
Tmem261	2.34129E-08	0.117870461	0.000655513	
Stra13	3.42553E-07	0.117760607	0.009590811	
Rabggtb	4.17768E-10	0.117641138	1.16967E-05	enzyme
Ghitm	1.09874E-08	0.117578359	0.000307624	other

Plekho2	1.74624E-08	0.117570463	0.000488911	other
Snx2	2.13957E-11	0.117439861	5.99037E-07	transporter
Ccnd2	8.81225E-07	0.117435785	0.024672549	other
Stip1	1.06373E-08	0.11688315	0.000297824	other
Got2	2.01651E-09	0.116861948	5.64582E-05	enzyme
Pigs	2.24185E-11	0.116816953	6.27673E-07	enzyme
Pip5k1a	2.81826E-09	0.116647101	7.89058E-05	kinase
Zfp655	7.49515E-08	0.116604191	0.002098493	
P2rx4	5.22964E-10	0.116414091	1.46419E-05	ion channel
Hmox2	3.73673E-09	0.116360845	0.000104621	enzyme
Lrrfip1	3.92142E-11	0.116230724	1.09792E-06	transcription regulator
Prrc1	1.82996E-07	0.11612034	0.005123535	other
Sp100	7.79495E-08	0.11587944	0.002182431	transcription regulator
Eif2b2	1.67853E-10	0.115766682	4.69956E-06	other
Casp8	1.4588E-13	0.115426224	4.08434E-09	peptidase
Psmd10	1.19686E-10	0.115005353	3.35097E-06	transcription regulator
AI413582	8.94669E-09	0.114575563	0.000250489	
Ccnh	1.16785E-10	0.11441804	3.26976E-06	transcription regulator
Cxx1b	2.02291E-07	0.114380828	0.00566374	
Csad	3.29039E-08	0.114347472	0.000921243	enzyme
Ahsa2	1.90977E-08	0.114051966	0.000534698	
Renbp	4.25602E-10	0.114018225	1.1916E-05	enzyme
1300002E11R	1.44216E-09	0.113999853	4.03775E-05	
Ptpn1	9.27176E-11	0.113875687	2.59591E-06	phosphatase
Adipor1	6.61282E-07	0.113220374	0.018514563	transmembrane receptor
Asna1	1.99953E-07	0.112993327	0.005598295	transporter
Myh9	9.23549E-09	0.112528488	0.000258575	enzyme
Higd2a	1.88534E-08	0.112410915	0.000527859	other
Mrps7	1.47291E-10	0.112163826	4.12385E-06	other
Pld3	2.10958E-08	0.111899459	0.000590639	enzyme
Sae1	1.07709E-09	0.111891615	3.01565E-05	enzyme
1110065P20R	2.11839E-10	0.111754428	5.93107E-06	
Ube2e2	1.83859E-10	0.111310669	5.14767E-06	enzyme
Mtx2	6.06918E-09	0.111189884	0.000169925	transporter
Abhd17a	6.59565E-08	0.110759439	0.001846649	enzyme
Cmc1	2.13292E-10	0.110618393	5.97175E-06	other
Gadd45gip1	2.7043E-08	0.110414724	0.00075715	other
Pitpna	5.08827E-07	0.110352625	0.014246145	transporter
Sucla2	4.06619E-08	0.110279544	0.001138453	enzyme
Mrpl51	2.56412E-07	0.110198044	0.007179012	other
Bzw2	2.47841E-09	0.110197724	6.93905E-05	translation regulator
Commd6	3.85626E-09	0.109609079	0.000107967	other
Hspb11	1.59565E-11	0.109429626	4.46751E-07	other
Ncam1	6.11224E-08	0.108936949	0.001711304	other
Ergic2	8.19193E-08	0.108905419	0.002293577	other
Zcrb1	1.17586E-07	0.108245406	0.003292165	other
Hs2st1	8.97682E-09	0.107762854	0.000251333	enzyme
Usp18	8.52849E-07	0.107638064	0.023878078	peptidase
Borcs8	1.26475E-09	0.107637052	3.54104E-05	other

Uba52	1.59412E-08	0.10735581	0.000446321	enzyme
Galk2	1.96854E-09	0.1072266	5.51153E-05	kinase
Dph3	1.01717E-07	0.107053027	0.002847877	other
Oser1	4.24644E-08	0.107049664	0.001188919	enzyme
Aph1a	6.69716E-07	0.106854222	0.018750718	peptidase
Cfap20	1.34594E-07	0.106761728	0.003768374	other
Spg21	2.63514E-09	0.106586143	7.37785E-05	enzyme
Gfer	3.55275E-08	0.106460178	0.000994699	enzyme
Rtfdc1	9.01733E-11	0.106290707	2.52467E-06	
Pea15a	4.30876E-10	0.105993825	1.20637E-05	
2300009A05R	1.05899E-07	0.105370218	0.002964949	
Cops5	1.72717E-07	0.105263773	0.004835718	transcription regulator
Ndfip1	2.57058E-09	0.104725946	7.19711E-05	other
B3glct	3.3638E-11	0.104409555	9.41797E-07	enzyme
Ubfd1	6.23296E-10	0.104400257	1.7451E-05	other
Edem2	1.73862E-10	0.104388074	4.8678E-06	enzyme
Slc25a39	2.38607E-09	0.10409888	6.68053E-05	other
Ppa2	5.57057E-09	0.103892763	0.000155965	enzyme
C1d	5.80248E-07	0.103735853	0.016245773	transcription regulator
Blmh	1.80763E-07	0.103532633	0.005060999	peptidase
Tgfb1	9.24219E-09	0.1034651	0.000258763	growth factor
Tgif1	6.4539E-09	0.103211342	0.000180696	transcription regulator
Sh3glb2	2.27782E-08	0.103061568	0.000637744	other
Aimp1	1.46703E-07	0.102959256	0.004107396	cytokine
Acadl	5.10759E-08	0.102605282	0.001430023	enzyme
Apip	4.31141E-09	0.102121608	0.000120711	enzyme
Txnrd1	2.19365E-07	0.102081423	0.006141792	enzyme
Lap3	2.68154E-10	0.101799451	7.50778E-06	peptidase
Gmppa	6.33786E-09	0.101322632	0.000177447	enzyme
Hint3	3.00908E-08	0.10129806	0.000842483	enzyme
Desi2	1.42621E-08	0.101123924	0.000399311	enzyme
Med21	3.07794E-09	0.100813786	8.61761E-05	transcription regulator
March5	1.48002E-08	0.100692236	0.000414376	enzyme
Mpg	2.40488E-10	0.100328341	6.73319E-06	enzyme
Snap23	2.33715E-07	0.100039225	0.006543554	transporter
Chic2	2.52388E-07	0.099878434	0.007066367	other
Tspan4	1.73033E-07	0.09987496	0.004844576	other
Rpa3	1.38987E-09	0.099794611	3.89135E-05	other
Ccnd3	2.13061E-07	0.099649368	0.005965292	kinase
Cacybp	4.48702E-07	0.099226762	0.012562755	other
Decr1	1.20814E-09	0.09920696	3.38255E-05	enzyme
Crlf2	1.27246E-08	0.099021577	0.000356264	transmembrane receptor
Cdk5rap3	1.00405E-06	0.099010692	0.028111384	other
Guk1	1.30206E-06	0.098886659	0.036455135	kinase
Tm2d1	2.86625E-07	0.098842377	0.00802492	G-protein coupled receptor
Prorsd1	6.069E-09	0.0987983	0.00016992	
Ube2f	7.08593E-07	0.098719437	0.01983919	enzyme
Nipa2	1.6285E-08	0.098523176	0.000455947	other
Gramd3	1.29024E-07	0.098096445	0.003612407	

Dhrs1	1.35269E-09	0.097883266	3.78727E-05	enzyme
Scamp2	1.53102E-06	0.09784304	0.042865406	other
Srp72	1.10315E-06	0.097670119	0.030886114	kinase
9530068E07R	4.49775E-07	0.097396347	0.012592799	
Wbp1	6.81327E-10	0.097035266	1.90758E-05	other
Nmt2	5.67585E-10	0.096491037	1.58913E-05	enzyme
Dpcd	2.12722E-08	0.096313355	0.000595578	other
Rbm43	2.52934E-09	0.096184046	7.08164E-05	other
Arhgap1	1.03296E-08	0.096004481	0.000289207	other
Impa1	3.90502E-09	0.095525966	0.000109333	phosphatase
Stx2	2.30087E-07	0.095320754	0.006441985	transporter
Cul7	5.59093E-07	0.095160214	0.015653489	enzyme
Snx4	2.74532E-07	0.095039164	0.007686359	transporter
Pam16	1.7733E-06	0.095031893	0.049648801	other
Zwint	1.26763E-08	0.094953701	0.00035491	other
Rhog	8.7789E-10	0.094571658	2.45792E-05	enzyme
Mafb	2.30656E-09	0.094449687	6.45791E-05	transcription regulator
Snx7	3.6579E-07	0.09407395	0.010241396	transporter
Tbc1d20	1.73863E-09	0.094045954	4.86782E-05	other
Ift43	1.04711E-06	0.093969639	0.029317079	other
Fopnl	1.7583E-08	0.093733161	0.000492288	other
Tprkb	8.36279E-07	0.093633688	0.023414128	other
Psmd13	2.57432E-08	0.093530863	0.000720758	peptidase
Naa50	2.37664E-07	0.093423166	0.006654112	enzyme
Fam198b	5.2305E-10	0.092766243	1.46444E-05	
Ubtd1	1.74459E-07	0.092722558	0.004884511	other
Fam136a	8.52086E-10	0.092488234	2.38567E-05	other
Mrps30	3.883E-08	0.092116267	0.001087162	enzyme
Acd	4.46994E-08	0.092023166	0.001251495	other
Nr2f6	9.99981E-09	0.091619164	0.000279975	ligand-dependent nuclear receptor
Tyms	8.63269E-07	0.091604143	0.024169802	enzyme
Ctnnbip1	2.24398E-11	0.091365485	6.28268E-07	other
Elp3	3.65986E-09	0.091340475	0.000102469	enzyme
Adss	3.22958E-09	0.09118709	9.04218E-05	enzyme
Kdelc2	4.34801E-08	0.090944764	0.001217355	
Ak6	2.22187E-07	0.090796553	0.00622079	kinase
Nus1	6.21436E-08	0.090636979	0.001739896	enzyme
Vps36	4.21116E-09	0.090613606	0.000117904	other
Jak2	4.24479E-08	0.090328887	0.001188456	kinase
Atp6v1h	5.28E-08	0.090037402	0.001478294	transporter
Wdr45	2.90496E-08	0.08970954	0.000813331	other
Htatip2	7.8327E-08	0.089464673	0.002193	transcription regulator
Dhrs4	1.43634E-10	0.089461148	4.02147E-06	enzyme
Cln5	3.35035E-08	0.089271568	0.000938032	other
Foxo3	1.55713E-08	0.089100013	0.000435966	transcription regulator
Polr2g	2.92596E-09	0.088900559	8.19209E-05	enzyme
Tatdn1	1.61061E-12	0.088824045	4.50938E-08	other
Mrpl21	8.81265E-07	0.088616011	0.024673653	other
Aldh9a1	1.07825E-09	0.088468562	3.01889E-05	enzyme

Faim	2.12982E-08	0.088173397	0.000596306	other
Phldb2	3.1943E-07	0.087944491	0.008943406	other
Nfkb2	7.45194E-08	0.087872914	0.002086394	transcription regulator
2210016F16R	7.75912E-08	0.087753176	0.002172399	
Unc93b1	4.00609E-07	0.087615698	0.011216245	other
Lamtor3	1.02743E-06	0.087575003	0.028766023	other
Glb1	6.5147E-08	0.087168821	0.001823986	enzyme
Scyl1	5.69391E-09	0.087089507	0.000159418	kinase
Gm5617	3.10997E-08	0.086996679	0.00087073	
Rabl6	9.29499E-10	0.086979275	2.60241E-05	other
Naa20	1.22696E-06	0.086648789	0.034352377	enzyme
Prkacb	2.01661E-07	0.086437095	0.005646112	kinase
Polb	2.05686E-07	0.086150941	0.005758808	enzyme
Mrpl18	1.18619E-06	0.085918334	0.033210935	translation regulator
Gmds	4.16752E-09	0.085798174	0.000116682	enzyme
Mrps6	8.77067E-08	0.085792806	0.002455612	other
Mageh1	4.3379E-07	0.0857285	0.012145261	other
Rrp7a	2.48811E-08	0.085600193	0.00069662	other
Fam173a	4.46756E-07	0.084310112	0.012508262	enzyme
Ethe1	5.83888E-08	0.082971313	0.001634771	enzyme
Lamtor1	1.01836E-06	0.082302918	0.028512075	other
Itsn1	6.33612E-07	0.082231701	0.01773986	other
Abhd8	3.67315E-08	0.082086655	0.001028409	enzyme
Slc25a20	4.30534E-09	0.082036362	0.000120541	transporter
Hn1l	2.00402E-09	0.081862867	5.61085E-05	
Hscb	3.43187E-07	0.081539073	0.00960856	other
Tmem55a	8.86786E-08	0.081484716	0.002482823	
Ergic1	1.10683E-07	0.081480464	0.003098914	other
Cnot6	1.42717E-07	0.081401748	0.003995787	enzyme
Med7	4.68717E-07	0.081401603	0.013123131	transcription regulator
Fnip1	4.19624E-08	0.081324716	0.001174865	other
Elovl5	4.29878E-09	0.081216808	0.000120357	enzyme
Cops2	1.0704E-07	0.081080474	0.002996905	other
Atf6b	6.1104E-07	0.081008881	0.017107908	transcription regulator
Agfg1	6.59955E-09	0.080709132	0.000184774	other
Rpl7a-ps5	5.10763E-08	0.080500878	0.001430033	
Lmf1	7.96197E-08	0.080420693	0.002229193	other
Flnb	4.27664E-07	0.080417175	0.01197375	other
Ubr5	1.05221E-08	0.080116241	0.000294598	enzyme
Mrps35	2.03737E-09	0.080047392	5.70423E-05	other
Tmem70	5.56912E-08	0.080032166	0.001559241	other
Atp6v1a	8.93282E-08	0.079879751	0.00250101	transporter
Mrpl48	2.66483E-07	0.079878539	0.007460985	other
BC004004	3.65128E-07	0.079606506	0.010222851	
Ube2h	4.52574E-07	0.07944792	0.012671163	enzyme
Lzts2	1.91697E-07	0.079078757	0.005367139	other
Ap1m1	5.08458E-08	0.078354624	0.00142358	transporter
Dcaf13	3.68823E-07	0.078191069	0.010326317	other
Fech	1.92299E-07	0.077897793	0.005383977	enzyme

1600012H06F	2.31428E-07	0.077020235	0.006479529	
Igsf3	1.7471E-08	0.07677888	0.000489153	other
Ufsp2	3.01978E-07	0.075802155	0.00845478	enzyme
Cttn	4.40431E-08	0.075454542	0.001233119	other
Dctn5	2.17057E-08	0.075444725	0.000607715	other
Rnf2	2.82586E-08	0.075443501	0.000791183	transcription regulator
Dars	3.61735E-09	0.075330158	0.000101279	enzyme
Anapc15	1.46204E-08	0.075129883	0.000409342	other
Med9	6.4542E-07	0.075044508	0.018070477	other
Pdzd11	3.22986E-07	0.074941726	0.009042969	other
Pabpc4	1.07612E-06	0.074818872	0.030129323	translation regulator
Papola	1.90266E-07	0.074266487	0.005327071	enzyme
Kxd1	1.68876E-07	0.074160551	0.0047282	other
Pts	1.69765E-07	0.073966265	0.004753085	enzyme
Mmadhc	1.61239E-06	0.073963189	0.045143571	other
Kdm5b	3.02883E-08	0.0739387	0.000848013	transcription regulator
Rbfa	2.78568E-08	0.073634774	0.000779936	other
Idh3b	1.64572E-07	0.073568268	0.004607697	enzyme
Prkra	4.99986E-09	0.073327322	0.000139986	other
Mtap	9.66694E-07	0.072937995	0.02706551	enzyme
Tmem106b	1.70563E-06	0.072850883	0.047754259	other
Mrps23	4.428E-07	0.072733534	0.012397507	other
Mrpl3	5.97483E-09	0.072553379	0.000167283	other
Fars2	2.60317E-09	0.07246377	7.28836E-05	enzyme
Nosip	6.73429E-08	0.072355137	0.001885466	other
Sp140	6.47202E-07	0.072344566	0.018120364	transcription regulator
Mapk9	2.01507E-08	0.072257561	0.000564179	kinase
Spin1	1.83067E-09	0.072185201	5.12551E-05	other
Mzt1	9.18411E-07	0.072069087	0.025713677	other
Ccdc90b	6.80858E-08	0.072014542	0.001906268	other
Cited2	1.60127E-06	0.071678673	0.044832496	transcription regulator
Asnsd1	6.74612E-09	0.071528389	0.000188878	enzyme
Reep4	1.1741E-06	0.071489136	0.032872369	other
Fam207a	2.08494E-08	0.071479131	0.000583743	other
Phax	2.57812E-07	0.07137486	0.007218232	other
Commd9	6.77237E-07	0.071266725	0.018961288	other
2810474O19F	9.74262E-07	0.071096097	0.027277388	
Spata6	5.30426E-07	0.071022327	0.014850857	other
Pigc	1.85121E-07	0.070935981	0.00518302	enzyme
Rnf166	1.52646E-06	0.070859777	0.042737877	other
Atg5	5.70337E-07	0.070852705	0.015968297	other
Ykt6	4.53799E-07	0.070835916	0.012705476	enzyme
Sphk1	8.16701E-07	0.070732257	0.022865989	kinase
Cept1	9.07496E-07	0.069985909	0.025408072	enzyme
Golga7	4.97085E-07	0.069839764	0.013917377	other
Gamt	4.49219E-07	0.069707938	0.012577242	enzyme
Ppil1	1.31668E-06	0.069472438	0.036864369	enzyme
Gm10020	1.05238E-06	0.069433852	0.029464494	
Seh1l	2.60839E-07	0.068850677	0.007302961	transporter

Ndufa9	1.56906E-07	0.0687576	0.004393047	enzyme
Unc50	2.63654E-08	0.068554721	0.000738178	other
Hmgcl	5.45141E-07	0.068012695	0.015262851	enzyme
Nmi	5.28619E-08	0.067931573	0.001480026	transcription regulator
Samm50	8.49347E-07	0.067573571	0.023780019	other
Rps6ka4	8.5332E-07	0.067524283	0.023891243	kinase
Camk2g	7.62072E-08	0.067276401	0.002133648	kinase
Ubr4	1.29153E-06	0.066696332	0.036160287	enzyme
Tpst1	3.46664E-07	0.06645525	0.009705885	enzyme
Bcas2	3.08878E-07	0.066334321	0.008647972	other
Cpne2	1.07773E-06	0.066257986	0.030174417	other
Zbed3	1.06591E-06	0.066052034	0.029843422	transcription regulator
Myof	9.66861E-07	0.066007927	0.027070171	other
Mrrf	1.67453E-06	0.065982627	0.04688358	other
9030624J02R	1.1471E-06	0.065843185	0.032116486	
Kctd5	3.22715E-08	0.065715154	0.000903536	other
Akt1s1	1.45531E-07	0.065421195	0.004074572	other
Mycbp	3.41777E-08	0.065164796	0.000956906	transcription regulator
Pex2	4.37187E-09	0.064991039	0.000122404	other
Sap30l	3.40509E-07	0.064890212	0.009533562	transcription regulator
Tmem29	1.5563E-06	0.064814774	0.043573421	
Pank2	2.93266E-07	0.064806551	0.008210871	kinase
Pgs1	5.12188E-08	0.064475051	0.001434023	enzyme
Tmem33	3.00033E-08	0.064253797	0.000840032	other
Rtca	4.48684E-08	0.064167372	0.001256227	enzyme
Kansl2	2.10169E-08	0.063565582	0.000588431	other
Npepl1	1.54979E-06	0.063398734	0.043390896	peptidase
Slc25a24	1.16336E-07	0.06328624	0.003257186	transporter
Zfp414	7.50841E-08	0.06321613	0.002102206	
Mrpl22	1.04934E-06	0.063034677	0.029379374	other
Kdelc1	5.82294E-07	0.062797057	0.016303081	
Dera	1.77383E-09	0.062618125	4.96638E-05	enzyme
Actr1b	4.79583E-07	0.062328486	0.013427373	other
Polr2a	4.8287E-07	0.061409216	0.013519407	enzyme
Capza1	4.74114E-07	0.061143814	0.01327424	other
Zak	9.52735E-07	0.061018002	0.026674687	
Naprt	1.13056E-07	0.06075721	0.003165344	enzyme
Mrps9	1.8656E-07	0.060715698	0.005223308	other
Cnep1r1	1.26104E-06	0.060634073	0.035306551	other
Mpst	4.79568E-07	0.060330275	0.013426946	enzyme
Gsdmd	4.54828E-09	0.060070644	0.000127343	other
Phf5a	4.5242E-07	0.059342644	0.012666859	other
Kars	7.91711E-07	0.059294521	0.022166324	enzyme
B3galnt1	4.06535E-07	0.059084259	0.01138218	enzyme
Rragc	1.05406E-07	0.058960037	0.002951166	enzyme
Dst	1.0286E-06	0.058799607	0.028798612	other
Cdk9	1.46425E-07	0.058648684	0.004099618	kinase
Slc30a7	9.64196E-07	0.058417293	0.026995558	transporter
Sesn3	3.19752E-07	0.058314052	0.008952411	other

Dcps	1.4267E-06	0.058052162	0.039944639	enzyme
Fxr1	1.66564E-07	0.057647691	0.004663451	other
Mapkapk2	1.39958E-07	0.056848613	0.00391855	kinase
Srd5a3	4.76635E-08	0.056501472	0.001334483	enzyme
Naglu	1.38381E-07	0.05576483	0.003874403	enzyme
Manbal	3.58061E-08	0.055652002	0.0010025	other
Qars	1.22936E-06	0.055619724	0.034419658	
Cnppd1	1.36592E-06	0.055549833	0.038242933	other
Anapc2	1.33304E-06	0.055431292	0.037322571	other
Rfc2	6.44559E-08	0.055186173	0.001804637	other
Aebp2	4.03268E-07	0.055083506	0.011290686	transcription regulator
Mrpl10	3.48316E-07	0.055012032	0.009752163	other
Amz2	6.72203E-08	0.054507057	0.001882033	other
Pmpca	4.22584E-07	0.054432016	0.011831515	peptidase
Mboat7	1.32058E-06	0.054389613	0.036973617	enzyme
Gas2l1	5.34662E-08	0.054230822	0.001496945	other
Adgra2	8.11824E-09	0.054041585	0.000227295	G-protein coupled receptor
Vps72	1.15207E-07	0.054030593	0.003225559	transcription regulator
Arl8b	3.20661E-07	0.053827997	0.00897786	enzyme
Tufm	1.60092E-06	0.053816474	0.044822589	translation regulator
Ube2g2	1.01554E-06	0.053746734	0.028433075	enzyme
Ruvbl1	1.557E-06	0.05372177	0.043592757	transcription regulator
Katna1	1.61469E-06	0.0531978	0.045207972	enzyme
Jam3	8.80499E-07	0.053186492	0.024652201	other
Rab8b	8.86233E-07	0.05313952	0.024812745	enzyme
Mrpl35	1.98715E-07	0.052607481	0.005563632	other
Grhpr	4.67385E-09	0.052338539	0.000130859	enzyme
Mthfsl	4.55933E-07	0.051284449	0.012765223	
Ripk3	4.68664E-09	0.051033915	0.000131216	kinase
Snx9	2.82407E-08	0.050746728	0.000790682	transporter
Cpsf3l	3.99816E-08	0.050744718	0.001119406	
Rnf115	2.00067E-07	0.050558601	0.005601476	enzyme
Bbip1	1.94802E-07	0.050110158	0.005454055	other
Zfp511	3.65964E-07	0.049963766	0.010246273	
Zfpl1	1.53868E-07	0.0498341	0.004307995	other
Nob1	1.30259E-06	0.049134343	0.036469871	enzyme
Lsm1	3.80553E-08	0.048666916	0.001065472	other
Afg3l1	7.00016E-07	0.048160936	0.019599041	
Golt1b	6.03152E-08	0.048014746	0.001688704	other
Mrpl38	1.03071E-06	0.047576503	0.028857839	other
Map2k3	1.60321E-07	0.047367416	0.004488674	kinase
Polr2h	1.8804E-07	0.046784102	0.00526473	enzyme
Med8	7.75775E-07	0.046125932	0.021720136	other
9430016H08R	7.44481E-07	0.045947259	0.020843977	
Prpsap1	2.7752E-07	0.045794996	0.007770009	other
Bin3	2.14709E-08	0.045501676	0.000601143	other
Slmap	5.36922E-07	0.045086981	0.015032754	other
Tsta3	5.0958E-07	0.04496284	0.014267212	enzyme
Azi2	1.03782E-06	0.044315692	0.029057004	other

Tmem251	6.45172E-07	0.044227903	0.018063524	other
Tbpl1	3.31939E-07	0.041987776	0.009293616	transcription regulator
Hadha	8.06344E-07	0.041406447	0.022576016	enzyme
Gde1	2.16951E-08	0.041322032	0.00060742	enzyme
Mrpl28	1.53009E-06	0.041040681	0.042839397	other
Snrnp48	4.51933E-07	0.040528749	0.012653211	other
1700021F05R	3.6035E-07	0.039929163	0.010089067	
Pfdn4	9.77146E-07	0.039488555	0.027358122	other
Gins4	1.10435E-06	0.039311752	0.030919563	other
Bcat2	2.98548E-07	0.039295465	0.008358743	enzyme
Nt5m	9.88527E-07	0.039124308	0.027676784	phosphatase
Man2b1	1.42388E-06	0.038613874	0.039865816	enzyme
Mrpl45	1.226E-06	0.038034469	0.034325506	other
Lta4h	3.77163E-07	0.037342807	0.010559815	enzyme
Ube2e3	9.0214E-07	0.037189271	0.025258122	enzyme
9130401M01F	1.25218E-06	0.036974474	0.035058529	
Ufd1l	6.7741E-07	0.036063201	0.018966124	
Stau1	1.71249E-06	0.035730554	0.047946298	transporter
Stam2	7.37721E-07	0.035381694	0.020654722	other
Timm44	3.64479E-07	0.034062629	0.010204682	transporter
Gtf2e2	1.63578E-06	0.033979594	0.045798658	transcription regulator
Stat3	1.55854E-06	0.033614653	0.043635893	transcription regulator
Tmem230	5.39666E-09	0.033580468	0.000151096	other
BC029722	1.39861E-06	0.033234405	0.039158232	
Fam234a	9.55651E-07	0.033063104	0.026756317	other
Cars	1.36898E-07	0.032172678	0.003832869	enzyme
Atp1a1	3.19688E-07	0.030823829	0.008950611	transporter
Chmp6	2.84652E-07	0.030500469	0.007969684	other
Ube2g1	1.46545E-06	0.030491598	0.041029759	enzyme
Slc25a51	6.01997E-07	0.029372829	0.01685472	other
Sf3b4	9.76596E-07	0.029348955	0.027342727	other
Alkbh3	7.32063E-07	0.027790178	0.020496313	enzyme
Gatad1	2.4135E-07	0.026536764	0.00675733	transcription regulator
Gnl1	2.89202E-07	0.026145332	0.008097065	other
Prosc	6.33181E-07	0.025241133	0.017727814	
Trip12	1.2677E-06	0.012154129	0.035493091	enzyme
Myg1	1.67241E-06	0.010925756	0.046824209	
Mfsd5	4.85481E-07	0.007631566	0.013592494	transporter
Tsnax	1.47499E-06	0.007621051	0.041296812	other
Pgm3	1.08902E-06	0.007222675	0.03049048	enzyme
Vrk3	8.88835E-07	0.005139794	0.024885594	kinase
Anxa2	2.01053E-07	-0.09172697	0.005629071	other
Angptl1	1.15578E-06	-0.15164936	0.032359632	other
Cryab	5.45191E-13	-0.17802977	1.52643E-08	other
mt-Co1	9.20278E-09	-0.18608225	0.000257659	
Selk	4.1104E-08	-0.20244475	0.00115083	
Junb	2.03281E-08	-0.22479339	0.000569146	transcription regulator
Nenf	8.91159E-09	-0.22715426	0.000249507	growth factor
Jun	4.39205E-08	-0.23320901	0.001229687	transcription regulator

Gstm1	1.67868E-09	-0.23968046	4.69998E-05	enzyme
Dpt	7.40332E-12	-0.25103067	2.07278E-07	other
Ddhd1	6.54186E-09	-0.25450165	0.000183159	enzyme
Hnrnpa2b1	1.28707E-08	-0.25643127	0.000360355	other
Ddah2	4.43171E-07	-0.26635431	0.012407889	enzyme
Cd248	2.60859E-08	-0.28558995	0.000730353	other
Mllt4	4.57012E-10	-0.28577759	1.27954E-05	
Metrnl	2.17643E-11	-0.28725718	6.09356E-07	other
Egr1	6.34223E-12	-0.28823148	1.7757E-07	transcription regulator
Pcyt2	1.65485E-06	-0.28878314	0.046332614	enzyme
Actb	2.32292E-08	-0.29196296	0.00065037	other
Cd81	2.22782E-14	-0.29672167	6.23746E-10	other
C1ra	5.59131E-09	-0.29964977	0.000156546	
Amy1	5.41838E-10	-0.30029601	1.51704E-05	
Trabd2b	8.3233E-08	-0.30066043	0.002330358	peptidase
Eif1	4.97553E-26	-0.30497825	1.39305E-21	translation regulator
Lrp1	2.87254E-08	-0.3083358	0.000804253	transmembrane receptor
Per1	2.5794E-09	-0.30900776	7.22181E-05	
Krt17	3.81584E-07	-0.31231827	0.010683585	other
Derl1	1.77852E-06	-0.31259229	0.049794985	other
Sdf4	1.07076E-06	-0.31284536	0.029979028	other
Tnrc6c	1.38511E-06	-0.31597864	0.038780204	other
Rabac1	1.39182E-12	-0.32311538	3.89682E-08	other
Ldb2	4.65492E-08	-0.32693064	0.001303284	transcription regulator
Chpt1	5.27076E-09	-0.32876807	0.000147571	enzyme
Plac9b	6.77855E-10	-0.32880497	1.89786E-05	
Mn1	9.81923E-09	-0.32916943	0.000274919	other
Irf2bp2	9.17834E-07	-0.33028661	0.025697515	transcription regulator
Rnase4	3.63951E-19	-0.33769683	1.01899E-14	enzyme
H2-D1	2.77304E-11	-0.3417452	7.76396E-07	
Hnrnpa3	1.58429E-08	-0.34502425	0.00044357	other
Nfib	5.92473E-09	-0.34731203	0.000165881	transcription regulator
Irx1	9.51381E-09	-0.34750413	0.000266368	transcription regulator
Srebf2	1.05394E-06	-0.35778702	0.029508147	transcription regulator
Irx2	6.84679E-13	-0.35929066	1.91696E-08	transcription regulator
Rassf1	7.75864E-07	-0.36143582	0.021722638	other
Man2b2	1.62309E-09	-0.3638372	4.54432E-05	enzyme
Lpar1	3.27441E-07	-0.36399636	0.009167691	G-protein coupled receptor
Sptbn1	3.42574E-08	-0.36650121	0.000959138	other
Krt1	1.28622E-13	-0.36704348	3.60116E-09	other
Sdc4	4.09692E-07	-0.36804045	0.01147056	other
Adk	1.87538E-07	-0.36881944	0.005250699	kinase
Cd9	1.26904E-11	-0.37130348	3.55306E-07	other
Ptgfr	8.78226E-09	-0.37171588	0.000245886	G-protein coupled receptor
Uqcc2	5.96913E-07	-0.37330322	0.016712372	other
Carhsp1	2.50159E-07	-0.37347522	0.007003951	transcription regulator
Aplp2	1.10354E-06	-0.37454066	0.030896973	other
Prpf4b	1.76962E-11	-0.37575079	4.95458E-07	kinase
Atp1a2	1.29881E-12	-0.37601719	3.6364E-08	transporter

Lsamp	1.39502E-20	-0.37716103	3.90578E-16	other
S100a14	4.69902E-14	-0.37791269	1.31563E-09	other
Icosl	9.29332E-19	-0.37802938	2.60194E-14	
Crip1	4.38602E-26	-0.37815489	1.228E-21	other
Adh1	6.99575E-14	-0.37884997	1.95867E-09	
Fdft1	2.4166E-07	-0.37895806	0.006766002	enzyme
Diaph1	4.57559E-08	-0.37948342	0.001281074	other
Luc7l2	7.78074E-08	-0.3799734	0.002178452	other
Cuedc1	2.95141E-07	-0.38021427	0.008263351	other
Rrbp1	4.02087E-10	-0.38039542	1.12576E-05	other
Foxo1	1.87232E-11	-0.38107601	5.24211E-07	transcription regulator
Fcgrt	2.09524E-13	-0.38241704	5.86626E-09	transmembrane receptor
Krtdap	1.3503E-20	-0.3828344	3.78057E-16	other
Mid1ip1	2.60467E-07	-0.38312387	0.007292561	other
Cgref1	1.1294E-06	-0.38397696	0.031620826	other
Dnajc3	6.55422E-07	-0.38929812	0.018350507	other
Dusp1	4.20098E-14	-0.38963534	1.17619E-09	phosphatase
Clip1	2.60719E-07	-0.39094141	0.007299624	other
Slco2b1	7.89036E-12	-0.39243353	2.20914E-07	transporter
Ier2	1.5318E-13	-0.39392697	4.28875E-09	transcription regulator
Cd55	8.8784E-10	-0.39397271	2.48577E-05	other
Glud1	4.95075E-07	-0.39521152	0.013861103	enzyme
Serpinf1	5.93401E-20	-0.39538187	1.6614E-15	other
Aldh2	5.8493E-11	-0.39561579	1.63769E-06	enzyme
Prrx2	4.13982E-10	-0.39691208	1.15907E-05	transcription regulator
B930036N10F	5.05489E-12	-0.40152357	1.41527E-07	
Ism1	2.64378E-27	-0.40181218	7.40205E-23	other
Mef2a	3.24742E-07	-0.40438432	0.009092136	transcription regulator
Cyb5a	2.30071E-23	-0.40537283	6.44153E-19	enzyme
Pnrc1	1.40428E-10	-0.40726182	3.93169E-06	other
Il6ra	3.92655E-12	-0.40917524	1.09935E-07	
Spcs2	3.83009E-15	-0.41061393	1.07235E-10	other
Csf1	2.43929E-09	-0.41152048	6.82951E-05	cytokine
Ckb	1.78959E-08	-0.41191858	0.000501049	kinase
Man1a	1.00598E-11	-0.41270158	2.81655E-07	
Nktr	1.16973E-09	-0.41316269	3.27501E-05	enzyme
Adamtsl5	6.53436E-11	-0.41350472	1.82949E-06	other
Reck	2.55043E-12	-0.41514801	7.14069E-08	other
Flt3l	6.54734E-17	-0.41576212	1.83312E-12	
Pbx1	3.8934E-10	-0.41624204	1.09007E-05	transcription regulator
Itgb5	3.35644E-09	-0.41748423	9.39735E-05	other
Emp3	6.98951E-22	-0.41796113	1.95692E-17	other
Xist	5.121E-12	-0.4190219	1.43378E-07	other
Prrx1	9.20949E-14	-0.41910363	2.57847E-09	transcription regulator
Kazald1	6.01168E-10	-0.42061646	1.68315E-05	other
Cpxm2	4.10029E-14	-0.42259722	1.148E-09	peptidase
Ablim1	2.80043E-11	-0.42375732	7.84066E-07	other
Ddx5	4.87724E-16	-0.42377549	1.36553E-11	enzyme
Tra2a	5.40637E-07	-0.42457461	0.015136766	other

Tuba1b	1.10684E-19	-0.42779941	3.09893E-15	other
Hspa5	1.60172E-15	-0.42788362	4.48449E-11	enzyme
Fkbp2	4.69634E-14	-0.42811311	1.31488E-09	enzyme
Rbm39	3.00215E-23	-0.43052999	8.40543E-19	transcription regulator
Spon2	1.15855E-20	-0.43533954	3.24371E-16	other
Cpq	2.18817E-11	-0.43568994	6.12644E-07	peptidase
Anxa8	3.71149E-17	-0.43719714	1.03914E-12	other
Ptrf	1.11053E-13	-0.43808807	3.10927E-09	
Ntn1	6.87611E-17	-0.44566629	1.92517E-12	growth factor
Ang	1.28103E-14	-0.44717541	3.58663E-10	enzyme
Il17ra	1.77829E-06	-0.44719573	0.049788516	transmembrane receptor
Pth1r	3.39874E-13	-0.448118	9.5158E-09	G-protein coupled receptor
Zfp36l2	3.17071E-09	-0.4539083	8.87734E-05	transcription regulator
Lepr	3.42736E-26	-0.45584636	9.59592E-22	transmembrane receptor
Ptma	8.00412E-27	-0.45808677	2.24099E-22	other
Akr1c14	5.71895E-11	-0.45890408	1.60119E-06	
Fbln1	5.99664E-17	-0.46036372	1.67894E-12	other
Il4ra	1.0271E-08	-0.46208595	0.000287566	
Wsb1	3.3956E-10	-0.46433153	9.50699E-06	other
Chst12	2.37371E-12	-0.46467267	6.64591E-08	enzyme
Bmp7	7.94004E-31	-0.46537383	2.22305E-26	growth factor
Idi1	1.28434E-12	-0.46545052	3.59588E-08	enzyme
Lum	8.64496E-27	-0.46847845	2.42042E-22	other
Lamp1	8.09732E-26	-0.46849992	2.26709E-21	other
Plekha5	4.86755E-14	-0.46899551	1.36282E-09	other
Hn1	1.40027E-12	-0.46979587	3.92046E-08	
Dpep1	4.46412E-20	-0.47042125	1.24986E-15	peptidase
Pdcd4	3.80721E-13	-0.470777	1.06594E-08	other
Gja1	2.75397E-12	-0.47195717	7.71055E-08	transporter
Efemp1	1.7318E-18	-0.47418172	4.84868E-14	enzyme
mt-Atp6	4.1636E-41	-0.47810246	1.16573E-36	
mt-Co2	2.67835E-37	-0.48203641	7.49885E-33	
Ldlr	1.98865E-18	-0.48285129	5.56781E-14	transporter
Atp1b3	9.40175E-10	-0.48375339	2.6323E-05	transporter
Adgrd1	3.3378E-11	-0.48639635	9.34517E-07	G-protein coupled receptor
Jund	2.08734E-38	-0.48983722	5.84414E-34	transcription regulator
Creb3l1	2.27379E-09	-0.49021698	6.36617E-05	transcription regulator
Igsf10	9.4729E-14	-0.49210109	2.65222E-09	other
mt-Co3	5.3964E-46	-0.49425879	1.51088E-41	
Srsf5	1.71764E-14	-0.49667405	4.80904E-10	other
Son	8.33762E-19	-0.49691701	2.33437E-14	other
Gm9844	3.67503E-17	-0.49792912	1.02894E-12	
Fam25c	1.53753E-20	-0.49860791	4.30476E-16	other
AY036118	1.1095E-14	-0.50053602	3.10638E-10	
Tmem132c	2.02841E-36	-0.50133795	5.67915E-32	other
Ahnak2	1.01337E-06	-0.50153355	0.028372298	other
Rsrp1	6.80621E-11	-0.501671	1.9056E-06	other
Nupr1	7.13243E-14	-0.50406983	1.99694E-09	transcription regulator
Ogn	6.92482E-24	-0.50459483	1.93881E-19	growth factor

Hspb8	3.16566E-07	-0.50935461	0.008863214	kinase
Irx5	4.58323E-21	-0.5129873	1.28321E-16	transcription regulator
Tgfb2	2.92088E-08	-0.51320213	0.000817787	growth factor
Ltbp4	5.74957E-23	-0.51700139	1.60976E-18	growth factor
Dapk1	2.28916E-15	-0.51747972	6.40918E-11	kinase
Bcl11b	3.32541E-36	-0.51838264	9.31047E-32	transcription regulator
Scara5	2.20374E-22	-0.52180128	6.17004E-18	transmembrane receptor
Manf	2.80049E-21	-0.52784934	7.8408E-17	other
Fus	9.63169E-22	-0.52942871	2.69668E-17	transcription regulator
Sfn	4.09932E-10	-0.53033759	1.14773E-05	other
Hnrnph1	5.15105E-16	-0.53120648	1.44219E-11	other
Atf4	2.41015E-16	-0.53200578	6.74793E-12	transcription regulator
Tmem50a	1.08075E-28	-0.53214732	3.02588E-24	other
Fxyd1	3.34833E-27	-0.53259358	9.37464E-23	ion channel
Ar	2.32767E-15	-0.53756628	6.51701E-11	ligand-dependent nuclear receptor
Rarres2	5.35059E-35	-0.54147096	1.49806E-30	transmembrane receptor
Add3	1.29707E-19	-0.54400904	3.63155E-15	other
Hpgd	4.92476E-21	-0.54436971	1.37883E-16	enzyme
Tmsb10	1.00479E-54	-0.54480495	2.81321E-50	other
Ddx3x	2.58797E-14	-0.54551959	7.24579E-10	enzyme
Tnxb	4.01354E-25	-0.54849992	1.12371E-20	other
Bmp4	6.53488E-21	-0.55384311	1.82964E-16	growth factor
H2-K1	3.65457E-32	-0.55785382	1.02321E-27	
Neat1	1.10101E-15	-0.56134177	3.08262E-11	other
Tppp3	1.53634E-31	-0.56436899	4.30144E-27	other
Slco2a1	7.18076E-23	-0.57080207	2.01047E-18	transporter
Ccl27a	2.95459E-13	-0.57145281	8.27226E-09	
Tmem47	1.84432E-22	-0.57785858	5.16373E-18	other
Ackr1	5.1683E-29	-0.58032838	1.44702E-24	G-protein coupled receptor
Ifi27	1.7727E-28	-0.58037351	4.96321E-24	other
Cdh13	1.37602E-35	-0.58329945	3.85259E-31	other
Plpp3	2.99044E-28	-0.58607179	8.37263E-24	phosphatase
Cebpb	1.36967E-17	-0.58644927	3.83479E-13	transcription regulator
mt-Nd5	3.51732E-24	-0.58842101	9.8478E-20	
Rabep1	7.22656E-21	-0.58994635	2.02329E-16	transporter
Crp	1.34694E-50	-0.59243361	3.77117E-46	other
Dmkn	1.38989E-16	-0.59675513	3.89141E-12	other
Mgst1	7.83849E-33	-0.6014425	2.19462E-28	enzyme
Klf9	1.03201E-21	-0.60201196	2.88943E-17	transcription regulator
Adamts2	7.24051E-18	-0.6100562	2.0272E-13	peptidase
Nrn1	1.52728E-45	-0.6107856	4.27609E-41	other
Clec11a	2.43414E-19	-0.61152568	6.81511E-15	growth factor
Perp	8.17684E-23	-0.61273644	2.28935E-18	other
Timp2	9.47036E-47	-0.61500576	2.65151E-42	other
Srsf2	1.00809E-23	-0.6169023	2.82246E-19	transcription regulator
Pi16	7.71218E-25	-0.62003562	2.15926E-20	other
Tnmd	1.36788E-16	-0.62150866	3.82978E-12	other
Mbnl1	4.54074E-17	-0.62277086	1.27132E-12	other
Mcl1	2.5731E-17	-0.62830525	7.20418E-13	transporter

Tuba1a	6.77659E-38	-0.62996254	1.89731E-33	other
Cyr61	1.25564E-14	-0.63319737	3.51553E-10	
Cyp26b1	8.97861E-18	-0.63359608	2.51383E-13	enzyme
Mmp27	2.66996E-28	-0.64036049	7.47536E-24	peptidase
Cdh4	1.41508E-69	-0.64063228	3.96194E-65	other
Tgm2	2.19036E-07	-0.64768175	0.006132577	enzyme
Serpina3c	1.33364E-39	-0.65505686	3.73392E-35	
Cldn10	1.99651E-13	-0.65700766	5.58984E-09	other
Htra1	2.25175E-34	-0.66333187	6.30445E-30	peptidase
Serping1	1.15341E-44	-0.66394565	3.2293E-40	other
Thy1	1.97256E-33	-0.6666021	5.52278E-29	other
Gm26532	1.75967E-33	-0.67805022	4.92673E-29	
Fdps	1.52951E-16	-0.68325555	4.28233E-12	enzyme
Atf3	5.9115E-29	-0.68849669	1.6551E-24	transcription regulator
Insig1	6.43875E-20	-0.69750512	1.80272E-15	other
Ahnak	4.03131E-49	-0.70045432	1.12869E-44	other
Aebp1	7.67138E-44	-0.70134422	2.14783E-39	peptidase
mt-Cytb	4.57143E-65	-0.70580481	1.27991E-60	
Twist1	7.08889E-26	-0.70592308	1.98475E-21	transcription regulator
Btg2	4.74716E-29	-0.71226531	1.32911E-24	transcription regulator
Oxtr	1.55688E-83	-0.71364571	4.35894E-79	G-protein coupled receptor
Sertad1	1.29716E-18	-0.7197658	3.6318E-14	transcription regulator
Ccdc80	7.61354E-36	-0.72996291	2.13164E-31	other
Osr2	2.12301E-18	-0.73414646	5.94401E-14	transcription regulator
Tgfbr2	1.52842E-29	-0.73996435	4.27928E-25	kinase
Ly6d	3.20372E-32	-0.75036111	8.96976E-28	other
Lgals3	1.41145E-48	-0.75258378	3.95178E-44	other
Ly6a	1.8494E-56	-0.75287444	5.17796E-52	
Lbp	2.54145E-16	-0.75314709	7.11555E-12	transporter
Krt10	3.6616E-36	-0.75492694	1.02518E-31	other
Ndufa4l2	9.43089E-31	-0.75610153	2.64046E-26	enzyme
Tmem158	2.70889E-14	-0.76538349	7.58434E-10	other
Mat2a	3.52189E-24	-0.7727732	9.86058E-20	enzyme
Fxyd3	4.32529E-48	-0.78005458	1.21099E-43	ion channel
Adam33	3.58967E-33	-0.78713458	1.00504E-28	peptidase
Krt14	4.47175E-29	-0.78989275	1.252E-24	other
Atf5	1.06778E-30	-0.79029555	2.98956E-26	transcription regulator
Prnp	6.88107E-46	-0.79070445	1.92656E-41	other
Nr4a1	3.59264E-14	-0.79452215	1.00587E-09	ligand-dependent nuclear receptor
mt-Nd4	7.80687E-77	-0.7958728	2.18577E-72	
Ppp1r14a	2.35356E-36	-0.80251498	6.5895E-32	phosphatase
Fos	1.53347E-39	-0.81589354	4.29342E-35	transcription regulator
Gpc4	5.1067E-33	-0.81765483	1.42977E-28	transmembrane receptor
Malat1	1.60953E-56	-0.81775788	4.50635E-52	other
Clec3b	5.51669E-55	-0.84297614	1.54456E-50	other
Sectm1a	4.40541E-39	-0.87708588	1.23343E-34	
Pltp	1.68687E-48	-0.88240134	4.7229E-44	enzyme
Cyp4b1	6.22483E-35	-0.88252163	1.74283E-30	enzyme
mt-Nd3	5.67367E-50	-0.89741534	1.58851E-45	

Apoe	1.49666E-62	-0.89938008	4.19034E-58	transporter
Penk	8.97934E-28	-0.90345028	2.51404E-23	other
Ly6c1	1.21972E-57	-0.90836539	3.41497E-53	
Pmp22	6.78539E-62	-0.90902036	1.89977E-57	other
Igf1	2.8896E-23	-0.91838983	8.09031E-19	growth factor
mt-Nd1	9.2419E-102	-0.9257902	2.58755E-97	
Aff3	3.18633E-78	-0.92962883	8.9211E-74	transcription regulator
Igfbp6	3.02271E-65	-0.94560776	8.46298E-61	other
Crip2	6.47218E-61	-0.96820696	1.81208E-56	other
Cpz	1.8793E-27	-0.97154051	5.26167E-23	peptidase
Cilp	3.2631E-18	-0.97761695	9.13603E-14	phosphatase
Scd2	3.1255E-37	-0.99251061	8.75077E-33	
Ccl19	2.43496E-44	-0.99336148	6.81741E-40	cytokine
Gm42418	2.99579E-22	-1.01415419	8.38761E-18	
Krt5	1.61482E-49	-1.01441751	4.52118E-45	other
Klf4	9.08019E-56	-1.05667223	2.54227E-51	transcription regulator
Pik3r1	3.10829E-57	-1.06719789	8.70258E-53	kinase
Ecm2	6.9847E-58	-1.07941909	1.95558E-53	other
Cdkn1a	4.67827E-36	-1.11892284	1.30982E-31	kinase
Ghr	4.77184E-58	-1.11937422	1.33602E-53	transmembrane receptor
Cadm3	1.6644E-74	-1.15244203	4.65998E-70	other
Igfbp5	2.04699E-51	-1.17466183	5.73115E-47	other
Ebf1	2.51962E-59	-1.17776282	7.05444E-55	transcription regulator
Adh7	6.60807E-75	-1.18695858	1.85013E-70	enzyme
Cd34	1.4015E-103	-1.18702997	3.92382E-99	other
Fosb	4.38792E-84	-1.1983619	1.22853E-79	transcription regulator
St3gal1	1.55739E-78	-1.21044363	4.36038E-74	enzyme
Dcn	1.67633E-97	-1.22231084	4.69338E-93	other
mt-Nd2	1.4351E-108	-1.24449188	4.018E-104	
Tgfbi	3.18262E-57	-1.26250506	8.9107E-53	other
Gsn	6.45086E-91	-1.27007728	1.80611E-86	other
Pcolce2	3.7032E-92	-1.28291685	1.03682E-87	other
Lgals7	2.4205E-120	-1.40480071	6.7769E-116	other
Il1r2	1.08064E-98	-1.48591472	3.02558E-94	transmembrane receptor
Krt15	4.87641E-76	-1.52821261	1.3653E-71	other
Timp3	7.9601E-120	-2.22858212	2.2287E-115	other
Cyp2f2	7.7436E-144	-2.24614599	2.1681E-139	
Gas6	1.437E-165	-2.41520933	4.0233E-161	growth factor

Supplementary table 6: GSEA results comparing cluster 3 between wounded and normal skin.

Top upregulated gene sets (FDR 1-val < 0.01)

NAME	ES	NES	NOM p-val	FDR q-val
HALLMARK_EPITHELIAL_MESENCHYMAL_TRANSITION	0.56182504	2.0768063	0	0.0012308
HALLMARK_INTERFERON_GAMMA_RESPONSE	0.5994271	1.9689211	0	0.0019959
HALLMARK_TNFA_SIGNALING_VIA_NFKB	0.5586421	1.983369	0	0.0023539
HALLMARK_INFLAMMATORY_RESPONSE	0.5917196	1.8887726	0	0.0049408
HALLMARK_IL6_JAK_STAT3_SIGNALING	0.6400612	1.8502051	0	0.0075623
GOBP_MYELOID_LEUKOCYTE_MIGRATION	0.72665274	2.2326467	0	0
GOBP_GRANULOCYTE_CHEMOTAXIS	0.74480754	2.1184185	0	0.0010206
GOBP_INFLAMMATORY_RESPONSE	0.5811849	2.2033746	0	0.001034
GOBP_NEUTROPHIL_MIGRATION	0.7680535	2.1184337	0	0.0011482
GOBP_CELLULAR_RESPONSE_TO_BIOTIC_STIMULUS	0.71577317	2.1246574	0	0.0011881
GOBP_GRANULOCYTE_MIGRATION	0.73700255	2.1283505	0	0.0012231
GOBP_HUMORAL_IMMUNE_RESPONSE	0.7519506	2.1398494	0	0.0012801
GOBP_NEUTROPHIL_CHEMOTAXIS	0.7768922	2.1205394	0	0.0013122
GOBP_CELLULAR_RESPONSE_TO_MOLECULE_OF_BACTERIA	0.739521	2.172188	0	0.0013696
GOBP_LEUKOCYTE_CHEMOTAXIS	0.6848653	2.0926037	0	0.0016407
GOBP_NUCLEAR_TRANSCRIBED_MRNA_CATABOLIC_PROCE	0.6050548	2.0801327	0	0.0018635
GOBP_CELL_CHEMOTAXIS	0.623618	2.0653222	0	0.0025583
GOBP_RESPONSE_TO_CHEMOKINE	0.79396045	2.0187645	0	0.0056148
GOBP_LEUKOCYTE_MIGRATION	0.5877587	1.9988539	0	0.0070669
GOBP_VIRAL_GENE_EXPRESSION	0.5780752	2.0031614	0	0.0071337
GOBP_COTRANSLATIONAL_PROTEIN_TARGETING_TO_MEMB	0.5902549	1.98632	0	0.0080485
GOBP_DEFENSE_RESPONSE	0.4945026	1.9793266	0	0.0089086

EMT genes

NAME	Rank in query	Rank score	Running ES	Core
CXCL1	1	3.63117409	0.04846093	Yes
CTHRC1	4	3.04780006	0.08838697	Yes
PTX3	5	2.98075342	0.12869745	Yes
TIMP1	8	2.53677511	0.1617126	Yes
IGFBP4	9	2.53238916	0.19595958	Yes
MGP	12	2.14902306	0.22373094	Yes
POSTN	16	1.87886024	0.24720313	Yes
SPP1	17	1.86461079	0.27241936	Yes
TNC	18	1.73721921	0.2959128	Yes
ACTA2	19	1.68314481	0.31867495	Yes
CD44	25	1.38404036	0.33416426	Yes
SERPINE1	29	1.22711074	0.34882247	Yes
IL6	32	1.18000233	0.36348918	Yes
THBS1	33	1.17702603	0.37940678	Yes
MFAP5	34	1.16609764	0.39517662	Yes
SFRP4	37	1.12981308	0.40916458	Yes
MMP3	41	1.08256698	0.42186806	Yes
TPM2	42	1.08034027	0.4364781	Yes
BGN	56	0.93109429	0.44067734	Yes
GADD45A	66	0.86131853	0.44651526	Yes
LRRC15	71	0.84175414	0.45531648	Yes
TNFAIP3	76	0.8199051	0.46382225	Yes

EFEMP2	86	0.80021185	0.46883377	Yes
LOX	92	0.77327454	0.47606334	Yes
GADD45B	93	0.77011555	0.48647806	Yes
COL12A1	113	0.69640344	0.48362994	Yes
SERPINE2	122	0.65158373	0.4872771	Yes
COL6A3	127	0.63220531	0.49324447	Yes
GPX7	133	0.61474454	0.49833012	Yes
COL5A3	139	0.60483336	0.5032818	Yes
TPM1	140	0.59758252	0.5113632	Yes
VCAM1	144	0.58733535	0.51736933	Yes
COL6A2	145	0.58723205	0.5253108	Yes
CALD1	155	0.56641245	0.5271606	Yes
COL5A1	161	0.55596668	0.53145134	Yes
TPM4	173	0.52269572	0.5314187	Yes
LGALS1	174	0.51864308	0.5384326	Yes
FSTL1	184	0.49690923	0.5393424	Yes
COL4A2	193	0.48707995	0.54076487	Yes
BASP1	195	0.48504934	0.5466789	Yes
COL5A2	204	0.47416076	0.5479266	Yes
COL3A1	208	0.47260454	0.5523812	Yes
DPYSL3	215	0.46341342	0.55477476	Yes
ABI3BP	219	0.45484826	0.5589892	Yes
PLOD2	242	0.42119092	0.5504825	Yes
SDC1	243	0.42014208	0.5561643	Yes
FBLN2	244	0.41858038	0.56182504	Yes
ADAM12	260	0.40583894	0.55762976	No
SPARC	276	0.38533622	0.5531572	No
LOXL2	286	0.37555921	0.5524259	No
VIM	290	0.37158212	0.55551434	No
COL4A1	306	0.36084545	0.5507106	No
MYL9	314	0.35739955	0.55102485	No
GEM	315	0.35727337	0.55585647	No
FAP	320	0.353773	0.55805844	No
LOXL1	327	0.3479133	0.55889004	No
FBN2	336	0.34023422	0.5583266	No
VCAN	380	0.31426072	0.5348167	No
NID2	388	0.30843794	0.5344688	No
MATN2	429	0.28577107	0.51251036	No
TFPI2	446	0.28064221	0.5059764	No
BMP1	481	0.26230735	0.4875741	No
P3H1	482	0.26183712	0.49111506	No
LAMC1	546	0.2286935	0.45353642	No
PLOD3	552	0.22624162	0.45336813	No
PLOD1	554	0.22562626	0.45577383	No
PLAUR	564	0.22222644	0.45296893	No
ITGA5	573	0.21879466	0.4507632	No
ITGB1	606	0.20840017	0.43292305	No
PVR	609	0.20703575	0.43443173	No
FERMT2	649	0.1913715	0.41184223	No
ANPEP	664	0.18404607	0.4052931	No

PMEPA1	670	0.18222037	0.4045295	No
SGCB	723	0.16862281	0.37323985	No
NNMT	724	0.16851056	0.3755187	No
COLGALT1	738	0.16534349	0.36936224	No
ITGAV	768	0.15898947	0.3527906	No
PDLIM4	787	0.15254523	0.34323314	No
ELN	861	0.13631271	0.2979494	No
ECM1	890	0.13160825	0.28165305	No
TGFB1	1044	0.1034651	0.18427886	No
DST	1265	0.05879961	0.04304693	No
JUN	1366	-0.233209	-0.01835703	No
LRP1	1383	-0.3083358	-0.02451647	No
SDC4	1407	-0.3680404	-0.03438753	No
ITGB5	1456	-0.4174842	-0.05972938	No
EMP3	1457	-0.4179611	-0.05407704	No
PRRX1	1459	-0.4191036	-0.04905484	No
FBLN1	1481	-0.4603637	-0.0563862	No
LUM	1487	-0.4684784	-0.05327858	No
GJA1	1493	-0.4719572	-0.05012392	No
TGM2	1568	-0.6476818	-0.08913769	No
HTRA1	1571	-0.6633319	-0.08145822	No
THY1	1573	-0.6666021	-0.07308894	No
OXTR	1583	-0.7136457	-0.0692481	No
PMP22	1615	-0.9090204	-0.07696779	No
ECM2	1629	-1.0794191	-0.07076268	No
DCN	1639	-1.2223108	-0.06004285	No
TGFBI	1641	-1.2625051	-0.04361483	No
PCOLCE2	1643	-1.2829169	-0.02691077	No
TIMP3	1647	-2.2285821	0.00129093	No

Angiogenesis genes

NAME	Rank in query	Rank score	Running ES	Core
TIMP1	8	2.53677511	0.2117435	Yes
POSTN	16	1.87886024	0.36791298	Yes
SPP1	17	1.86461079	0.5271521	Yes
LPL	95	0.74945396	0.54400355	Yes
FSTL1	184	0.49690923	0.53255147	Yes
COL5A2	204	0.47416076	0.56141007	Yes
COL3A1	208	0.47260454	0.59993374	Yes
THBD	253	0.41304538	0.60826385	Yes
NRP1	303	0.36272347	0.60923463	Yes
APP	325	0.35006934	0.626271	Yes
VCAN	380	0.31426072	0.62004113	No
JAG1	463	0.2708903	0.5929611	No
FGFR1	602	0.20943044	0.52633953	No
ITGAV	768	0.15898947	0.4388763	No
CCND2	977	0.11743578	0.3215325	No
LUM	1487	-0.4684784	0.04984464	No
SLCO2A1	1539	-0.5708021	0.06736069	No

Supplementary table 7: DEGs between fibroblast clusters in wound and normal skin

Highlighted in red: upregulated genes

Highlighted in green: downregulated genes

Top DEGs upregulated in > 1 fibroblast cluster in wound with no downregulation.

Gene_name	Cluster 0	Cluster 3	Cluster 9	Cluster 14	Cluster 17	Up	Down
Cxcl1	0.97	3.63	1.26	1.18		4	0
Cxcl2	1.79	3.57	2.18	1.59		4	0
Ccl2	1.81	3.28	1.90	1.35		4	0
Timp1	0.91	2.54	1.77	1.65		4	0
S100a9	0.76	1.07	0.88	0.84		4	0
Il1b	0.82	0.99	0.96	0.90		4	0
H2-Ab1	0.68	0.80	0.73	0.92		4	0
Ppic	0.35	0.77	0.50	0.77		4	0
H2-Eb1	0.52	0.71	0.57	0.75		4	0
Eif3f	0.40	0.67	0.35	0.53		4	0

Top DEGs downregulated in > 1 fibroblast cluster in wound with no upregulation

Gene_name	Cluster 0	Cluster 3	Cluster 9	Cluster 14	Cluster 17	Up	Down
Lgals7	-1.20	-1.40	-1.50	-1.78	-0.57	0	5
Fxyd3	-0.62	-0.78	-0.59	-2.62		0	4
Fos	-0.38	-0.82	-0.74	-1.80		0	4
mt-Nd2	-0.51	-1.24	-0.38	-0.80		0	4
Krt15	-1.36	-1.53	-1.49	-1.39		0	4
Hnrnpa3	-0.37	-0.35	-0.50			0	3
Krt1	-0.45	-0.37	-0.58			0	3
S100a14	-0.44	-0.38	-0.58			0	3
Krtdap	-0.74	-0.38	-0.68			0	3
Anxa8	-0.52	-0.44	-0.34			0	3

Top DEGs found to be upregulated and downregulated in at least 1 fibroblast cluster.

Gene_name	Cluster 0	Cluster 3	Cluster 9	Cluster 14	Cluster 17	Up	Downregulated
Phlda1		1.67		-2.42		1	1
Mfap5	-0.69	1.17				1	1
Col5a3	-0.35	0.60	0.35			2	1
Sbsn	-0.27	0.59				1	1
Mt1	0.39	0.59		-1.43		2	1
Cilp	1.37	-0.98	0.68			2	1
Ccl19	0.35	-0.99				1	1
Igfbp5	-0.57	-1.17		1.71		1	2
Dcn		-1.22	0.38			1	1
Tgfbi		-1.26	0.55			1	1

Top DEGs upregulated in one fibroblast cluster.

Gene_name	Cluster 0	Cluster 3	Cluster 9	Cluster 14	Cluster 17	Up	Downregulated
Gas1	0.71					1	0
Gadd45g	0.69					1	0

Saa3		4.19				1	0
Cthrc1		3.05				1	0
Chchd10			1.19			1	0
Ctsl			0.76			1	0
Des				2.78		1	0
Mest				2.62		1	0
Mustn1					4.01	1	0

Top DEGs downregulated in one fibroblast cluster.

Gene_name	Cluster 0	Cluster 3	Cluster 9	Cluster 14	Cluster 17	Up	Down
Fn1	-0.59					0	1
Pi15	-0.57					0	1
Il1r2		-1.49				0	1
Adh7		-1.19				0	1
Igfbp3			-2.15			0	1
Crabp1			-2.14			0	1
Mbp				-4.30		0	1
Dct				-3.28		0	1
Tnni3k					-1.63	0	1
Mcc					-0.81	0	1

DEGs identified to be involved in EMT fibroblast clusters in wounded skin (log2FC ±0.25).

Gene_name	Cluster 0	Cluster 3	Cluster 9	Cluster 14	Cluster 17	Up	Down
Cxcl1	0.97	3.63	1.26	1.18		4	0
Cthrc1		3.05				1	0
Ptx3		2.98				1	0
Timp1	0.91	2.54	1.77	1.65		4	0
Igfbp4		2.53		1.06		2	0
Mgp		2.15	2.08	2.61		3	0
Postn		1.88	0.88			2	0
Spp1		1.86				1	0
Tnc		1.74	1.75			2	0
Acta2		1.68	0.90	3.22		3	0
Cd44		1.38	0.42			2	0
Serpine1	0.63	1.23	0.84			3	0
Il6	0.77	1.18				2	0
Thbs1	1.01	1.18	0.54			3	0
Mfap5	-0.69	1.17				1	1
Sfrp4		1.13				1	0
Mmp3	0.72	1.08	1.74			3	0
Tpm2		1.08				1	0
Bgn		0.93	0.69	2.16		3	0
Gadd45a		0.86		1.33		2	0
Lrrc15		0.84	0.82			2	0
Tnfaip3	0.78	0.82	0.38			3	0
Efemp2		0.80				1	0

Lox		0.77	0.83		2	0
Gadd45b	0.52	0.77			2	0
Col12a1		0.70	0.60		2	0
Serpine2		0.65			1	0
Col6a3	0.40	0.63	0.49		3	0
Gpx7		0.61	0.44	0.69	3	0
Col5a3	-0.35	0.60	0.35		2	1
Tpm1		0.60		1.09	2	0
Vcam1	0.39	0.59		1.45	3	0
Col6a2		0.59	0.31		2	0
Cald1		0.57		1.11	2	0
Col5a1	-0.28	0.56			1	1
Tpm4		0.52		1.10	2	0
Lgals1		0.52		1.33	2	0
Fstl1		0.50	0.58	0.84	3	0
Col4a2		0.49	0.30		2	0
Basp1		0.49			1	0
Col5a2		0.47	0.65	1.15	3	0
Col3a1		0.47	0.42		2	0
Dpysl3		0.46			1	0
Abi3bp		0.45	0.63		2	0
Plod2		0.42			1	0
Sdc1		0.42			1	0
Fbln2		0.42	0.77		2	0
Adam12		0.41			1	0
Sparc		0.39	0.42		2	0
Loxl2	-0.51	0.38			1	1
Vim		0.37			1	0
Col4a1		0.36			1	0
Myl9		0.36			1	0
Gem	0.44	0.36			2	0
Fap		0.35			1	0
Loxl1		0.35	0.49	1.45	3	0
Fbn2		0.34			1	0
Vcan	-0.27	0.31			1	1
Nid2		0.31			1	0
Matn2		0.29		0.68	2	0
Tfpi2		0.28			1	0
Bmp1		0.26			1	0
P3h1		0.26			1	0
Lrp1	0.26	-0.31	0.57		2	1
Sdc4		-0.37			0	1
Itgb5		-0.42			0	1
Emp3	-0.30	-0.42			0	2
Prrx1		-0.42			0	1
Fbln1	0.31	-0.46	0.54		2	1

Lum		-0.47			0	1
Gja1	0.29	-0.47			1	1
Tgm2		-0.65			0	1
Htra1		-0.66			0	1
Thy1	-0.33	-0.67			0	2
Oxtr	-0.64	-0.71			0	2
Pmp22	-0.31	-0.91		-3.07	0	3
Ecm2		-1.08			0	1
Dcn		-1.22	0.38		1	1
Tgfbi		-1.26	0.55		1	1
Pcolce2	-0.34	-1.28			0	2
Timp3	-0.90	-2.23			0	2
Pdlim4				2.60	1	0
Pmepa1			0.63	0.96	2	0
Colgalt1				0.49	1	0
Jun				-1.63	0	1
Eln	0.57		0.81		2	0
Nnmt	0.47		0.72		2	0
Anpep			0.26		1	0

Supplementary table 8: Similarity check between cluster 3 DEGs signature when comparing wounded and normal skin with drug signature using ConnectivityMap

Drugs/ compounds with p value < 0.05

Highlighted in red with positive enrichment scores.

Highlighted in green with negative enrichment scores.

cmap name	mean	n	enrichment	p value	specificity	percent non-null
TTNPB	0.69	2	0.96	0.00266	0	100
verteporfin	0.53	3	0.841	0.00787	0.0714	66
nicotinic acid	0.384	4	0.837	0.00109	0	75
hydrocotarnine	0.318	4	0.792	0.00364	0	50
iloprost	0.375	3	0.782	0.02109	0.0855	66
ticarcillin	0.293	3	0.757	0.0285	0.0199	66
cyclizine	0.366	4	0.712	0.014	0.0133	75
latamoxef	0.326	3	0.706	0.04984	0.058	66
etodolac	0.337	5	0.684	0.00809	0	80
cefapirin	0.239	4	0.677	0.02381	0.0231	50
selegiline	0.237	4	0.672	0.0252	0	50
chlorambucil	0.434	4	0.664	0.02896	0.0127	75
cefamandole	0.307	4	0.648	0.03654	0.1563	75
pyridoxine	0.429	4	0.646	0.0374	0.0648	75
7-aminocephalospo	0.362	4	0.645	0.03841	0.0265	75
pentoxyverine	0.365	4	0.634	0.04434	0.0957	75
enoxacin	0.477	4	0.626	0.04904	0.04	75
aminophenazone	0.202	5	0.611	0.02653	0.0211	60
furaltadone	0.392	6	0.585	0.01752	0.0216	66
glipizide	0.4	5	0.571	0.04588	0.028	60
fenoprofen	0.238	6	0.568	0.02398	0.0517	50
CP-690334-01	-0.326	8	-0.494	0.02479	0.1361	50
loperamide	-0.449	6	-0.54	0.03764	0.1842	66
guanadrel	-0.34	5	-0.593	0.03234	0.1522	60
etiocholanolone	-0.293	6	-0.625	0.00878	0.1688	50
lomustine	-0.29	4	-0.625	0.04852	0.2887	50
hydrastine hydrochl	-0.429	4	-0.629	0.04617	0.0629	75
leflunomide	-0.377	4	-0.632	0.04468	0.0963	50
iobenguane	-0.277	4	-0.637	0.04168	0.039	50
diphenylpyraline	-0.479	6	-0.639	0.00642	0.0188	83
co-dergocrine mesil	-0.425	4	-0.642	0.03917	0.0827	75
dihydroergocristine	-0.337	4	-0.646	0.0375	0.0615	50
tranylcypromine	-0.482	5	-0.658	0.01137	0.055	80
ethosuximide	-0.44	4	-0.658	0.03123	0.046	75
sulfathiazole	-0.408	5	-0.659	0.01101	0.0428	60
tiabendazole	-0.416	4	-0.661	0.0298	0.068	75
citalopram	-0.234	4	-0.664	0.02863	0.0208	50
quipazine	-0.218	4	-0.666	0.02823	0.0189	50
chlorphenesin	-0.332	4	-0.674	0.02522	0.0388	50
furazolidone	-0.234	4	-0.674	0.02536	0.051	50
methoxamine	-0.252	4	-0.679	0.02357	0.0839	50
moracizine	-0.463	4	-0.682	0.02226	0.0265	75
ondansetron	-0.368	4	-0.686	0.02103	0.0376	75

propidium iodide	-0.381	4	-0.701	0.01675	0	75
pepstatin	-0.478	4	-0.704	0.01581	0.006	75
vancomycin	-0.314	4	-0.706	0.0155	0.0621	50
acetohexamide	-0.358	4	-0.711	0.01414	0.0071	75
oxymetazoline	-0.381	4	-0.716	0.01329	0.0121	75
ethionamide	-0.376	3	-0.721	0.04469	0.0813	66
primidone	-0.211	4	-0.73	0.01062	0.0109	50
finasteride	-0.354	6	-0.741	0.00064	0.0244	50
alprenolol	-0.374	4	-0.765	0.00619	0	75
lansoprazole	-0.36	4	-0.771	0.00557	0.0154	75
penbutolol	-0.257	3	-0.772	0.02426	0.0296	66
hydrochlorothiazide	-0.393	5	-0.774	0.00106	0	80
CAY-10397	-0.51	3	-0.781	0.02141	0.0111	100
ifosfamide	-0.463	3	-0.803	0.01548	0.0182	100
ajmaline	-0.496	3	-0.832	0.00937	0.0284	100
Y-27632	-0.474	2	-0.851	0.04436	0.082	100
AH-6809	-0.546	2	-0.877	0.03024	0.0279	100
methocarbamol	-0.647	3	-0.902	0.00178	0	100
